# Fitness factor genes conserved within the multi-species core genome of Gram-negative Enterobacterales species contribute to bacteremia pathogenesis

**DOI:** 10.1371/journal.ppat.1012495

**Published:** 2024-08-23

**Authors:** Harry L. T. Mobley, Mark T. Anderson, Bridget S. Moricz, Geoffrey B. Severin, Caitlyn L. Holmes, Elizabeth N. Ottosen, Tad Eichler, Surbhi Gupta, Santosh Paudel, Ritam Sinha, Sophia Mason, Stephanie D. Himpsl, Aric N. Brown, Margaret Gaca, Christina M. Kiser, Thomas H. Clarke, Derrick E. Fouts, Victor J. DiRita, Michael A. Bachman

**Affiliations:** 1 Department of Microbiology and Immunology, University of Michigan Medical School, Ann Arbor, Michigan, United States of America; 2 Department of Pathology, University of Michigan Medical School, Ann Arbor, Michigan, United States of America; 3 Department of Microbiology and Molecular Genetics, Michigan State University College of Natural Sciences, East Lansing, Michigan, United States of America; 4 J. Craig Venter Institute, Rockville, Maryland, United States of America; University of Utah, UNITED STATES OF AMERICA

## Abstract

There is a critical gap in knowledge about how Gram-negative bacterial pathogens, using survival strategies developed for other niches, cause lethal bacteremia. Facultative anaerobic species of the Enterobacterales order are the most common cause of Gram-negative bacteremia, including *Escherichia coli*, *Klebsiella pneumoniae*, *Serratia marcescens*, *Citrobacter freundii*, and *Enterobacter hormaechei*. Bacteremia often leads to sepsis, a life-threatening organ dysfunction resulting from unregulated immune responses to infection. Despite a lack of specialization for this host environment, Gram-negative pathogens cause nearly half of bacteremia cases annually. Based on our existing Tn-Seq fitness factor data from a murine model of bacteremia combined with comparative genomics of the five Enterobacterales species above, we prioritized 18 conserved fitness genes or operons for further characterization. Mutants were constructed for all genes in all five species. Each mutant was used to cochallenge C57BL/6 mice via tail vein injection along with each respective wild-type strain to determine competitive indices for each fitness gene. Five fitness factor genes, when mutated, attenuated mutants in four or five species in the spleen and liver (*tatC*, *ruvA*, *gmhB*, *wzxE*, *arcA*). Five additional fitness factor genes or operons were validated as outcompeted by wild-type in three, four, or five bacterial species in the spleen (*xerC*, *prc*, *apaGH*, *atpG*, *aroC*). Overall, 17 of 18 fitness factor mutants were attenuated in at least one species in the spleen or liver. Together, these findings allow for the development of a model of bacteremia pathogenesis that may include future targets of therapy against bloodstream infections.

## Author summary

Frequent cases of bacteremia plague our ICUs, bone marrow transplant units, and inpatient facilities. Nearly half of these infections are caused by Gram-negative bacteria. The Enterobacterales order including *E*. *coli*, *K*. *pneumoniae*, *S*. *marcescens*, *C*. *freundii*, and *E*. *hormaechei* are leading causes of bacteremia. An alarming proportion of these are due to antibiotic-resistant isolates, which are four times more likely to kill than antibiotic-susceptible isolates. Clearly, we need new therapeutic targets to treat cases of bacteremia and sepsis. Previously, it has been unclear what genes contribute to their ability to survive in this hostile host environment. We have previously undertaken unbiased genetic screens to identify genes shared by all five bacterial genera that are required for maintaining bacterial burden in the blood and blood-filtering organs. These include genes that encode proteins that maintain proton motive force, resist antimicrobial peptides and complement, mediate genome maintenance, transport key metabolites and proteins, avoid oxidative stress, acquire iron, and regulate key pathways. Mutants, constructed in these shared genes in the five species, were validated for a high proportion of genes as critical for infection in the mouse model of bacteremia.

## Introduction

Sepsis, a life-threatening organ dysfunction, results from an unregulated immune response to infection. It is the leading cause of death in hospitalized patients across the United States [1] with a mortality rate of 25–50% leading to 220,000 deaths per year. The leading causes of bloodstream infections include the bacterial pathogens *Escherichia coli*, *Staphylococcus aureus*, *Klebsiella pneumonia*e, and coagulase-negative Staphylococci. In addition, other notable pathogens include *Pseudomonas aeruginosa*, *Acinetobacter baumannii* and *Candida* species [2,3]. However, Gram-negative pathogens cause nearly half of bacteremia cases annually [4] and these isolates are increasingly antibiotic-resistant [4,5]. Indeed, facultatively anaerobic species within the Enterobacterales order are the most common cause of Gram-negative bacteremia, including the species *Escherichia coli*, *Klebsiella pneumoniae*, *Serratia marcescens*, *Citrobacter freundii* [6] and *Enterobacter hormaechei* [7]. Early treatment with antibiotics is critical to reduce mortality, but antibiotic resistance may thwart this empiric therapy. Thus, there is a critical need to develop new therapies and salvage existing ones to counter antibiotic resistance and reduce sepsis mortality.

Bacteremia has three phases of pathogenesis: initial primary site infection, dissemination to the bloodstream, and growth and survival in blood and blood-filtering organs [8]. In Gram-negative bacteremia, the primary site serves as a reservoir of the pathogen that can intermittently re-seed the bloodstream and prolong the infection. We have recently determined that Enterobacterales replicate rapidly in the liver and spleen during bacteremia [9], but are slowly cleared in most cases, indicating that the immune system can overcome rapid bacterial growth. Whereas current antibiotics are based on the ability to kill or inhibit bacterial growth *in vitro*, there is an opportunity to identify novel drug targets that contribute to the persistence of infection.

To that end, we previously constructed random transposon libraries or ordered transposon libraries in representative clinical isolates of five Gram-negative Enterobacterales species: *E*. *coli* CFT073 [10], *K*. *pneumoniae* KPPR1 [11,12], *S*. *marcescens* UMH9 [13], *C*. *freundii* UMH14 [14], and *E*. *hormaechei* UM_CRE_14. We conducted global Tn-Seq screens in the murine model of bacteremia by tail vein inoculation and identified putative fitness genes of each species predicted to contribute significantly to maintaining the bacterial burden in the bloodstream and blood-filtering organs including the spleen and liver. The characteristics of the Tn libraries and the outcomes of the Tn-Seq screens are summarized in **Table 1**.

**Table 1:** Species, strains, transposon pool complexity, and genome sequences used in Tn-Seq studies in the murine bacteremia model.

Species and strain	No. mutants in transposon pool	No. fitness genes predicted by Tn-Seq	Fitness genes validated^a^	Genome size (Mb) (species range)	Pan genome analysis		Reference
					No. genomes sequenced by us	No. genomes in global database	
*E*. *coli* CFT073	360,000	242	9/11	5.2 (4.9–5.4)	2	9904	[10,15]
*K*. *pneumoniae* KPPR1	25,000	58	8/8	5.4 (4.2–6.1)	117	3517	[11,16]
*C*. *freundii* UMH14	44,000	223	5/7	4.9 (4.9–5.8)	8	102	[14]
*S*. *marcescens* UMH9	32,000	169	6/7	5.0 (4.9–5.4)	11	358	[13]
*E*. *hormaechei* UM_CRE_14	15,000	214	5/7	5.0 (5.1–5.6)	90	593	-^b^

^a^number of independently constructed fitness factor mutants that were validated in murine model of bacteremia per number of fitness factors tested in the original studies for each species.

^b^*E*. *hormaechei* UM_CRE_14 shared all Prioritized Fitness Genes listed in Tables 3 and 4 with the exception of *arnBCADTEF*.

To further enable these studies, we also conducted extensive genomic comparisons and identified the multi-species core genome of Enterobacterales species commonly causing bacteremia in humans [17]. By integrating our multi-species core genome and genome-wide fitness data of the five selected species, we aligned Tn-Seq screen hits identified in a murine model of bacteremia to prioritize fitness genes shared among Enterobacterales species.

Although phenotypically similar in terms of antimicrobial resistance and metabolism, these five Gram-negative species nevertheless represent a heterogeneous group of organisms that differ in virulence mechanisms, primary sites of infection, and metabolic pathways. For example, extraintestinal pathogenic *E*. *coli* produce a a variety of adhesins and toxins and generally reaches the bloodstream by infection of the urinary tract, ascension to the kidneys, and breaching of the capillary network in the kidney. *K*. *pneumoniae* commonly infects the lungs causing a highly inflammatory pneumonia relying on multiple capsule types and siderophores, and disseminates from the lungs to the bloodstream. *Serratia marcescens* has a propensity for kidney infection and can enter the circulation by crossing those endothelial barriers. *C*. *freundii* and *E*. *hormaechei* colonize the gastrointestinal tract and disseminate from there [for a review, see [2]].

There is also wide variation in our knowledge regarding infections of the bloodstream. For example, *E*. *coli* has been extensively studied in the context of extraintestinal infection (~6000 PubMed references on “*E*. *coli* and bacteremia” as of 2024) and ~2000 for *K*. *pneumoniae*. In contrast, our understanding of *S*. *marcescens*, *C*. *freundii*, and *E*. *hormaechei* has lagged behind, despite the recognition of their epidemiological importance (just over 300 references combined). Moreover, only a small percentage of these reports dealt with virulence mechanisms, but rather characterized clinical infections and antibiotic resistance. While previous studies have evaluated Gram-negative bacteremia fitness genes required in the blood, there has been no systematic analysis of shared genes critical across species in this hostile environment. Based on the data herein, we developed a model of Enterobacterales pathogenesis of bacteremia for these species.

The goal of this study was to identify and characterize conserved bacterial fitness genes and operons that play critical roles in the development and outcome of bacteremia that may also serve as potential therapeutic targets. Data from independent Tn-Seq studies of five Enterobacterales species in a murine model of bacteremia were used to prioritize conserved fitness factor genetic components. Mutants in 18 shared genetic components were newly constructed in all five species and used to cochallenge C57BL/6 mice via tail vein injection along with the respective wild-type strain of each species to determine competitive indices for each fitness gene or operon. Twelve of 18 prioritized components were confirmed as fitness factors in three or more species. Relevant phenotypes of the mutants were assessed to validate the mutant constructs and identify potential *in vitro* correlates of virulence. We propose that these genes and their respective proteins may represent future targets of therapy against bloodstream infections.

## Results

### Selection and mutation of fitness genes found in the multi-species core genome shared by at least four of the five species

In preparation for this study, we defined a “multi-species core genome” composed of 2850 non-essential genes shared by at least four of the five species as detailed in Materials and Methods (Determination of the multi-species core genome) and by [17]. Using the Tn-Seq data from transposon libraries of the Gram-negative species used to challenge our murine model via tail injection, we found 500 total bacteremia fitness genes present in at least one species-level core-genome of which 373 were represented in the multi-species core genome. From the 373 total bacteremia fitness genes, we created a scoring rubric to prioritize conserved fitness genes of interest for further study as detailed in Materials and Methods (Scoring rubric for ranking and prioritization of fitness mutants) and as outlined in [17]. Scoring for each fitness gene was based on the following criteria 1) the magnitude of the fitness defect associated with a gene in any one species, 2) a gene that was a fitness factor in multiple species, 3) whether multiple fitness genes reside in the same operon, and 4) that mutation of a fitness gene was found to confer increased antibiotic susceptibility in *E*. *coli* BW25113 [18,19] (seven different antibiotics were tested; however, cephalosporins, antibiotics clinically useful against bacteremia, were not tested in these published studies). 102 of the 373 fitness factor genes met this last criterion, including prioritized genes *atpG*, *ubiG*, *hscB*, *gmhB*, *sapA*, *pstC*, *tatC*, *ruvA*, *xerC*, and *lpdA*. Overall, 18 single genes and/or gene clusters within operons, ranked according to the scoring rubric, were prioritized for study based on their initial scoring (**Table 2**). These conserved and shared bacteremia fitness genes, predicted by Tn-Seq screens to contribute significantly to virulence of Gram-negative Enterobacterales bacilli, were assigned to eight common pathways.

**Table 2:** Prioritization criteria for conserved and shared bacteremia fitness genes within common pathways predicted by Tn-Seq screens as required for virulence of Gram-negative Enterobacterales bacilli.

Bacterial Cell Function	Scoring Rubric Score				Phenotypes of mutants assessed^f^
**1. Maintenance of Proton-Motive Force across inner membrane**	**Gene** ^**a**^	**Score**	**Operon** ^**b**^	**Score**	
ATP Synthase	*atpG*	9	*atpI* ***BE*** *F* ***HAGD*** *C*	40	
Electron Transport: Ubiquinone synthesis	*ubiH*	5	***ygfB pepP ubiHI***	14	
Iron-Sulfur Cluster biosynthesis for cytochrome maturation	*hscB*	10	*iscR****SUA*** *hsc****BA*** *fdx iscX*	36	
**2. Resistance to Antimicrobial Peptides and Complement**					
Modification of Lipid A of LPS	*arnD*	12	*arnBC* ***ADTEF***	34	**+**
LPS inner core synthesis	*gmhB*	7	gmh**B**	n/a^c^	**+**
Synthesis of Enterobacterial Common Antigen	*wzxE*	13	*wec****A*** *wzz****E*** *wec****B****C rffG****H*** *wecD****E*** *wzx****E*** *wec****F*** *wzyE rff****M***	59	+
Sensitivity to antimicrobial peptides	*sapA*	8	*sap* ***A*** *B* ***CDF***	19	**+**
Periplasmic protease inactivates complement	*prc*	6	*pro* ***Q prc***	16	**+**
**3. Transport**					
Phosphate (ABC) transport	*pstC*	10	*pst* ***SCA*** *B* ***phoU***	31	**+**
Twin arginine transporter	*tatC*	9	*tatAB* ***CD***	11	**+**
**4. Genome maintenance**					
Endonuclease that resolves Holliday junctions	*ruvA*	10	ruv**AB**	12	**+**
Tyrosine recombinase	*xerC*	10	*yif****L*** *dap****F*** *yig****A*** *xer****C*** *yigB*	24	**+**
**5. Shikimate biosynthesis**					
Contributes chorismate for quinone, siderophore, aromatic amino acid, and folate biosynthesis	*aroK* ^*e*^	6	*aro****KB*** *dam****X dam*** *rpe gph trpS*	25	**+**
**6. Global gene regulation**					
Aerobic respiration control protein	*arcA*	5	arc**A**	n/a^c^	**+**
**7. Osmotic and Oxidative stress**					
Regulates Import of proline/betaine by ProP	*proQ* ^*d*^	10	*pro* ***Q prc***	16	**+**
Oxidative stress response	*apaG* *pdxA*	4 8	*lptD* ***surA pdxA rsmA*** *apa****GH*** *(same as above)*	31 31	**+**
**8. Metabolic Pathways**					
Pyruvate dehydrogenase complex component	*lpdA*	10	*pdhR ace* ***E*** *F lpd* ***A***	17	

^a^individual gene score from scoring rubric (see Materials and Methods and [17].

^b^score from the scoring rubric of the operon containing the highlighted gene(s); **bold** indicates that a gene was identified as a fitness factor in at least one of the five bacterial species in Tn-Seq studies (**Table 1**). Operon gene order is representative for the five species; however, gene order may differ in some species.

^c^n/a, not applicable; gene not found within an operon

^d^because of concerns that a *proQ* mutant would introduce polar effects on the downstream *prc* gene, *proP*, which encodes the proline/betaine importer that is regulated by *proQ*, was chosen to construct the mutant used in this study.

^e^the *aroC* allele, chorismate synthase, encodes the terminal enzyme in the shikimate biosynthesis pathway and was selected for mutagenesis in this study.

^f^Phenotypes tested for mutants are cited in **S2 Table** where statistically significant changes in phenotypes associated with each mutant as compared to wild-type are listed for all five species.

**Fig 1:**
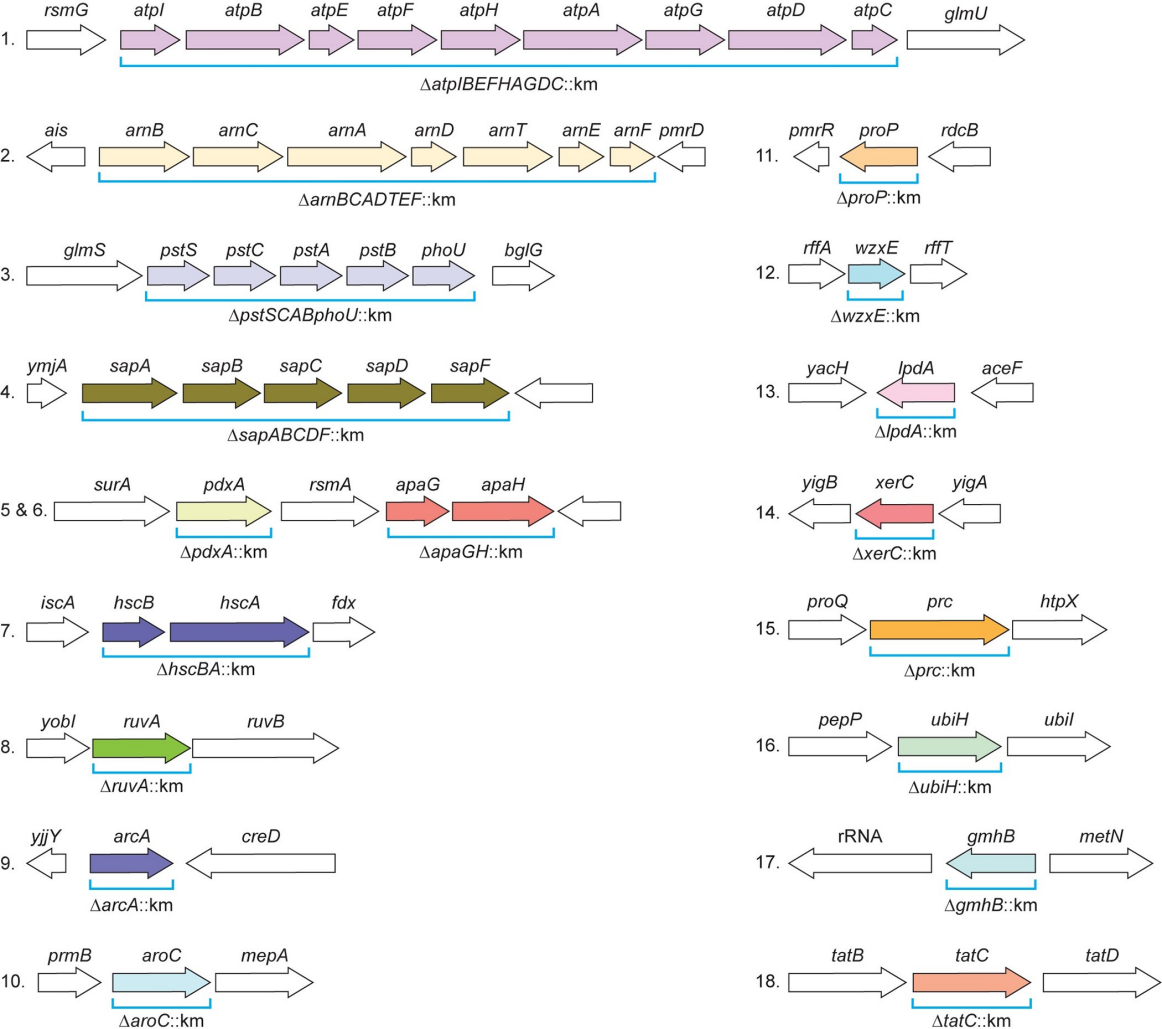
Prioritized multi-species bacteremia fitness genes. Colored arrows represent fitness genes that were targeted for mutagenesis via recombineering. The genetic organization of *E*. *coli* CFT073 is shown for reference, but corresponding mutations were generated in each of the five species of interest. ORF length are not to scale.

All isolates representing the five species (**Table 1**) have been demonstrated as amenable to *lambda* red recombineering (see “Construction of mutants in prioritized fitness genes” in Materials and Methods). Therefore, using a common strategy outlined in **Fig 1**, we constructed 89 total deletion mutants composed of either single or multi-gene (*i*.*e*., operons) mutations covering 203 genes in total across all five species. In *E*. *coli*, *K*. *pneumoniae*, *S*. *marcescens*, and *C*. *freundii*, we constructed 18 mutants that covered 42 total genes per species. In *E*. *hormaechei* UM_CRE_14, a strain that lacks *arn* genes, we constructed 17 mutants that covered 35 genes. All mutant constructs were verified by PCR and/or sequencing.

### Assessment of virulence of mutants versus wildtype strains in the murine tail vein injection model

Each of the 89 mutants were competed head-to-head against their respective wild-type strains in co-infections in the murine bacteremia model. Inocula for each species were titrated to avoid lethality and were set to represent maximal CFU/ml that would not result in loss of the murine host following inoculation (see footnote “a” in Table 3 for inocula size for each species). At 24 hours post-inoculation, mice were euthanized and CFU/g tissue in homogenates of spleens and livers were enumerated and used to calculate competitive indices. Results are shown for all five species in **Table 3** for spleens and **Table 4** for livers. Statistical corrections for multiple comparisons were implemented by determining the False Discovery Rates for each species; P values were adjusted accordingly (see footnote “b” in Tables 3–5). Competitive indices +/- standard deviations in spleens and livers are presented in graphical form in **S1 Fig**. Average fold-defects +/- standard deviations are presented for these data in **S2 Fig**. We additionally report competitive indices of *S*. *marcescens* UMH9 mutants in kidneys (**Table 5**) owing to the wild-type’s robust capacity to colonize this organ in this murine model [9]. Competitive indices +/- standard deviations in spleens and livers are presented in graphical form in **S3 Fig**. Individual mutant competitive indices found to be statistically below the hypothetical mean of 0 (*i*.*e*., neutral fitness) are considered attenuated (green shading).

**Table 3:** Competitive indices of bacteremia Tn-Seq fitness factor gene mutants in the Spleen following cochallenge via tail vein injection with respective wild-type parent strain in the murine model of bacteremia.

Prioritized fitness factor gene	*E*. *coli* CFT073	*K*. *pneumoniae KPPR1*	*C*. *freundii* UMH14	*S*. *marcescens* UMH9	*E*. *hormaechei UM_CRE_14*
	**Log**_**10**_ **Competitive Index**^**a**^ **(*p* value**^**b**^**)**^**c**^				
*tatC*	-0.440* ^**d**^	-1.725**	-1.772*** ^**g**^	-2.187**	-0.843**
*ruvA*	-1.191*	-1.331*	-1.019***	-0.675***	-1.533*
*xerC*	-0.438*	-0.747**	-0.533***	-0.556*	-1.512*
*gmhB*	**-1.017** ^**e**^	-0.800* ^**e**^	-0.293*** ^**e**^	-0.954**	-1.326**
*wzxE*	-0.903*	-1.105*	**0.063**	-1.077***	-0.750**
*arcA*	**-0.154** ^**f**^	-0.697* ^**f**^	-0.407* ^**f**^	-0.279* ^**f**^	-1.046**
*prc*	-0.482*	0.730*	-0.222***	-0.580***	-0.395**
*apaGH*	**0.058**	-0.519*	-0.349**	-0.261*	**-0.518**
*atpIBEFHAGDC*	-0.6306*	-1.531**	**0.140**	-0.566**	**-0.506**
*aroC*	**-0.018**	**-0.092**	-0.564***	-0.351***	-0.519**
*ubiH*	**-0.038**	**-0.496**	**-0.093**	-0.649*	-0.883**
*pdxA*	**-0.261**	-0.356* ^**i**^	**-0.118**	**-0.084**	-0.392*
*hscBA*	-0.299*	-0.377*	**0.570**	**-0.032**	**0.288**
*pstSCABphoU*	-0.387*	**-0.031**	**-.0118**	**-0.104**	-0.568*
*lpdA*	**-0.824**	**-0.674**	**-0.251**	-0.557*	**-1.093**
*sapABCDF*	**0.015**	-0.924*	**0.032**	**0.391**	**-0.222**
*arnBCADTEF*	**-0.275**	**0.060**	**-0.235**	**-0.0445**	**-** ^**h**^
*proP*	**-0.057**	**0.170**	**0.174**	**0.0048**	**0.060**

^a^Competitive index (CI) was calculated as (CFU_mutant_/CFU_wild-type_)^output^/(CFU_mutant_/CFU_wild-type_)^input^ Inocula in total CFU were *E*. *coli* CFT073 (1 x 10^7^), *K*. *pneumoniae* KPPR1 (1 x 10^5^), S. *marcescens* UMH9 (5 x 10^6^), *C*. *freundii* UMH14 (1 x 10^8^), and *E*. *hormaechei* UM_CRE_14 (1 x 10^8^). Competitive indices were derived from pools of 5–14 mice.

^b^CI data subjected to *t*-test for determination of statistical significance. Data were recalculated for False Discovery Rate and p values were adjusted for multiple comparisons: *p ≤ .05, **p ≤ .01, ***p ≤ .001

^c^number of mice co-challenged by tail vein injection with wild-type and mutant were 5–14 mice. CFU determined at 24 hours

^d^Green shading, wild-type significantly outcompeted mutant; No shading, wild-type did not significantly outcompete mutant. Brown shading, mutant significantly outcompeted wild-type strain. Genes are listed in order of number of species in which the gene was validated as a fitness gene and then by the magnitude of fitness defect in validated genes.

^**e**^*E*. *coli gmhB* competitive index derived from *gmhB*::Tn5 library [16]. *K*. *pneumoniae* and *C*. *freundii gmhB* competitive indices have been reported in our published work [14].

^f^*arcA* competitive indices have been reported in our published work [20].

^g^*tatC* competitive indices have been reported in our published work [14].

^h^*arn* genes not present in *E*. *hormaechei*

^i^*pdxA* competitive index and complementation for *K*. *pneumoniae* has been reported in our published work [11].

**Table 4:** Competitive indices of bacteremia Tn-Seq fitness factor gene mutants in the Liver following cochallenge via tail vein injection with respective wild-type parent strain in the murine model of bacteremia.

Prioritized fitness factor gene	*E*. *coli* CFT073	*K*. *pneumoniae KPPR1*		*C*. *freundii* UMH14	*S*. *marcescens* UMH9	*E*. *hormaechei UM_CRE_14*
	**Log**_**10**_ **Competitive Index**^**a**^ **(*p* value**^**b**^**)**^**c**^^**d**^					
*tatC*	-0.798*		-1.693*	-2.195*** ^**g**^	-2.151*	-2.141***
*gmhB*	-0.630*		-1.000** ^**e**^	-0.578* ^**e**^	-0.754*	-2.452***
*aroC*	-0.339*		-0.995**	-0.891*	-1.515*	-0.826**
*ruvA*	-1.873*		-1.507***	-1.318***	**-0.802**	-2.596***
*ubiH*	-0.430**		-1.372***	**0.285**	-3.239***	-1.611***
*arcA*	**-0.139** ^**f**^		-1.619*** ^**f**^	-0.627** ^**f**^	-0.737** ^**f**^	-1.762*
*wzxE*	-0.873*		-2.343*	**-0.547**	-1.377*	-1.505**
*xerC*	**-0.627**		-0.959**	-0.623*	**-1.045**	-1.250**
*apaGH*	**-0.180**		-1.428**	-0.484*	**-0.523**	-0.833*
*hscBA*	-0.469*		-1.515*	**0.155**	**-0.353**	-0.349*
*prc*	-0.607*		0.777**	-0.918*	**-0.726**	**-0.246**
*lpdA*	-1.195*		**-0.835**	**0.496**	**-0.982**	-2.233**
*pstSCABphoU*	**-0.995**		**-0.853**	**-0.215**	**-0.416**	-2.628**
*atpIBEFHAGDC*	**-0.759**		**-2.039**	**0.069**	**0.080**	-1.474*
*pdxA*	**-1.260**		**-0.363** ^**i**^	**0.227**	**-0.019**	-0.440*
*proP*	-0.347*		0.666**	**-0.934**	**0.117**	**-0.093**
*arnBCADTEF*	**-0.153**		**-0.203**	**-1.108**	**-0.066**	**-** ^**h**^
*sapABCDF*	**0.000**		**-0.133**	**-0.112**	**-0.191**	**-0.003**

^a^Competitive index (CI) was calculated as (CFU_mutant_/CFU_wild-type_)^output^/(CFU_mutant_/CFU_wild-type_)^input^. Inocula in total CFU were *E*. *coli* CFT073 (1 x 10^7^), *K*. *pneumoniae* KPPR1 (1 x 10^5^), S. *marcescens* UMH9 (5 x 10^6^), *C*. *freundii* UMH14 (1 x 10^8^), and *E*. *hormaechei* UM_CRE_14 (1 x 10^8^).

^b^CI data subjected to *t*-test for determination of statistical significance. Data were recalculated for False Discovery Rate and p values were adjusted for multiple comparisons: *p ≤ .05, **p ≤ .01, ***p ≤ .001

^c^number of mice co-challenged by tail vein injection with wild-type and mutant were 5–14 mice. CFU determined at 24 hours

^d^ Genes are listed in order of number of species in which the gene was validated as a fitness gene and then by the magnitude of fitness defect in validated genes. Green shading, wild-type significantly outcompeted mutant; No shading, wild-type did not significantly outcompete mutant. Brown shading, mutant significantly outcompeted wild-type strain.

^**e**^*E*. *coli gmhB* competitive index derived from *gmhB*::Tn5 ordered library [11]. *K*. *pneumoniae* and *C*. *freundii gmhB* competitive indices have been reported in our published work [14,16].

^f^*arcA* competitive indices for have been reported in our published work [20].

^g^*tatC* competitive indices have been reported in our published work [14].

^h^*arn* operon not found in *E*. *hormaechei*

^i^*pdxA* competitive index for *K*. *pneumoniae* has been reported in our published work [11].

**Table 5:** Competitive indices of prioritized Tn-Seq fitness factor gene mutants in the Kidneys following cochallenge with wild-type *Serratia marcescens* UMH9 in the murine model of bacteremia.

Prioritized fitness factor gene	Log_10_ Competitive Index^a^ (*p* value^b^)^c^	Fold-fitness Defect
*aroC*	-2.669**^d^	**-467**
*wzxE*	-2.478**	**-301**
*tatC*	-2.466**	**-292**
*atpIBEFHAGDC*	-2.143**	**-139**
*prc*	-1.260**	**-18**
*arcA*	-1.201**	**-16**
*lpdA*	-1.678*	**-48**
*ubiH*	-1.500*	**-32**
*apaGH*	-1.071*	**-12**
*xerC*	-0.786*	**-6.1**
*pdxA*	-0.723*	**-5.3**
*gmhB*	**-0.931**	**-8.5**
*arnBCADTEF*	**-0.238**	**-1.7**
*pstSCABphoU*	**-0.207**	**-1.6**
*ruvA*	**-0.152**	**-1.4**
*hscBA*	**-0.083**	**-1.2**
*sapABCDF*	**0.011**	**-1.0**
*proP*	**1.337**	**22**

^a^ Competitive index (CI) was calculated as (CFU_mutant_/CFU_wild-type_)^output^/(CFU_mutant_/CFU_wild-type_)^input^

^b^CI data subjected to *t*-test for determination of statistical significance. Data were recalculated for False Discovery Rate and p values were adjusted for multiple comparisons:*p ≤ .05, **p ≤ .01,***p ≤ .001

^c^number of mice co-challenged by tail vein injection with wild-type and mutant were 5–14 mice per cochallenge. CFU determined at 24 hours

^d^Green shading, wild-type significantly outcompeted mutant; No shading, wild-type did not significantly outcompete mutant. Genes are listed in order of the magnitude of fitness defect in validated genes.

In the spleen (**Table 3**), three mutants (*tatC*, *ruvA*, *xerC*) were attenuated in all five species. Four mutants (*gmhB*, *wzxE*, *arcA*, *prc*) were attenuated in four species. Seven mutants (*apaGH*, *atpIBEFHAGDC*, *lpdA*, *aroC*, *ubiH*, *pdxA*, *hscBA*, *pstSCABphoU*) were outcompeted by wild-type in two or three bacterial species. Two mutants were outcompeted in only one species (*lpdA*, *sapABCDF*) and two mutants (*arnBCADTEF*, *proP*) were not attenuated in any species.

In the liver (**Table 4**), three mutants (*tatC*, *gmhB*, *aroC*) were outcompeted by the wild-type of all five bacterial species. Four mutants (*ruvA*, *ubiH*, *arcA*, *wzxE*) were attenuated in four species. Four mutants (*xerC*, *apaGH*, *hscBA*, *prc)* were outcompeted by wild-type in three bacterial species. Six mutants (*lpdA*, *pstSCABphoU*, *atpIBEFHAGDC*, *pdxA proP*) were attenuated in 1 or 2 species. As for the liver, only two mutants (*arnBCADTEF*, *sapABCDF*) were not attenuated for any species.

Unlike the other four species, *S*. *marcescens* has an affinity for kidney colonization in addition to the spleen and liver following challenge by tail vein injection of mice [9]. Six of the 18 mutants (*aroC*, *wzxE*, *tatC*, *atpIBEFHAGDC*, *prc*, *arcA*) were significantly outcompeted by the *S*. *marcescens* wild-type strain in the kidneys with fitness defects ranging from 467-fold to 16-fold (**Table 5**).

### Establishing phenotypes of mutants *in vitro*

The 89 bacterial constructs carrying deletion mutations in single prioritized fitness genes or their respective operons across all species were then evaluated for *in vitro* phenotypes. (**Tables 2 and S2**). We also sought *in vitro* correlates that could predict successful colonization by a bacterial species in the murine model of bacteremia. We attempted to determine whether the functional pathways identified represent a global strategy of Gram-negative bacilli to successfully infect the bloodstream.

#### *i*. Growth rates of mutants

Growth rates of wild-type strains and all mutants were assessed during exponential growth in lysogeny broth (LB) at 37°C with aeration to identify potential growth defects in rich medium displayed by the mutants as compared to their respective wild-type strains. Growth was monitored over 16 h in automated growth curve analyzers. Relative growth rates were defined during exponential growth phase as maximal specific growth rate of mutant / maximal specific growth rate of wild-type **(Table 6** and **S4 Fig)**. For 65 of 89 (73%) mutants, no statistically significant defect in growth rate was noted as compared to the wild-type strain. Among the mutant constructs with growth defects, mutations in a component of the pyruvate dehydrogenase complex (*lpdA*) and an enzyme in the ubiquinone synthesis pathway (*ubiH*) conferred significantly slower growth in all five species. Mutations in genes encoding ATP synthase (*atpIBEFHAGDC*) and the *pstSCAB phoU* locus similarly resulted in significantly slower growth in three of the five species. Although most of the observed growth phenotypes were consistent between species, *wzxE* mutations were an exception, resulting in enhanced growth for *S*. *marcescens* but slower replication for *E*. *hormaechei*.

**Table 6:** Relative growth rates of mutants compared to wild-type strains in LB medium^a^.

Mutant	Mean Relative Growth Rate^b^ (Adj. P^c^)				
	*E*. *coli* CFT073	*K*. *pneumoniae* KPPR1	*S*. *marcescens* UMH9	*C*. *freundii* UMH14	*E*. *hormaechei* UM_CRE_14
*apaGH*	0.88 (NS)	1.06 (NS)	1.06 (NS)	0.98 (NS)	0.98 (NS)
*arcA*	(20)^d^	(20)^d^	0.79 (NS)	(20)^d^	0.51 (<0.0001)
*arnBCADTEF*	0.89 (NS)	1.09 (NS)	1.03 (NS)	1.09 (NS)	ND^e^
*aroC*	0.92 (NS)	0.66 (0.0056)	1.18 (NS)	0.99 (NS)	0.96 (NS)
*atpIBEFHAGDC*	0.68 (NS)	0.74 (0.0486)	0.62 (0.0002)	0.87 (NS)	0.75 (<0.0001)
*gmhB*	0.67 (NS)	(16)^d^	1.16 (NS)	0.78 (NS)	0.89 (0.0016)
*hscBA*	0.70 (NS)	0.89 (NS)	0.88 (NS)	1.08 (NS)	0.90 (0.0073)
*lpdA*	0.25 (<0.0001)	0.47 (<0.0001)	0.50 (<0.0001)	0.46 (0.0107)	0.31 (<0.0001)
*pdxA*	0.81 (NS)	(11)^d^	0.97 (NS)	1.17 (NS)	0.94 (NS)
*prc*	0.76 (NS)	0.97 (NS)	1.36 (0.0004)	1.03 (NS)	0.99 (NS)
*proP*	0.87 (NS)	0.97 (NS)	1.07 (NS)	0.96 (NS)	1.01 (NS)
*pstSCABphoU*	0.61 (0.0382)	0.73 (0.0346)	0.78 (NS)	0.73 (NS)	0.90 (0.0106)
*ruvA*	0.81 (NS)	1.01 (NS)	0.86 (NS)	(14)^d^	0.97 (NS)
*sapABCDF*	0.70 (NS)	0.83 (NS)	1.20 (NS)	1.02 (NS)	0.79 (<0.0001)
*tatC*	0.85 (NS)	1.04 (NS)	0.89 (NS)	(14)^d^	0.95 (NS)
*ubiH*	0.28 (<0.0001)	0.36 (<0.0001)	0.64 (0.0004)	0.43 (0.0065)	0.39 (<0.0001)
*wzxE*	0.84 (NS)	1.04 (NS)	1.60 (<0.0001)	0.90 (NS)	0.80 (<0.0001)
*xerC*	1.01 (NS)	0.97 (NS)	0.93 (NS)	1.07 (NS)	0.94 (NS)

^a^aerobic growth in lysogeny broth at 37°C

^b^maximal specific growth rate was determined using AMiGA software [21]. Relative growth rates (mutant/wild-type) <1 indicate mutant grew more slowly than wild-type.

^c^statistical significance was assessed by one-way ANOVA with Dunnett’s multiple comparisons test against a reference value of 1 representing wild-type. NS, growth rates of mutant and respective wild-type strain were not significantly different.

^d^growth rates previously determined in the indicated reference from our group. Growth rates of *arcA* mutants of *K*. *pneumoniae* KPPR1, *C*. *freundii* UMH14, and *S marcescens* UMH9 were restored to wild-type levels by complementation with *arcA in trans* on a plasmid [20].

^e^not determined, genes not present

**Fig 2:**
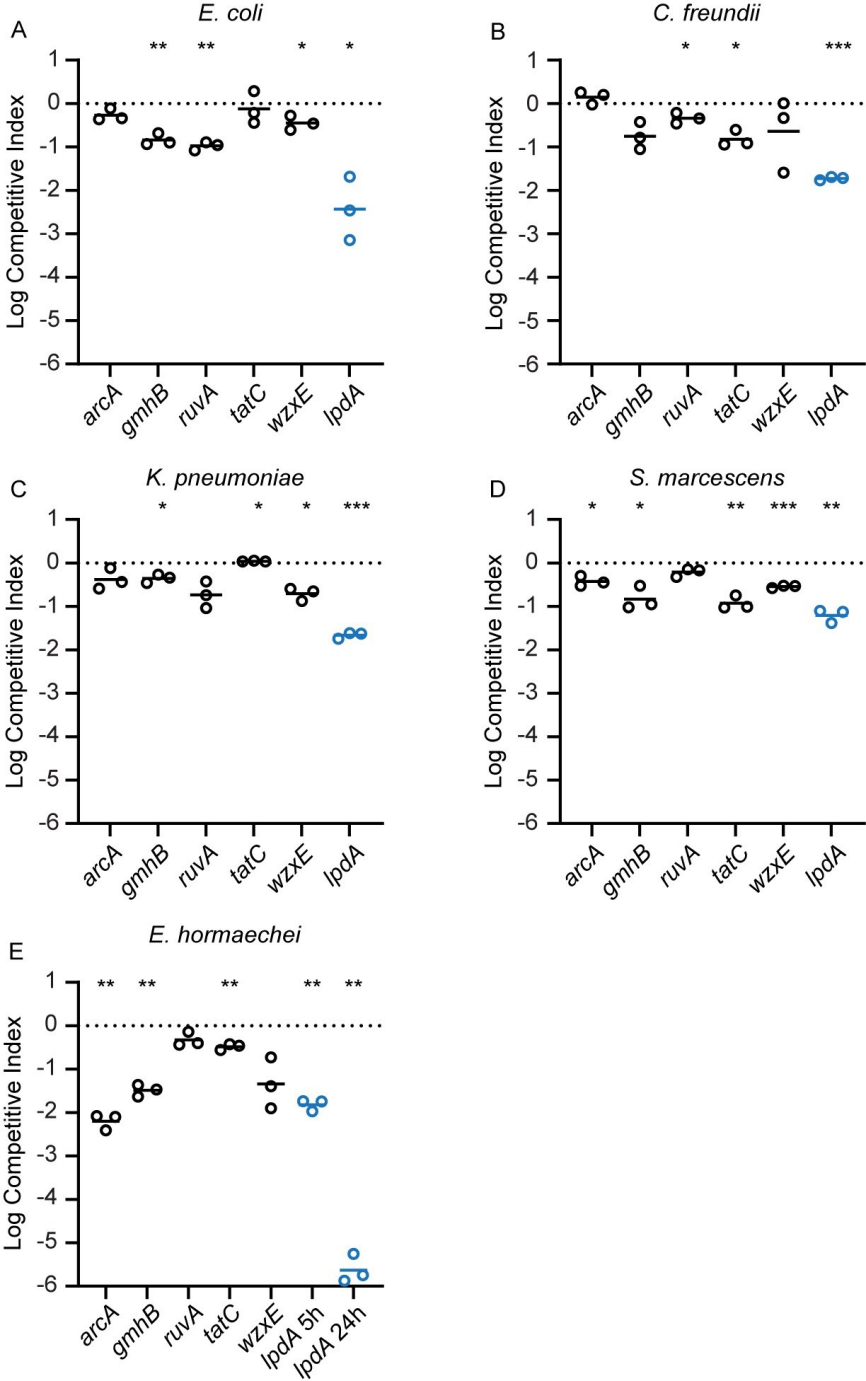
Competitive Indices of competition of mutants and respective wild type strains co-cultured in lysogeny broth. Stationary phase cultures of selected mutants and their respective wild type strains were normalized to OD_600_ = 1.0 in PBS, pH7.2, and mixed 1:1, then diluted 1:100 into 2 ml of lysogeny broth and cultured for 24 h at 37°C with aeration. Co-cultures were serially diluted and differentially plated on Luria agar with and without antibiotic selection and CFUs were quantified. Data are expressed as competitive indices as defined in footnote “a” of Table 3. Each data point represents an independent competition experiment. Significance was determined by a one-sample *t*-test performed on log_10_-transformed competitive indices (*, p < .05; **, p < .01; ***, p < .001). Blue circles indicate *lpdA* control competition experiments after 24 h with the exception of *E*. *hormaechei* which was also determined at 5 h.

To use a more sensitive method to identify potential growth defects of mutants, we conducted *in vitro* competition of mutants with respective wild-type strains by co-culture in lysogeny broth. Mutants selected were those that had not displayed a growth rate defect in mono-culture in at least four of the five species and included the top five mutants in genes that were most outcompeted by wild type in both spleen and liver (*tatC*, *ruvA*, *gmhB*, *wzxE*, and *arcA*) as well as a control, *lpdA*, which when mutated displayed a very significant growth defect in all five species. Competitive indices resulting from co-culture were more sensitive at detecting a growth defect than mono-culture (**Fig 2**). Besides all *lpdA* mutants, which had growth defects in both mono- and co-culture, nine of the 20 other mutants had growth defects in co-culture that were not detected in mono-culture. While these differences were statistically significant, generally the drop in competitive indices was less than one log_10_ and may not be sufficient to explain the full extent of the *in vivo* fitness defect.

#### *ii*. Susceptibility to Ciprofloxacin

Mutants in recombinases encoded by *ruvA* and *xerC*, responsible for DNA repair, have been shown to be susceptible to the action of the DNA topoisomerase and gyrase inhibitors including the commonly prescribed antibiotic ciprofloxacin [22]. We measured the diameters of zones of inhibition (killing) on agar plates around paper disks saturated with 5 μg ciprofloxacin. *ruvA* mutants of all five species and *xerC* mutants of four species had a significantly larger zone size (p < .05) to ciprofloxacin than the wild-type strains (**Table 7**). Notably, *E*. *hormaechei* UM_CRE_14 is completely resistant to ciprofloxacin and while mutation of *xerC* did not alter that intrinsic resistance, loss of *ruvA* did result in a significant, albeit modest, increase in susceptibility. While susceptibility to other related antibiotics was not tested, this confirms the expected *in vitro* phenotype of these mutants and their prioritization as potential fitness factors that also affect antibiotic susceptibility.

**Table 7:** Zones of inhibition for disk diffusion of ciprofloxacin in wild-type strains and *xerC* and *ruvA* mutants.

Bacterial strain	Wild-type/ mutant	Zone of Inhibition (mM)^b^	*P-value* ^c^
*E*. *coli* CFT073	wild-type	21.8	
	*xerC*	27.3	.0327
	wild-type	22.6	
	*ruvA*	29.0	.0007
*K*. *pneumoniae* KPPR1	wild-type	21.7	
	*xerC*	25.7	.0224
	*wild-type*	22.3	
	*ruvA*	27.3	.0004
*S*. *marcescens* UMH9	wild-type	22.0	
	xerC	26.7	.0002
	wild-type	21.0	
	*ruvA*	26.3	.0013
*C*. *freundii* UMH14	wild-type	22.0	
	*xerC*	30.0	.0023
	wild-type	24.0	
	*ruvA*	28.7	.0249
*E*. *hormaechei* UM_CRE_14^**a**^	wild-type	0.00	
	*xerC*	0.00	NS^d^
	wild-type	0.00	
	*ruvA*	10.7	< .0001

^a^*E*. *hormaechei wild-type* and *xerC* mutant were not susceptible to ciprofloxacin

^b^average of 3 replicates with disk containing 5 μg ciprofloxacin on agar plate

^c^Mean zone of inhibition compared using the unpaired *t-test*

^d^NS, not significant

#### *iii*. Serum Resistance

**Fig 3:**
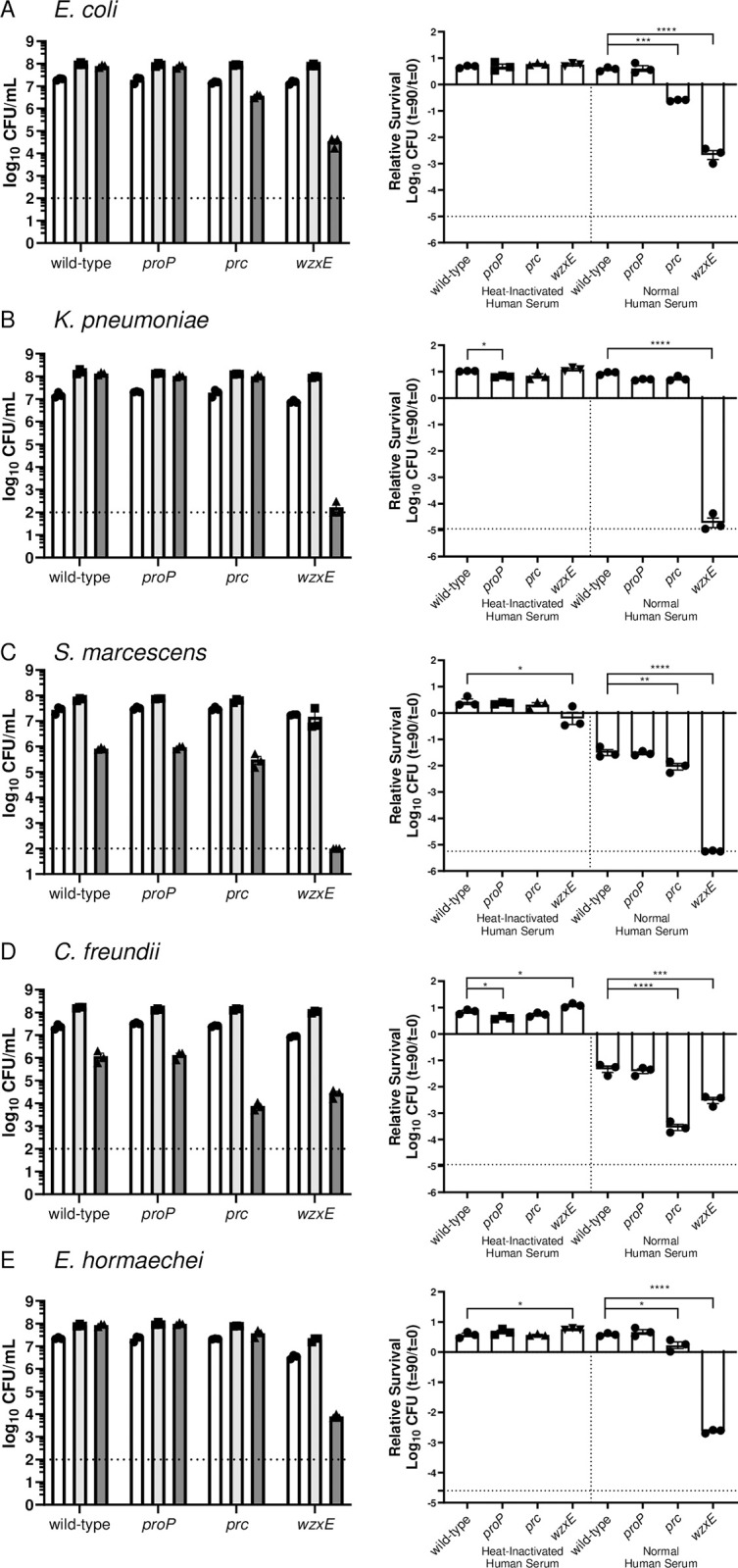
Susceptibility of Enterobacterales species to human serum. 1x10^7^ CFU/mL of bacteria (A-E: named species and strain) were incubated with normal human serum and heat-inactivated human serum to indicate complement-specific killing for 90 minutes at 37°C. 90% pooled human serum was used for *K*. *pneumoniae* KPPR1 and 40% pooled human serum for *C*. *freundii* UMH14, *E*. *coli* CT073, *S*. *marcescens* UMH9, and *E*. *hormaechei* UM_CRE_*14*. (Left panels) Individual CFUs with mean +/- SEM (n = 3) were plotted at t = 0 (white) and t = 90 in heat-inactivated human serum (light gray) and normal human serum (dark gray). (Right panels) Viability was calculated relative to t = 0 with statistical differences in susceptibility to heat-inactivated human serum or normal human serum determined using an unpaired *t*-test (*p<0.05, **p<0.01, ***p<0.001, ****p<0.0001). Dashed line denotes limit of detection. Data are presented as the mean ± SEM and are representative of 3 independent experiments each with 3 biological replicates.

We hypothesized that three fitness factor genes or operons may contribute to resistance to human serum. The *wzxE* mutants are expected to have reduced amounts of Enterobacterial Common Antigen (ECA) on the surface that may weaken outer membrane integrity and modify interaction with serum components including complement. *prc* encodes a protease involved in the regulation of peptidoglycan synthesis [23] but has also been demonstrated to degrade complement [24]. The *sap* locus encodes proteins that provide “sensitivity to antimicrobial peptides” as shown in *Salmonella* Typhimurium [25]. To test for serum sensitivity, 10^7^ cfu/ml of the wild-type strains and their respective *wzxE*, *prc*, and *sapABCDF* mutants were incubated with human serum for 90 minutes at 37°C and then plated for viability on Luria agar. *prc* deletion mutants of four species, excepting *K*. *pneumoniae* KPPR1, were found to be more sensitive to active human serum as compared to the wild-type strains (Fig 3). *wzxE* mutants in all five species were more susceptible to serum killing than their wild-type strains. None of the *proP* mutants were more sensitive than respective wild-type strains for any species tested. Mutants for all species were not sensitive to heat-inactivated human serum.

The *K*. *pneumoniae* enterobacterial common antigen (ECA) *wzxE* mutant was complemented in the human serum susceptibility assay (**S5 Fig**). As noted, the *wzxE* mutant was significantly more susceptible to killing by active human serum but not by heat-inactivated serum indicating that killing was complement-mediated. When *wzxE* mutant was complemented *in trans* on a plasmid carrying *wzxE*, killing by active human serum was reduced to the level of the wild type strain. Thus mutation of wzxE was not polar on downstream genes in the ECA operon.

#### *iv*. Susceptibility to Antimicrobial Peptides

**Fig 4:**
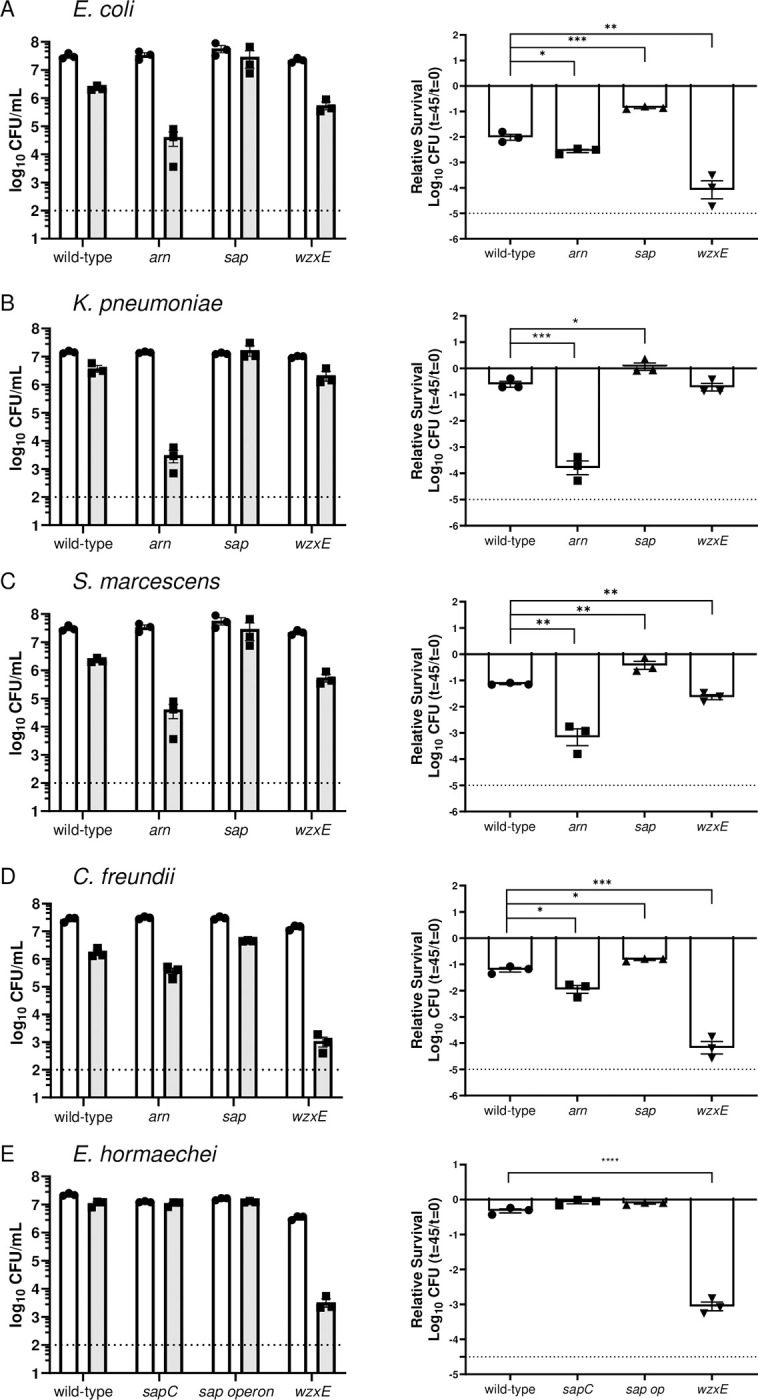
Susceptibility of Enterobacterales species to Polymyxin B. 1x10^7^ CFU/mL of bacteria (A-E: named species and strain) were incubated with Polymyxin B in PBS for 45 minutes at 37°C. (Left panels) Individual CFUs with mean +/- SEM (n = 3) were plotted at t = 0 (white) and t = 45 (gray). (Right panels) Viability was calculated relative to t = 0 with statistical differences in sensitivity to Polymyxin B determined using an unpaired *t*-test (*p<0.05, **p<0.01, ***p<0.001, ****p<0.0001). Polymyxin B concentrations used are as follows: 1 μg/mL for *E*. *coli* CFT073; 2.5 μg/mL for *C*. *freundii* UMH14; 5 μg/mL for *K*. *pneumoniae* Kppr1; 10 μg/mL for *S*. *marcescens* UMH9 and 1 μg/mL for *E*. *hormaechei* UM_CRE_14. *sap* operon indicates *sapBCADTEF* mutant. *arn* indicates *arnABCDF* mutant. Dashed line denotes Limit of Detection. Data are presented as the mean ± SEM and are representative of 3 independent experiments each with 3 biological replicates. Statistical significance was assessed by the *t*-test.

We tested *wzxE*, *arn*, and *sap* deletion mutants and their respective wild-type strains for susceptibility to the model cationic antimicrobial peptide polymyxin B (**Fig 4**). *arnBCADTEF* deletion mutants were statistically more susceptible to polymyxin B in all species tested (*E*. *hormaechei* UM_CRE_14 lacks the *arn* operon and was not tested), as were *wzxE* mutants in four species (*E*. *coli* CFT073, *C*. *freundii* UMH14, *S*. *marcescens* UMH9 and *E*. *hormaechei* UMCRE14). Unlike the published report for *Salmonella* Typhimurium [25], and opposite to the anticipated result, *sap* operon deletion mutants were statistically protected against polymyxin B relative to their respective wild-type strain in four species (*E*. *coli* CFT073, *K*. *pneumoniae* KPPR1, *S*. *marcescens* UMH9 and *C*. *freundii* UMH14). Finally, neither the *sap* operon nor the single *sapC* locus mutants in *E*. *hormaechei* UM_CRE-14 were statistically different than the wild-type strain.

#### *v*. Secretion of folded proteins

**Fig 5:**
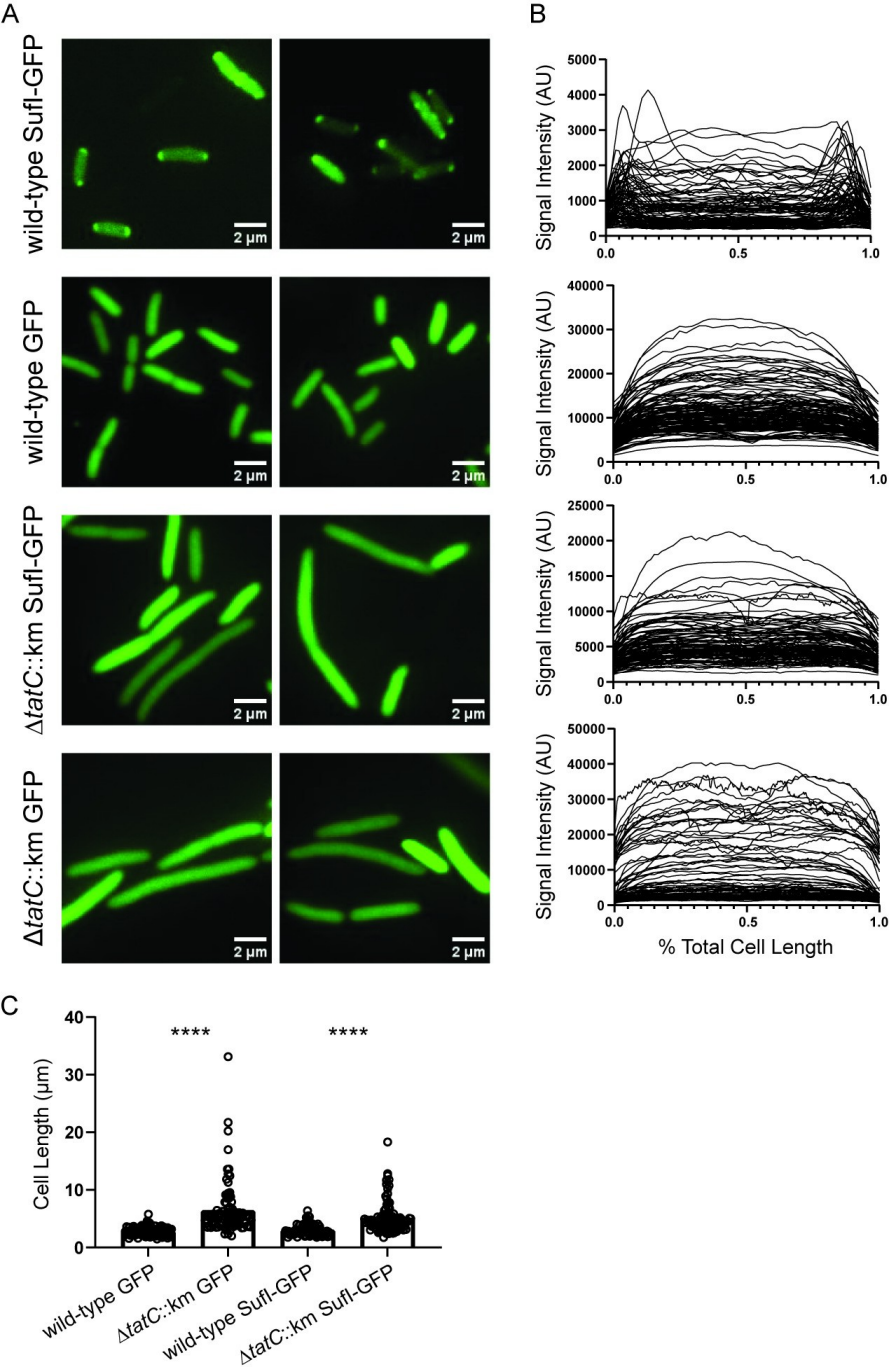
Demonstration of Tat pathway secretion in *S*. *marcescens*. A. *S*. *marcescens* wild-type and *tatC* mutant bacteria harboring plasmids that encoded an unmodified GFP (GFP) or an engineered fusion of the SufI N-terminal twin-arginine signal peptide with GFP (SufI-GFP) were visualized by fluorescence microscopy. Fluorescence intensity as a function of cell length (B) and total cell length (C) were determined for bacteria (n≥100) from multiple fields using Image J. Statistical significance for panel C was assessed by unpaired *t*-test: ****, *p*<0.0001.

SufI is a model protein substrate of the twin-arginine translocation (Tat) protein export system whose relationship with Tat secretion has been studied extensively in *E*. *coli* [26–28]. The Tat pathway is widely conserved among bacteria [29] and we have previously demonstrated that both *tatC*, encoding twin-arginine signal peptide recognition capacity, and *sufI* were significant contributors to bacteremia fitness of *C*. *freundii* [14]. Thus, we chose to demonstrate its phenotype in an uninvestigated species. In wild-type exponentially growing *S*. *marcescens* expressing a SufI-GFP fusion, a strong fluorescent signal was observed at one or both cell poles (**Fig 5A**) by fluorescence microscopy, resulting in increased signal intensity at these locations when plotted as a function of cell length (**Fig 5B**). This polar localization of SufI-GFP was completely *tatC*-dependent since fluorescence resulting from the same fusion construct was uniformly diffuse throughout the cell in the UMH9 Δ*tatC*::*km* strain. Similarly, differential localization was also not observed in wild-type or mutant bacteria expressing the unmodified GFP control plasmid, indicating that the SufI N-terminal signal sequence was required for the polar localization of SufI-GFP. Together, these results are consistent with Tat-dependent translocation of *S*. *marcescens* SufI that is mediated by the predicted twin-arginine signal sequence. In addition to the diffuse fluorescence phenotype, the Δ*tatC*::*km* mutant also exhibited a significant increase in total cell length compared to wild-type bacteria (**Fig 5C**). These findings are consistent with observations of *tat* null mutations in other species and is likely a consequence of inappropriate localization of cell division-related proteins that are secreted via the Tat pathway [27,30–32].

#### *vi*. Repression of aerobic growth

**Fig 6:**
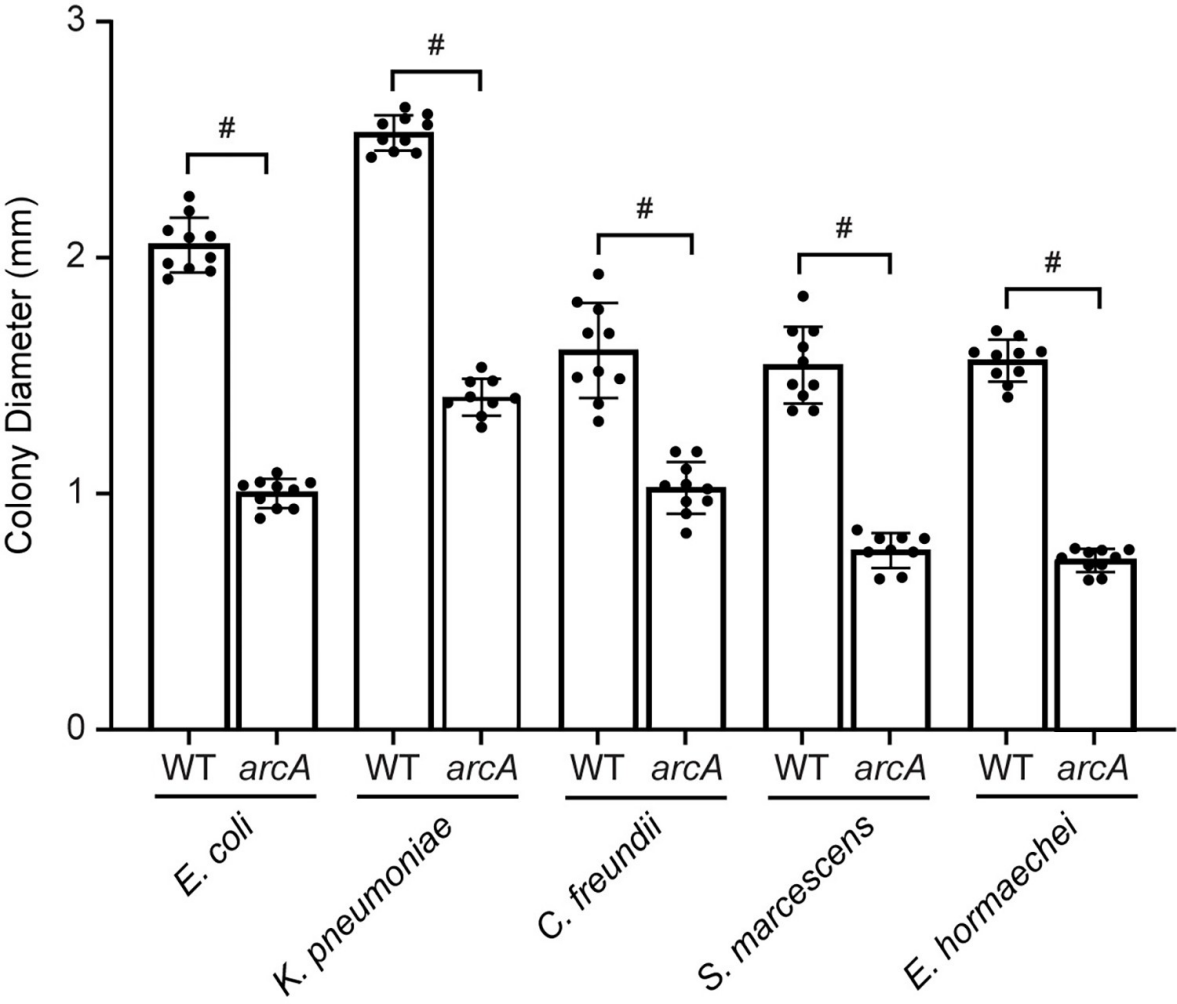
Small colony phenotype of *arcA* mutants. Wild-type and *arcA* mutants were cultured overnight in LB with aeration at 37°C. Ten-fold dilutions were spread plated onto Luria agar and incubated overnight at 37°C. Colony diameters were measured using ImageJ software (http://imagej.nih.gov/ij). ^#^For all five species, *arcA* mutants had statistically significantly smaller colony diameters than wild-type strains as determined using an unpaired *t*-test. ^#^(p < .0001). The small colony phenotype has been observed previously in *arcA* mutants [33,34].

Mutation of *arcA* in *E*. *coli*, *K*. *pneumoniae*, *S*. *marcescens*, *C*. *freundii*, *and E*. *hormaechei* resulted in a small colony phenotype compared to each respective wild-type strain, a phenotype that has been noted previously [33,34]. This was quantified by plating dilutions of cultures of *arcA* mutants and wild-type strains on Luria agar and measuring colony diameters after overnight incubation at 37°C using ImageJ-2 software (**Fig 6**). Wild-type colony diameters for *E*. *coli*, *K*. *pneumoniae*, *S*. *marcescens*, *C*. *freundii*, and *E*. *hormaechei* averaged 2.05 mM, 2.53 mM, 1.54 mM, 1.61 mM, and 1.56 mM, respectively, whereas *arcA* mutant colony diameters averaged 1.00 mM, 1.41 mM, 0.76 mM, 1.02 mM, and 0.72 mM (*p* < .0001 for all comparisons), respectively. Growth rates of the *arcA* mutants were also notably slower than respective wild-type strain when cultured aerobically in lysogeny broth [Table 6 and [20]]. Growth rates were complemented to wild type levels in lysogeny broth with *arcA* expressed *in trans* on a plasmid for *K*. *pneumoniae*, *S*. *marcescens*, and *C*. *freundii* as described previously [20]].

#### *vii*. Siderophore production

**Fig 7:**
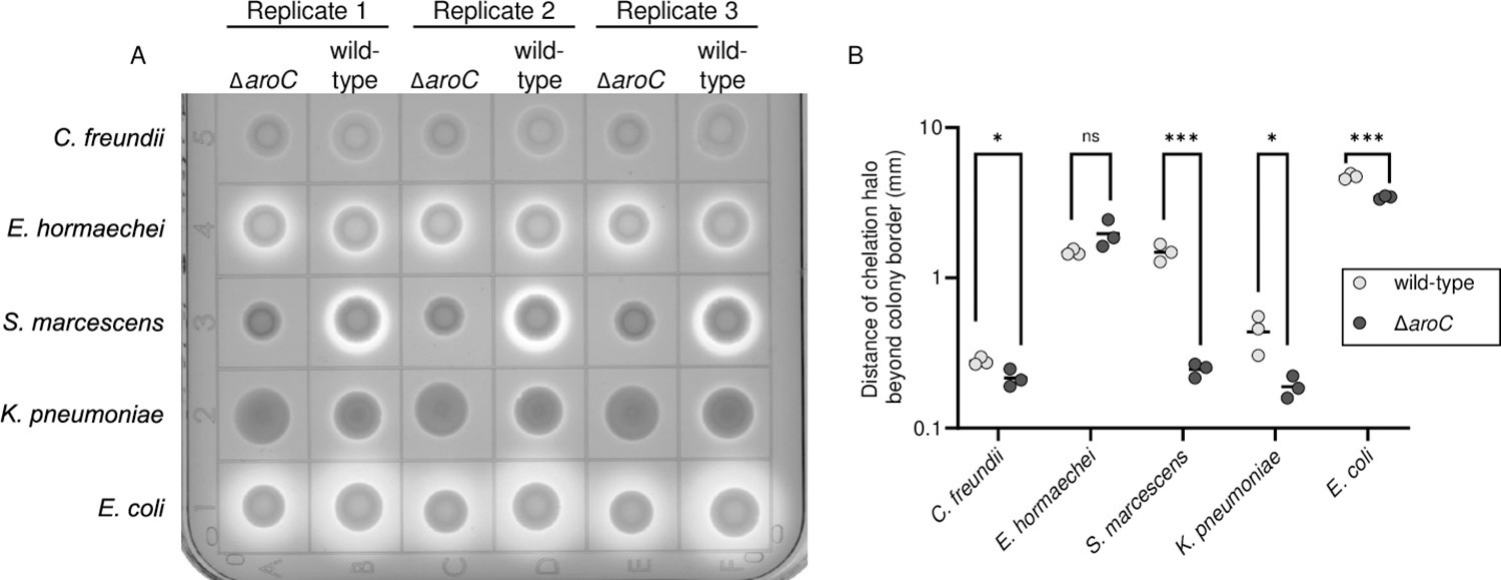
Siderophore production. (**A**) Siderophore production was detected on chrome azurol S (CAS) plates supplemented with 1% tryptone. A sample (2 μl) of overnight stationary phase LB medium cultures was spotted in triplicate onto CAS agar plates and incubated at 37°C for 16 h. (**B**) Siderophore activity was measured from the linear distance from the edge of the colony to the edge of the chelation halo at positions 12, 3, 6 and 9 o’clock (4 measurements/colony) using ImageJ software. Statistical significance is based on an unpaired two-tailed *t*-test (*p<0.05, ***p<0.005).

*aroC* encodes chorismate synthase which provides the precursor for the synthesis of catechol siderophores including enterobactin [35]. Thus, mutation of *aroC* prevents enterobactin biosynthesis. While *C*. *freundii* UMH14 synthesizes enterobactin as its only siderophore, the other four species carry multiple siderophore biosynthesis pathways. We examined total siderophore activity on CAS agar plates for wild-type strains and *aroC* mutant constructs. In the CAS assay, a bright halo around a colony, resulting from chelation of iron by the siderophore from the dye, is indicative of siderophore activity (**Fig 7A**). For four of the five species, wild-type strains quantitatively demonstrated more siderophore activity than their respective *aroC* mutants (**Fig 7B**) ranging from subtle to dramatic. For example, *S*. *marcescens* UMH9 [36], which synthesizes chrysobactin and the catecholate siderophore serratiachelin, showed a dramatic reduction in halo radius and intensity in the mutant compared to wild-type, whereas *K*. *pneumoniae* KPPR1 [37], which produces enterobactin, salmochelin, and yersiniabactin [38], and *C*. *freundii* UMH14, which synthesizes only enterobactin [assessed by antiSmash 7.0 [38]], showed a more subtle diminishment of halo intensity. For *E*. *coli* CFT073, which carries at least four siderophore biosynthetic pathways including enterobactin, salmochelin, yersiniabactin, and aerobactin as well as heme receptors [39], the loss of enterobactin, salmochelin and perhaps yersiniabactin (catechol siderophores) reduced the radius of the halo around the colony on CAS agar but the bacterium still produces significant chelating activity due to remaining siderophores. *E*. *hormaechei* UM_CRE_14, which encodes the ability to synthesize enterobactin (100% match for biosynthesis genes by antiSmash 7.0) and perhaps aerobactin (60% match) did not display a demonstrable difference between wild-type and mutant in this assay.

#### *viii*. Oxidative Stress assessed by exposure to H_2_O_2_

Resistance to oxidative stress is a key mechanism used by pathogens to evade immune responses. To determine if fitness genes annotated as involved in oxidative stress responses protected against this threat, we exposed the arcA, ruvA, and xerC mutant constructs to hydrogen peroxide (**S6 Fig**). None of these mutant constructs were more susceptible to H_2_O_2_ across any species. Previous literature demonstrated that the gene sspA is required for K. pneumoniae oxidative stress resistance [11], although this gene is not traditionally annotated as involved in this stress response [11]. Oxidative stress resistance may be conveyed by other fitness genes, and each species likely has unique mechanisms to combat this response.

#### *ix*. Osmotic stress

**Fig 8:**
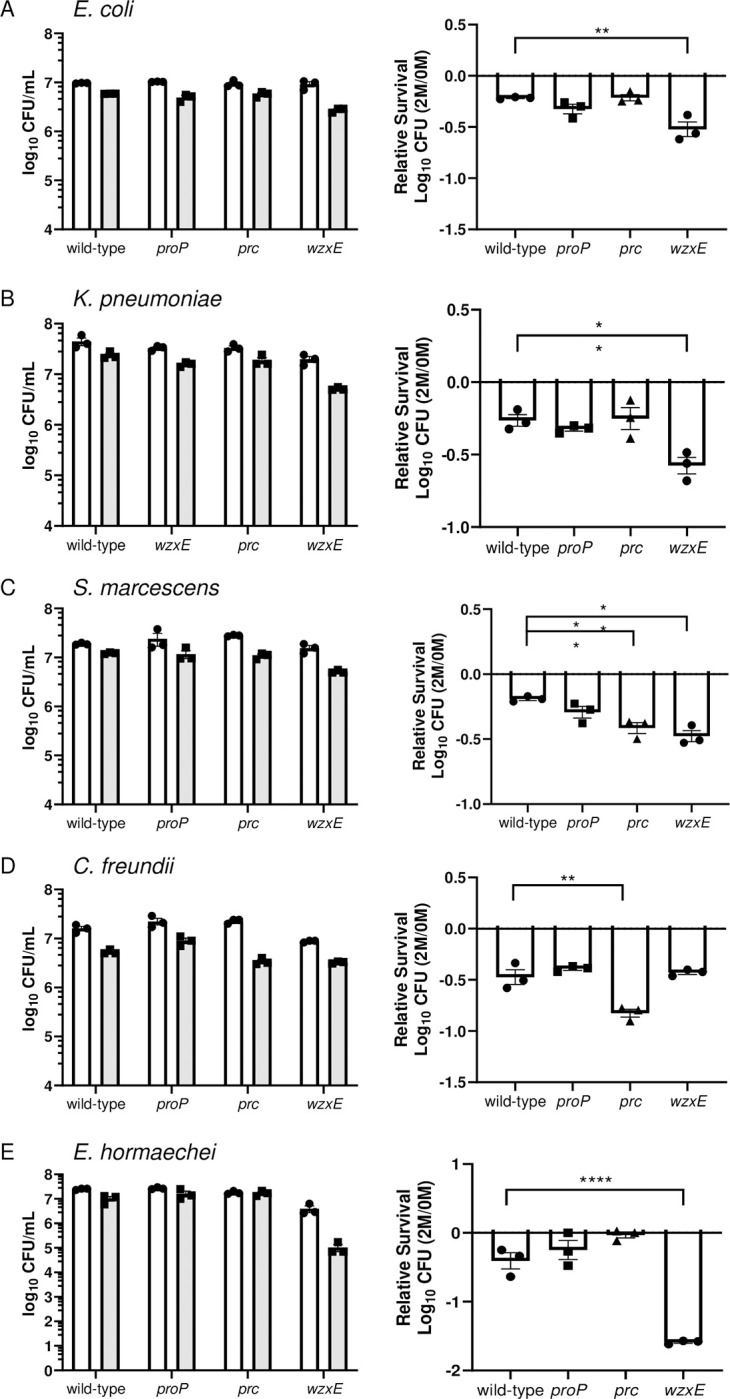
Susceptibility of Enterobacterales species to osmotic stress. 1x10^7^ CFU/mL of bacteria (A-E: named species and strain) were incubated with 0M or 2M D-sorbitol in PBS to induce osmotic stress for 30 minutes. (Left panels) Individual CFUs with mean +/- SEM (n = 3) were plotted after 30-minute incubation in 0M (white) and 2M (gray) sorbitol. (Right panels) Bacterial viability was calculated relative to 0M sorbitol. Data are presented as the mean ± SEM and are representative of 3 independent experiments each with 3 biological replicates. Statistical significance was assessed by an unpaired *t*-test.

Sensitivity to osmotic stress, encountered in the bloodstream, was assessed by incubating an independent suspension (10^7^ CFU/mL) of wild-type, *prc*, *wzxE*, and *proP* deletion mutants in PBS, pH 7.4 with and without 2 M D-sorbitol for 30 minutes at 37°C. Viable colonies were enumerated by dilution plating onto Luria agar (**Fig 8**). *prc* mutants of *S*. *marcescens* UMH9 and *C*. *freundii* UMH14 were significantly more sensitive to osmotic stress induced by 2M D-sorbitol than their wild-type counterparts. Complementation of *C*. *freundii* UMH14 *prc* mutant by *prc in trans* restored resistance to osmotic stress to wild-type levels (**S5 Fig**). *wzxE* deletions of *E*. *coli* CFT073, *K*. *pneumoniae* KPPR1, *S*. *marcescens* UMH9, and *E*. *hormaechei* UM_CRE_14 were also significantly more sensitive to sorbitol than their wild-type strains. However, no significant sensitivity in any *proP* deletion mutant to sorbitol was observed.

We also examined osmotic stress in *C*. *freundii* UMH14 by culturing the wild type strain and *prc* deletion mutant in a hypotonic medium (LB containing no NaCl). While little difference is observed in the growth patterns of the two strains in standard LB medium (which contains 1% NaCl) the *prc* mutant exhibits a severe growth defect in LB medium which does not contain NaCl (**S7 Fig**). As *prc* is preceded by *proQ* in a shared operon (Table 2) we isolated the activity of Prc from ProQ in a *prc* complementation vector by first cloning the native promoter and two-gene operon into a low-copy plasmid and then introducing a nonsense mutation in the start codon of *proQ* (pPrc). Maintenance of pPrc by the *prc* mutant restored growth in the hypotonic medium to levels comparable to that of the wild type (**S7 Fig**). This result demonstrates the hypotonic growth defect is directly attributable to the absence of the *prc* allele as it can be fully complemented by provision of *prc* on a plasmid.

#### x. Envelope stress

**Fig 9:**
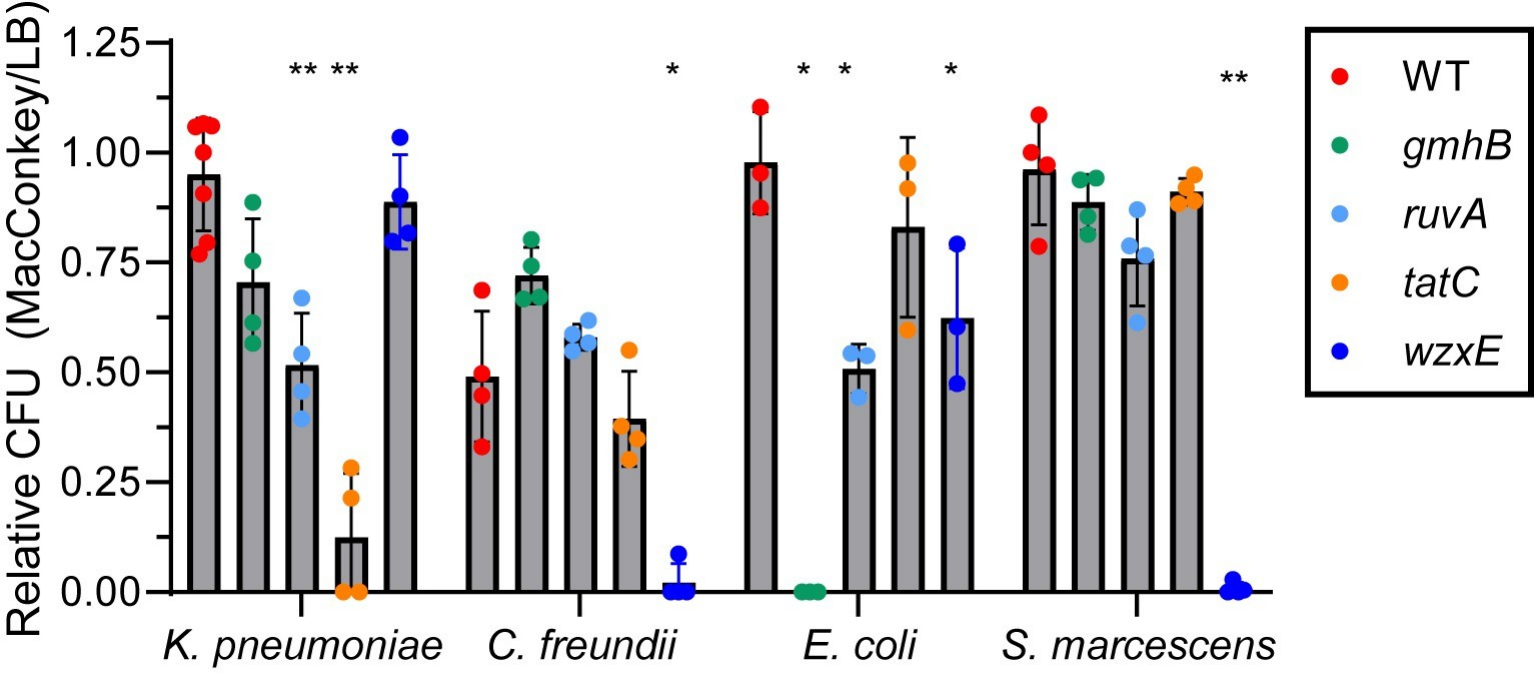
Envelope stress after exposure to bile salts. Bacterial cultures were incubated at 37°C overnight in LB, diluted in PBS to a final concentration of 10^4^ CFU/mL. Bacterial suspensions were spread-plated onto MacConkey agar (Mac) and LB agar (LB) in triplicate and incubated overnight at 27°C Colonies were counted after 48 hours of incubation. Data are presented as the ratio of the number of CFU on MacConkey agar to the number of CFU on LB agar [79]. Significance was determined by paired ANOVA Dunnett’s multiple comparisons test.

We measured the growth of *ruvA*, *tatC*, *gmhB*, and *wzxE* mutants and their respective wild-type strains in four of the five species (*C*. *freundii*, *E*. *coli*, *S*. *marcescens*, and *K*. *pneumoniae*) on MacConkey agar, a medium containing bile salts, and compared survival to growth on Luria agar by CFU enumeration (**Fig 9**). Except for *C*. *freundii*, wild-type strains were resistant to bile salts, which may be encountered in the liver and gastrointestinal tract during infection. Mutation of *gmhB* resulted in a profound sensitivity to bile salts in *E*. *coli*. *ruvA* mutants of *E*. *coli* and *K*. *pneumoniae* were both intermediately sensitive. For *tatC* mutants, *K*. *pneumoniae* was most sensitive and for *wzxE*, both *C*. *freundii* and *S*. *marcescens* were nearly completely susceptible, with scarce CFUs encountered on MacConkey agar. It should be noted that MacConkey agar, in addition to bile salts, also contains crystal violet, which could also be inhibitory to these mutants.

*, *p* < .05; **, *p* < .01.

#### *xi*. Phosphate transport

**Fig 10:**
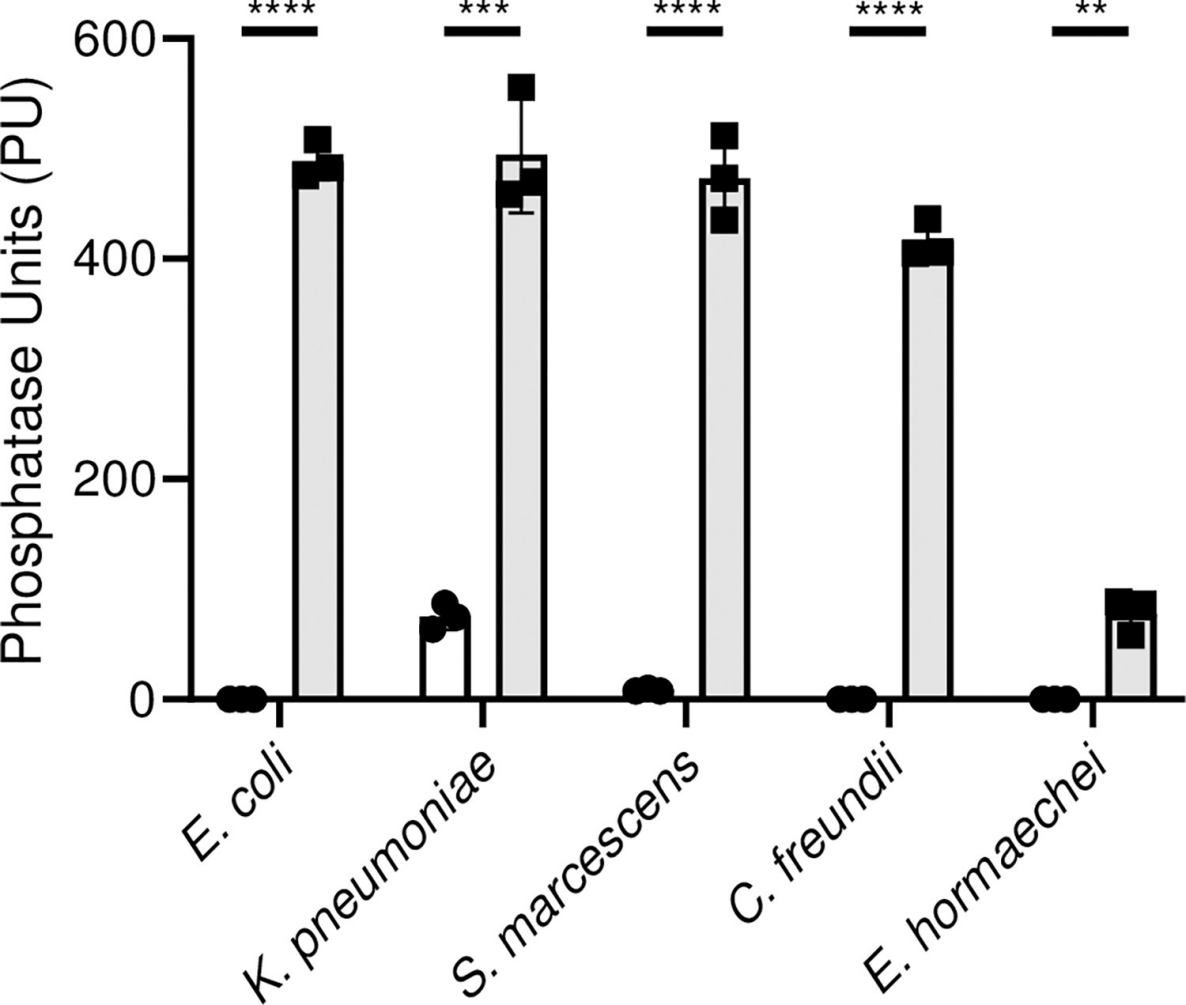
Alkaline phosphatase activity of phosphate transport mutants. Suspensions of wild-type strains normalized to OD_600_ = 0.1 (circle) *E*. *coli* CFT073, *K*. *pneumoniae* Kppr1, *S*. *marcescens* UMH9, *C*. *freundii* UMH14, *E*. *hormaechei* UM_CRE_14 and their respective *pstSCABphoU* mutants (square) were incubated with *p*-nitrophenylphosphate at 37°C for one hour. Hydrolysis of substrate was followed OD_405_ as a measure of alkaline phosphatase activity. Statistical differences were measured with an unpaired *t*-test and (**p<0.01, ***p<0.001, ****I<0.0001).

Mutation of the primary high affinity phosphate ABC transport system (*pstSCABphoU*) results in limitation of phosphate, a requisite nutrient [40]. When bacteria experience phosphate starvation, they upregulate alkaline phosphatase encoded by *phoA*, which scavenges phosphate from monoesters of phosphoric acid [41]. Thus, *pst* mutants for all species and respective wild-type strains were assayed for alkaline phosphatase activity following culture in phosphate-limiting minimal medium [42]. In sharp contrast to their wild-type strains, *pstSCABphoU* mutants of *E*. *coli* CFT073, *K*. *pneumoniae* KPPR1, *C*. *freundii* UMH14, *S*. *marcescens* UMH9 but somewhat less so for *E*. *hormaechei* UM_CRE_14, displayed significantly higher alkaline phosphatase activity when cultured in this phosphate limited condition (**Fig 10**).

#### *xii. In vitro* correlates of infection

Phenotypic tests were conducted on mutants where there had been a previous association of fitness gene and phenotype. While not all assays were conducted on all mutants, when quantifying the statistically significant association of loss of a phenotypic trait with statistically significantly attenuation of the mutant in spleen and liver (**Tables 3** and **4**), a pattern is worth noting. Phenotypes associated with cell envelope integrity of the five species including susceptibility to active human serum, bile salts, and osmotic stress were most frequently observed and included mutants in *prc*, *wzxE*, and *ruvA* (**S2 Table**). Susceptibility of these mutants to these agents correlated with attenuation of virulence and thus may serve as correlates of virulence.

## Discussion

Our previous series of Tn-Seq screens for five Gram-negative bacterial species [10–14] in the Enterobacterales order (**Table 1**), in addition to genomics analysis that permitted identification of a multi-species core genome [17], allowed us to identify and prioritize shared key non-essential fitness factor genes required for maintaining bacterial burden once these bacterial species have gained access to the bloodstream and blood-filtering organs in the murine model of bacteremia (**Table 2**). We constructed mutations in the 18 most highly prioritized genes or operons in four species and 17 mutants in *E*. *hormaechei* for a total of 89 mutants. Each mutant was competed with its respective wild-type parent strain in the bacteremia model by tail vein injection of identical numbers of CFUs of wild-type and mutant bacteria. The same mutants were subjected to a battery of phenotypic assessments. Using these results, we reranked the fitness factor genes based on their respective significant impacts on survival (competitive indices and fold-defects) and number of species attenuated in the murine model of bacteremia (**Tables 3**, **4**, and **5,** and **S3–S5 Figs**).

Using survival in the spleen and secondarily in the liver, 49 of the 89 mutants were significantly outcompeted by their parent strain in the spleen (**Table 3)** and 48 of 89 mutants were significantly outcompeted by their parent strain in the liver (**Table 4**). 63 of 89 mutants were outcompeted by wild-type in either the spleen or liver. Finally, 41 of 89 mutants were outcompeted by wild-type in both the spleen and the liver. After comparing competitive indices determined in the cochallenges and the number of species attenuated by mutation of the genes, several fitness factor genes rose to the top of our consideration. For the spleen, these are: *tatC* (twin arginine transporter), *ruvA* [an endonuclease that resolves Holliday junctions [43]], *xerC* (tyrosine recombinase required for chromosomal segregation at cell division), *gmhB* (D,D-heptose 1,7-bisphosphate phosphatase involved in LPS synthesis), *wzxE* (flippase required for Enterobacterial common antigen synthesis), *prc (*protease involved in peptidoglycan synthesis regulation and inactivation of complement [24,44], and *arcA* (regulator of aerobic respiration). In the liver, a similar hierarchy of fitness factors was authenticated (*tatC*, *gmhB*, and *ruvA)*; however, both *aroC* and *ubiH* (members of the shikimate biosynthesis pathway) were also prominent fitness factor genes for all five species and four species, respectively.

Measurement of *in vitro* growth rates revealed that mutation of fitness factor genes had varying influence on doubling time (**Table 6** and **S4 Fig)**. Indeed, three fourths (65 of 89) of the mutants displayed no significant growth defect during *in vitro* mono-culture in lysogeny broth but their corresponding transposon mutants had nevertheless been outcompeted during the course of experimental bacteremia. These mutants lacked a factor that did not inhibit successful *in vitro* growth but did diminish protection from the host response. On the other hand, some mutants displayed severe growth defects in mono-culture. For example, mutants in the *atp* operon encoding ATP synthase caused a severe *in vitro* growth defect by limiting the synthesis of ATP during both aerobic and anaerobic respiration. As well, *ubiH* and *lpdA* mutants also revealed significant growth defects. In contrast, *wzxE* and *prc* mutants displayed significantly faster doubling times than their wild-type strains in *S*. *marcescens*. Regardless of the *in vitro* growth rate of the mutant, in the original Tn-Seq screens, all 18 genes were identified as bonified hits comparing *in vivo* output (spleen and liver bacterial burden) to *in vitro* input (growth of the inoculum in lysogeny broth). It should be noted that growth in lysogeny broth likely does not recapitulate the host environment since the available nutrients clearly differ between the two milieus. While the three outcomes (no change, slower, or faster growth rates) are interesting, perhaps greater weight should be given to fitness gene mutants that display no significant *in vitro* growth defects but are significantly outcompeted in the mouse model. As well, emphasis could be placed on conserved genes that are not considered metabolic housekeeping genes, based on their predicted or known functions and lack of *in vitro* growth defects. Examples of these include the virulence factors *gmhB* that is important but not essential for LPS synthesis, *wzxE* that encodes a key enzyme (flippase) in ECA synthesis, *tatC* secretion of folded proteins by the twin-arginine transporter, factors providing protection against host defenses including *ruvA*- and *xerC*-mediated genome repair, and *prc* periplasmic protease that regulates peptidoglycan synthesis but also inactivates complement.

While this study is expansive, there are limitations that should be noted. The strategy did not allow us to examine the role of essential genes, which would have not been detected by Tn-Seq. The effects of genes directly upstream or downstream of the identified fitness genes or their operons were also not examined if not identified in the Tn-Seq assessment. Furthermore, as a practical matter, we relied on single type strains of otherwise genetically diverse species and some results may have differed if alternate strains within each species had been used for these studies. Lastly, while not all mutants were complemented, in each of eight cases where complementation was undertaken, the respective phenotype was restored to wild type values.

### Integration of findings into a model of pathogenesis of Gram-negative bacteremia

**Fig 11:**
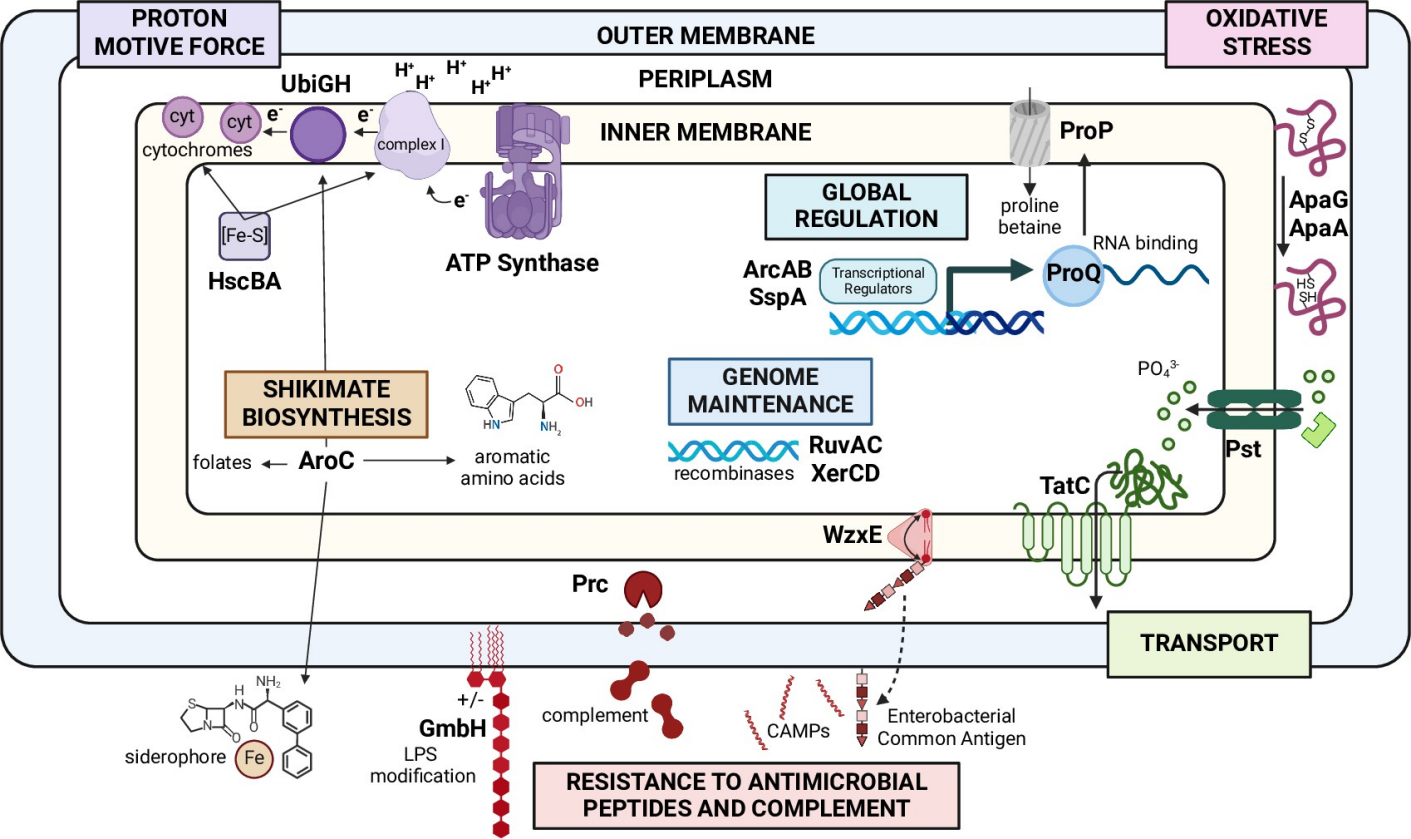
Model of Bacterial Pathogenesis of Bacteremia by Gram-negative Bacteremia. Seven common pathways are depicted that contribute to pathogenesis of bacteremia by five bacterial species within the Enterobacterales. Genes shared within the multi-species core genome of five bacterial species including *E*. *coli*, *K*. *pneumoniae*, *S*. *marcescens*, *C*. *freundii*, and *E*. *hormaechei*, were predicted as common fitness genes using Tn-Seq screens in a murine model of bacteremia. Prioritized mutants were constructed in 18 conserved genes or operons in all 5 species. Mice were cochallenged with each mutant and its respective wild-type strain by tail vein injection of mice. Genes that were validated in from 1–5 species as attenuated as measured by competitive indices are included in the model. Created with BioRender.com.

To integrate the data presented in this report into a comprehensive model of bacteremia pathogenesis for these related species, we considered: 1) the results of Tn-Seq screens for five Enterobacterales species surveyed in the murine model of bacteremia (**Table 1**); 2) the construction of 18 mutants for all four species and 17 mutants for *E*. *hormaechei* in genes predicted as most highly attenuated in the Tn-Seq screens (**Fig 2** and **Table 2**); 3) competitive indices and fold-defects from subsequent cochallenges of wild-type and each mutant in all five species in the murine model of bacteremia (**Tables 3–5** and **S1** and **S2 Figs**); and 4) relevant *in vitro* phenotypes for most of these mutants (**Figs 3–10. Tables 6** and **7**). Taken together, these data allowed us to formulate a working model of the pathogenic mechanisms encoded by key non-essential genes within the multi-species core genome of Gram-negative bacilli during bacteremia (**Fig 11**).

This model of pathogenesis teaches us that specific core pathways shared by Gram-negative species of the Enterobacterales order are critical for infection of the mammalian bloodstream and its blood-filtering organs. First, bacteria must maintain their proton-motive force using ATP synthase to synthesize ATP using reentry of extruded protons from the periplasm into the cytosol. As well, synthesis of iron-sulfur clusters allows the maturation of cytochromes for electron transport. This implies some degree of aerobic respiration is employed by these pathogens in most cases. To that end, global regulator ArcA regulates genes associated with aerobic respiration by sensing available oxygen in conjunction with ArcB to maintain proton motive force. However, in the absence of oxygen, ArcA, with ArcB, can shift metabolism toward fermentation to survive in such an environment, a key representation of the metabolic flexibility displayed by these facultative anaerobes.

Just as critical is the role of the outer envelope to survival in the bloodstream where bacteria must resist the toxic effects of osmotic stress and complement. The protease Prc appears to protect against complement by degrading components of the system. Enterobacterial Common Antigen (ECA) also contributes to defense against complement and antimicrobial peptides. Transport also plays a crucial role by importing phosphate through the Pst ABC transporter. As well, numerous crucial folded proteins are exported across the cytoplasmic membrane by the twin-arginine transport system to maintain viability in the bloodstream. Next, shikimate biosynthesis is required for three key functions that include 1) siderophore synthesis required for iron acquisition that is mandatory for survival in an environment where free iron is tightly sequestered by the host (**Fig 7**); 2) quinone biosynthesis as cofactors in cytochromes for electron transport; and 3) synthesis of aromatic amino acids and folate. This pathway appears to be particularly important for survival in the liver. Bacteria must also resist oxidative stress elicited by innate immune cells including neutrophils and macrophages, which are the first line of host innate defense against infection. However, this was not directly demonstrated by resistance to H_2_O_2_ (**S6 Fig**). Finally, bacteria must maintain the integrity of their genome under oxidative stresses encountered specifically in the host compared to laboratory conditions.

We can also make assertions about the metabolic flexibility of these bacterial pathogens. In studies identifying the most commonly isolated Gram-negative bacilli in cases of bacteremia, at least 80% of the species are classified as facultative anaerobes with a majority being in the Enterobacterales order while *P*. *aeruginosa* and *A*. *baumannii* are strict aerobes dominating the remainder of cases [8,45–47]. Among Gram-negative pathogens, we have shown that the temporal dynamic of bacteremia in the murine model differs by species and anatomical site [9]. Despite having the same “toolbox” of major metabolic pathways at their disposal and evidently utilizing rapid replication to support infection, facultative anaerobes rely on different metabolic processes to various extents in the bacteremia model here. Revisiting why facultative anaerobes cause the largest proportion of Gram-negative bacteremia cases with this perspective poses new questions. A leading hypothesis for their success is their ability to survive in the environment and in the host as a result of metabolic adaptation [48]. However, *A*. *baumannii* and *P*. *aeruginosa* are also environmental pathogens capable of successfully colonizing the bloodstream while remaining strict aerobes [49,50]. Metabolic flexibility may be beneficial in that these species can transition between metabolic pathways during infection, or they may perhaps pivot to use different pathways altogether during infection. These species are possibly better described as being “metabolically advantaged”. This notion would explain why opportunistic bacteria that are facultatively anaerobic are the most common Gram-negative pathogens in cases of bacteremia, yet do not necessarily rely on the same metabolic capabilities as one another. Nevertheless, these bacteria share many common strategies to survive in the mammalian bloodstream as highlighted by the model of pathogenesis.

Prominent examples of fitness factor phenotypes are discussed below in the context of the model of bacteremia pathogenesis where seven key pathways required for the pathogenesis of bacteremia were uncovered and validated (**Fig 11**).

### Maintenance of proton motive force

Mutants in ATP synthase (*atpIBEFHAGDC*) and Iron-sulfur cluster biosynthesis (*hscBA*), required for cytochrome maturation, were validated in *E*. *coli*, *K*. *pneumoniae*, and *S*. *marcescens* in the spleen. Ubiquinone synthesis (*ubiH*), also required for cytochrome maturation and essential for electron transport was validated in *E*. *coli*, *K*. *pneumoniae*, *S*. *marcescens*, and *E*. *hormaechei* in the liver. These data suggest that the levels of aerobic growth, generating ATP via chemiosmosis, may differ in the liver compared to the spleen. While oxygen is tightly bound by hemoglobin within erythrocytes circulating in the bloodstream, we would nevertheless consider arterial blood as a milieu that would favor aerobic growth and thus these genes would support growth in this environment.

### Resistance to complement and osmotic stress

Enterobacterial Common Antigen is a carbohydrate antigen composed of repeating subunits of three amino sugars including *N*-acetylglucosamine, *N*-acetyl D-mannosaminuronic acid, and 4-acetamindo-4,6-dideoxy-D-galactose [51]. This surface structure contributes to resistance to antimicrobial peptides and complement (**Figs 3** and **4**). *wzxE* mutants lacking a flippase, which translocates ECA intermediates from cytosol to periplasm, was attenuated in 4 of 5 species in the spleen and the liver, but not in *C*. *freundii* in either organ. The ECA mutant was successfully complemented for *K*. *pneumoniae* in the human serum killing assay (**S5 Fig**). The antigen is found in three forms including ECA_PG_ that is covalently linked to phospholipids in the outer leaflet of the outer membrane envelope, ECA_LPS_ that is linked to the core polysaccharide of LPS in cases where O-antigen is not synthesized (*i*.*e*., in “rough” strains) and ECA_CYC_ that consists of 4–6 repeating cyclized units (depending on the species) that is localized to the periplasm [51]. ECA structure is conserved in all bacterial species within the Enterobacterales order. Synthesis of ECA may represent a target of therapy as this mutant was susceptible to human serum, osmotic stress, and/or antimicrobial peptides in four species, and bile salts for three species (**Figs 3**, **4**, **8**, and **9** and **S2 Table**). Eight other genes in the *wec* operon encoding ECA were also hit in the Tn-Seq screens (**Table 2**) highlighting the critical importance of this neglected cell surface antigen. Indeed, *wzxE* was the most highly prioritized gene and its operon was also most highly prioritized (**Table 2**).

The *prc* mutant was also validated as attenuated in multiple species and was complemented for restoration to wild type growth in hypotonic medium (**S7 Fig**). The periplasmic protease Prc has at least three roles in pathogenesis including regulation of cell wall synthesis, motility, and complement inactivation [23,24,44]. Prc and its homologues have also been directly demonstrated to be involved in the pathogenesis of Gram-negative bacterial infections. The *prc* mutant of *E*. *coli* RS218 exhibited a decreased ability to cause a high level of bacteremia [24] [as confirmed by us in four species (**Tables 3** and **4**)] and is more susceptible to human serum killing than strain *E*. *coli* RS218 as are the strains in this study (**Fig 3** and **S2 Table**). This sensitivity appears due to the mutant’s decreased ability to avoid the activation of the antibody-dependent and independent classical complement cascades as well as its decreased resistance to killing mediated by the membrane attack complex [24]. Evasion of classical complement-mediated serum killing of our representative Gram-negative bacilli makes Prc a potential target for the development of therapeutic and preventive measures against Gram-negative bacteremia. Indeed, a *prc* mutant is also attenuated in experimental urinary tract infection and has reduced motility due to downregulation of FlhDC, the master regulator of flagellar synthesis [44]. Thus, mutation of *prc* understandably causes multiple deleterious effects on the fitness of Gram-negative bacilli.

In this study, *gmhB* was also a prominent fitness factor gene validated in all five species in the liver and 4 of 5 species in the spleen. Although it does not fit neatly in this category, it is involved in, but not essential for, LPS synthesis. As well, this mutant is susceptible to antimicrobial peptides in *K*. *pneumoniae* KPPR1 [52] and is outcompeted by wild-type during incubation in murine spleen homogenates [16]. GmhB-deficient strains produce a mixed phenotype of full-length and stunted LPS molecules [53]. Nevertheless, some lower molecular weight components of LPS are absent in a *gmhB* mutant [16]. In the bloodstream, bacterial surface structures such as intact LPS are critical to maintain outer membrane permeability and defense against complement and antimicrobial peptides [54]. The *in vivo* spleen defect of this *gmhB* mutant was restored by complementation with *gmhB in trans* [16].

### Transport

The twin arginine translocation system, encoded by *tatABC*, functions in diverse bacterial species to secrete folded proteins across the cytoplasmic membrane. *tatC* mutants were attenuated in all 5 species both in the spleen and in the liver, making this transport system the most impactful shared fitness determinant identified. The *tatC* mutation has been successfully complemented for *C*. *freundii* UMH14 *in vivo* in the bacteremia model (spleen and liver) by expressing *tatC in trans* on a plasmid [14]. In addition, insertional inactivation of *tatC* rendered *K*. *pneumoniae* KPPR1 susceptible to ampicillin *in vitro*; complementation with *tatC in trans* converted the isolate back to intermediate resistance. Likewise, mutation of *tatC* in *E*. *hormaechei* CRE14 displayed sensitivity to piperacillin-tazobactam; complementation with *tatC* returned the isolate to the wild type resistant phenotype [17]. The TatBC complex binds Tat substrate proteins carrying the twin arginine motif on their signal peptides [55] with TatC recognizing the secretion signal sequences for proteins that are exported through this pathway [56]. TatA is recruited to the activated TatBC complex and mediates transport of the substrate. We have now demonstrated that *S*. *marcescens* SufI subcellular localization is TatC-dependent (**Fig 5**), consistent with well-established observations in *E*. *coli* and other species.

Phosphate, a critical nutrient, is imported by an ABC transporter encoded by the *pstSCABphoU* operon. It is well known that *pst* mutants are attenuated in other species [*Proteus mirabilis* in a biofilm and UTI model [42,57] and *Campylobacter jejuni* in the murine gut model of infection [58]]. Thus, we attempted to validate this finding for bacteremia by testing *pstSCABphoU* mutants of all five species in the murine model of bacteremia and found that two, *E*. *coli* CFT073 and *E*. *hormaechei* UM_CRE_14, were attenuated (**Tables 3 and 4**).

### Genome maintenance

*ruvA* and *xerC* DNA recombinase mutants were both outcompeted in all 5 species by each respective wild-type strain in the spleen following cochallenge by tail vein injection (**Table 3**). This is notable, because chromosomal DNA in bacterial cells is subjected to constant attack from exposure to physical and chemical agents (*e*.*g*., reactive oxygen species generated by neutrophils). Bacterial cells have developed systems to repair these defects, preventing cell death or mutation. RuvABC enzyme complexes are involved in DNA recombination and repair and facilitate Holliday junction branch migration and resolution [59,60]. RuvABC repairs damage by catalyzing homologous exchanges between damaged and undamaged DNA [61]. The protein complex resolves Holliday junction intermediates produced by RecA. While the *ruvA* fitness defect was confirmed by competition infection against the wild-type strain using independently constructed mutants in the five species, these mutants had no defect in survival following hydrogen peroxide exposure. However, *K*. *pneumoniae* and *E*. *coli* had modest growth defects in bile salts suggesting additional stressors may impinge on the Ruv system. For *C*. *freundii*, transposon insertion in both *ruvA* and *ruvC* by transposon insertion resulted in a >30-fold loss of fitness (**S2 Fig**). It is tempting to speculate that bacterial DNA damage occurring in the host environment, potentially through immune cell-mediated production of reactive oxygen species, but not adequately mimicked by H_2_O_2_, may also contribute to the requirement for these complexes for full virulence.

Similarly, XerC is a site-specific recombinase that resolves multimers of plasmids and also has a role in the segregation of replicated chromosomes at cell division [62]. *xerC* mutants form filaments that appear unable to fully partition DNA. XerC responds to DNA damage mediated by reactive oxygen species produced by neutrophils during infection and is required for induction of the SOS response, with the result that a mutant defective in *xerC* is more susceptible to a range of DNA-damaging antibiotics including ciprofloxacin and immune-mediated killing [63]. *xerC* was validated as a fitness factor in all 5 species in the spleen and 3 of 5 species in the liver (**Tables 3 and 4**). Combined, XerC and RuvA provide examples of conserved fitness factors that if disrupted by a novel inhibitor, could increase the efficacy of an FDA-approved antibiotic and sensitize bacteria to stressors in the host.

### Shikimate biosynthesis

Mutants in genes encoding enzymes of the shikimate pathway, *aroC*, and *ubiH*, were attenuated in multiple species (**Tables 3–5**). The *aroC* gene encodes chorismate synthase, which performs the terminal enzymatic step in the shikimate biosynthesis pathway to produce chorismate, a fundamental precursor for the biosynthesis of folates, aromatic amino acids, and the quinones, ubiquinone and menaquinone [64,65], which contribute to aerobic and anaerobic respiration, respectively. In addition, chorismate is also required for the biosynthesis of catecholate siderophores including enterobactin and salmochelin. In agreement with our finding that mutation of *aroC* attenuates bacterial fitness in a murine host during systemic infection, alleles for five of the seven enzymatic steps in the shikimate biosynthesis pathway, were predicted by Tn-Seq studies to contribute to bacterial fitness following TVI in the same murine model of bacteremia [10,11,14–16]. This is also in agreement with numerous prior studies demonstrating the fundamental role of shikimate biosynthesis to bacterial pathogenesis in diverse models of disease [66–69]. Fitness deficiencies resulting from mutations within the shikimate biosynthesis pathway are likely caused by the compounding effects of impaired folate biosynthesis, aromatic amino acid auxotrophy, inhibition of aerobic and anaerobic respiration, and limited iron-scavenging capacity. The attenuation of *ubiH* mutants for growth during systemic infection illustrates the utility of aerobic respiration for energy production in the host during bacteremia. Ubiquinone plays a vital role in the electron transport chain during aerobic respiration, allowing for the utilization of oxygen as a terminal electron acceptor [70].

### Global regulation

The Arc two-component system family of bacterial transcriptional regulators, composed of sensor kinase ArcB and response regulator ArcA, senses the modulation of oxygen availability for use as an electron receptor [71]. The *arcA* mutant was attenuated in 4 of 5 species in both the spleen and the liver and displays a small colony phenotype (**Fig 6**) that has been noted by others [33,34]. The Arc system, found in facultatively anaerobic bacteria, mediates the switch from utilizing aerobic respiration to fermentation or anaerobic respiration when oxygen is not being consumed or is limited [72]. Indeed, *arcA* was the most attenuated fitness factor gene transposon mutant in our *K*. *pneumoniae* Tn-Seq bacteremia screen [11]. Notably, the original Tn-Seq screens did not predict *arcA* as a fitness gene in *E*. *coli* and this was validated by lack of attenuation in a specifically constructed *arcA* mutant. This suggests that *E*. *coli* is growing aerobically during bacteremia whereas *K*. *pneumoniae*, *S*. *marcescens*, and *C*. *freundii* may be, at some point during infection, undergoing fermentation during bacteremia.

In our recent work [20], *arcA* mutants were also found to exhibit a dysregulated response to changes in oxygen availability, iron limitation, and membrane perturbations, which bacterial cells may experience during infection. The genetic response of the *arcA* mutants to the cationic antimicrobial peptide polymyxin B supported an expanded role for ArcA as an activator in response to membrane damage. ArcA function was also linked to electron transport chain activity based on its response to proton motive force uncoupling by carbonylcyanide-*m*-chlorophenylhydrazone (CCCP).

### Oxidative stress

Bacteria must resist oxidative stress elicited by innate immune cells during infection. Surprisingly, no genes predicted to enhance oxidative stress resistance (*arcA*, *ruvA*, or *xerC*) were required for bacterial survival following *in vitro* exposure to hydrogen peroxide. However, during infection, many genes may work together to enhance oxidative stress resistance. It is likely that each species has multiple mechanisms to combat this stress during infection. For example, SspA in *K*. *pneumoniae* is required for resistance to oxidative stress but was not tested in these studies [11].

### Mutants not attenuated

Of the 18 bacteremia fitness loci explored in this study, only mutation of *sapABCDF* failed to produce a fitness defect in either the liver or spleen for at least one species. This was surprising since four of the five genes in the *sap* operon were hit in the Tn-seq screens, predicting a role in virulence. A *proP* mutant, attenuated in only one species encodes a proline/betaine transporter for osmotic protection. While *proP per se* was not predicted in any of the Tn-Seq experiments to be a fitness factor, its regulator *proQ*, which encodes an RNA-binding protein that plays a role in stabilizing sRNA-mRNA interactions, was identified. Because *proQ* and *prc* are co-transcribed, we elected to explore the contribution of *proP* to bacteremia fitness to avoid potential polar effects on *prc* expression resulting from mutation of *proQ*. While *proP* mutants were not attenuated during cochallenge, we did generate a *proQ* mutant in *C*. *freundii* UMH14 that was not polar on *prc* and found it to be significantly attenuated in the bacteremia model [Log_10_ competitive indices in spleen and liver were -0.169 (p < .05) and -0.502 (p < .05), respectively], supporting the Tn-Seq prediction that *proQ* is indeed a fitness factor.

## Materials and methods

### Ethics statement

Murine infections were performed in accordance with protocols approved by the University of Michigan Institutional Animal Care and Use Committee and were in accordance with Office of Laboratory Animal Welfare guidelines.

### Determination of the multi-species core genome

In preparation for the current analysis described in this report, we used a pan-genome pipeline developed by our group including colleagues at the *J*. *Craig Venter Institute* along with ~15,000 sequenced genomes to identify the multispecies core genome shared by *E*. *coli*, *K*. *pneumoniae*, *C*. *freundii*, and *S*. *marcescens*. Using criteria of genes being present in four of five species and that 70% of strains of each species carried the gene, we predicted a core genome of 2850 genes shared in the multi-species core genome of these four species [17].

### Scoring rubric for ranking and prioritization of fitness mutants

For the fitness genes predicted by Tn-Seq in the murine bacteremia model for each of the four bacterial species listed in **Table 1** (*E*. *hormaechei* was initially excluded because Tn-Seq studies were not completed at that stage of the study), we prioritized each fitness gene for each of the four species using the Tn-Seq data, according to a scoring rubric [17], reiterated here, based on four major additive criteria: 1) the magnitude of the fitness defect associated with a gene in any one species: 3 points for fitness genes in each species’ top 20 genes; 2 points for genes ranked 21–40; and 1 point for each gene ranked 41–60; 2) a gene was a fitness factor in multiple species: 3 points for a fitness gene found in 3 or more species, 2 points if found in 2 species; 3) whether multiple fitness genes reside in the same operon: 3 points if 5–9 other fitness genes are encoded in the same operon, 2 points if 3–4 other fitness genes are encoded in the same operon, and 1 point if 1–2 other fitness factors are encoded in the same operon. An additional 1 point was awarded to fitness genes encoded in operons where >49% of the operon loci were predicted to be fitness factors; 4) mutation of a fitness gene was found to confer increased antibiotic susceptibility to any of the following antibiotics: ciprofloxacin, rifampin, vancomycin, ampicillin, sulfamethoxazole, gentamicin, or metronidazole in *E*. *coli* BW25113 [19]; 2 points for such a fitness gene. The sum of the four criteria above was used to assign individual scores for all fitness genes or found in each species pan-genome core. Operon scores were calculated by summing all the individual scores of fitness genes encode within that operon. Data are presented in **Table 2**.

### Construction of mutants in prioritized fitness genes

Fitness gene mutations in all species were generated by lambda red recombineering using established protocols [13,14,66,73]. For *C*. *freundii*, *E*. *coli*, *K*. *pneumoniae*, and *S*. *marcescens* the *nptII* kanamycin resistance cassette was PCR-amplified from pKD4 [73,74] and directed to in-frame deletions of target genes via 5′-end homologous sequences (**S1 Table**). At least six codons on the 3′-end of each gene were left intact to preserve translatability of downstream genes when target genes were internal to a polycistronic message. Insertion sequences were matched to the transcriptional orientation of the original open reading frame. For *E*. *hormaechei*, the *acc(3)IV* apramycin resistance cassette was amplified from pUC18-miniTn7T-Apr [75] and used in place of kanamycin resistance. Recombination was facilitated by functions encoded on pKD46, pSIM19, or pSIM18 depending on the species mutated [73,74]. All mutations were confirmed by analyzing the sizes of PCR-amplified alleles and antibiotic resistance cassettes. In most cases mutations were further validated by sequencing. Recombineering plasmids were cured prior to phenotypic analysis. For selected mutants, complementation was achieved by either: a) cloning deleted gene sequences into pGEN-MCS (a plasmid stably maintained in these five type strains [76] during experimental infections in the absence of antibiotics) and transforming the resultant plasmid constructs into the respective mutants; or b) by allelic replacement into the chromosome with the wild-type gene.

To demonstrate complementation of *prc* in *Citrobacter freundii* UMH14 (**S7 Fig**), LB cultures (2 mL) supplemented with gentamicin (10 μg/mL) were inoculated from freezer stocks, maintaining the plasmids described below, and cultured for 16 h at 37° C with aeration. Stationary phase cultures were normalized to 1.0 OD_600_ in fresh LB medium and inoculated 1:100 in triplicate wells of a Honeycomb 2 microtiter plate (Bioscreen) containing 300 μL lysogeny broth (1% NaCl) or lysogeny broth lacking NaCl (0% NaCl). The microtiter plate was incubated at 37°C with continuous shaking and culture densities were measured every 15 m for 10 h at OD_600_ in a Bioscreen C growth curve analyzer. The *prc* complementation vector (pPrc) was generated using pBBR1MCS-5 (Vector) [77] linearized by a double restriction digest with an orientation of 5’ BamHI-HF (NEB) and 3’ EcoRI-HF (NEB). First, a vector containing the presumed native promoter (592 NT’s 5’ of *proQ*) and both the *proQ* and *prc* ORFs was cloned using NEBuilder HiFi DNA Assembly Master Mix (NEB) in a two-piece assembly of the digested pBBR1-mcs5 vector and a PCR product containing nucleotides 2,081,680–2,078,143 amplified using Q5 High-Fidelity DNA Polymerase (NEB) from a *C*. *freundii* UMH14 genomic DNA template. In order to eliminate the contributions of the ProQ while maintaining the genetic context of *prc* and its promoter a nonsense mutation was incorporated into the start codon *proQ* using the SPRINP method of site-directed mutagenesis [78] with Q5 High-Fidelity DNA Polymerases (NEB). The pBBR1MCS-5 and pPrc plasmids were mobilized into wildtype *C*. *freundii* UMH14 and the *prc* mutant using electrotransformation. Primers for pPrc construction are listed below:

**Table:** 

Primer name / description	NT sequence
comp vector native proQ prc Fw	CGGCCGCTCTAGAACTAGTGAACAAAATAGAGCTATACACG
comp vector native proQ prc Rv	TATCGATAAGCTTGATATCGAATTTTCTTTTAACCTCAATTTAACAAAAC
SDM proQ start to stop Fw	TACGTCCGTTGTAATCAGGAAATTTCTAAGAAAATCAACCTAAGTT
SDM proQ start to stop Rv	AACTTAGGTTGATTTTCTTAGAAATTTCCTGATTACAACGGACGTA

### Bacterial growth rates

Bacterial strains were cultured in LB medium [79] with aeration and optical density (600 nm) was measured in 10–15 min intervals using either a LogPhase 600 (Agilent) or BioScreen C (Growth Curves USA) automated growth curve system. Maximal specific growth rates and doubling times were calculated using AMiGA software [21]. Relative growth rates of each mutant were calculated for three biological replicates in comparison to a wild-type control cultured on the same microtiter plate. In addition, competition experiments were undertaken in lysogeny broth with six selected mutants versus their respective wildtype strains. Data were expressed as competitive indices.

### Murine model of bacteremia

Murine infections were performed in accordance with protocols approved by the University of Michigan Institutional Animal Care and Use Committee and were in accordance with Office of Laboratory Animal Welfare guidelines. Bacteria for murine infection experiments were prepared by subculturing overnight LB growth into fresh medium and incubating for 2.5 hours. Exponential phase bacteria were collected by centrifugation and resuspended in an appropriate volume of PBS. Female 6–8-weeks old C57BL/6 mice were infected with bacterial suspension (containing the indicated number of cfu) of *E*. *coli* CFT073 (1 x 10^7^), *K*. *pneumoniae* KPPR1 (1 x 10^5^), S. *marcescens* UMH9 (5 x 10^6^), *C*. *freundii* UMH14 (1 x 10^8^), and *E*. *hormaechei* UM_CRE_14 (1 x 10^8^) via tail vein injection [80], unless otherwise noted. Total inocula for the five species were based on previous trials that 1) allowed for no bottleneck in the original trials (*i*.*e*., no stochastic loss of mutants during the tail vein challenge); 2) accounted for the size and complexity of each transposon pool; and 3) avoided lethality following injection (see footnote ^a^ to Tables 3 and 4 for number of cfu in each inoculum). The spleen and liver from mice sacrificed at 24 hours post-inoculation were homogenized in PBS and ten-fold serial dilutions were plated on LB agar to determine the bacterial burden. For *S*. *marcescens*, kidneys were also used to determine bacterial burden. For competition infections, the wild-type *strain* was mixed with antibiotic-resistant mutant constructs at a 1:1 ratio prior to inoculation. The viable count for each strain was determined for both the inoculum (input) and organ homogenates (output) by serial dilution and differential plating on LB and LB containing antibiotics. The competitive index (CI) was calculated as follows: (CFU_mutant_/CFU_wild-type_)^output^/(CFU_mutant_/CFU_wild-type_)^input^. All murine infections were conducted using protocols approved by the University of Michigan Institutional Animal Care and Use Committee and in accordance with the Office of Laboratory Animal Welfare guidelines.

### Susceptibility to ciprofloxacin

Susceptibility to ciprofloxacin was measured by disk diffusion, which was performed for wild-type strains and their respective *xerC* and *ruvA* mutants. LB was inoculated and cultured overnight at 37°C. Bacterial suspensions were normalized to an OD_600_ of 0.1 in accordance with the McFarland Standard Protocol [81]. Sterile swabs were used to create a bacterial lawn on Mueller-Hinton agar plates. Ciprofloxacin disks (0.5 μg) were placed in the center of each agar plate using sterile forceps. Following incubation for 18–24 hours at 37°C, the diameter of the zone of inhibition was measured according to Kirby-Bauer Disk Diffusion Susceptibility Test Protocol [82].

### Susceptibility to antimicrobial peptides

CFU/ml and relative survival as compared to the wild-type strain survival was determined by incubating 10^7^ CFU/mL log-phase bacterial cells with polymyxin B in PBS, pH 7.4 for 45 min at 37°C and then plating for viable counts on Luria agar [83,84]. See legend to Fig 3 for polymyxin B concentrations for each species.

### Susceptibility to human serum

Susceptibility to bactericidal activity of human serum was measured by incubating 10^7^ CFU/mL log-phase bacteria in 90% pooled human serum for *K*. *pneumoniae* KPPR1 and 40% pooled human serum for the other four species. Viability was measured by plating samples on Luria agar and determining CFUs after incubation times of 0 and 90 minutes. Both active serum and heat-inactivated (56°C, 60 min) serum were tested [13,16,85,86].

### Siderophore production

Siderophore production was detected on chrome azurol S (CAS) plates supplemented with tryptone. Samples of overnight stationary phase cultures (2 μl) were spotted onto CAS agar plates and incubated at 37°C for 16 h. Siderophore activity was indicated by a shift from blue to yellow color within and surrounding the colonies. Gray scale image, which facilitate the detection of the blue (dark pixelation) to yellow (light pixelation) coloration in the CAS agar, was captured on a BioRad Gel Doc system with a “Coomassie Blue” setting and a white acrylic filter. Halo diameters were measured by ImageJ software as described in the legend to **Fig 7**. Media were prepared as described previously [87] with the sole modification of replacing 0.1% casamino acids with 1% tryptone to supplement the aromatic amino acid auxotroph inherent to mutation of *aroC*.

### Osmotic stress

Susceptibility to osmotic stress was determined by incubating 10^7^ CFU/ml log-phase bacteria in PBS, pH7.4 with or without 2M D-sorbitol for 30 min at 37°C and then plating for viable counts [86,88].

### Oxidative stress assessed by exposure to H_2_O_2_

To determine gene contributions to oxidative stress resistance, overnight bacterial cultures were adjusted to 1x10^7^ CFU/mL in PBS containing 1mM hydrogen peroxide. Each strain was incubated for 2 hours at 37°C, and quantitative culture was used to define the abundance of each strain at the input (t = 0) and output (t = 2) incubation. Percent survival was defined as [(CFU at t = 2)/(CFU at t = 0)] x 100. Fold change was defined as (wild-type %Survival/mutant %Survival) [11].

### Envelope stress

Envelope stress sensitivity was measured by growth on MacConkey agar, a medium containing bile salts [79]. Mutants in *ruvA*, *tatC*, *gmhB*, and *wzxE* mutants and their respective wildtype strains in *E*. *coli*, *K*. *pneumoniae*, *C*. *freundii*, *S*. *marcescens*, were evaluated. Cultures were incubated at 37°C overnight, then diluted in PBS to a final concentration of 10^4^ CFU/mL. Diluted cultures were then spread plated on MacConkey agar and LB agar in triplicate and incubated overnight at 27°C. Colonies were counted after 48 hours of incubation. The CFU on MacConkey agar was divided by the CFU on LB agar on each day and compared to the ratio of their respective WT for each day.

### Phosphate import

Phosphate transport mutants constitutively express alkaline phosphatase, whereas wild-type strains do not. Bacterial strains were cultured in phosphate-limiting minimal medium. Bacterial suspensions were normalized to OD_600_ = 0.1. Enzyme activity was measured by following hydrolysis of 0.4% *p*-nitrophenylphosphate at OD_405_ following incubation at 37°C for 1 hr as described [42,79,89].

### Twin arginine protein export

To generate the *S*. *marcescens* SufI signal peptide-GFP translational fusion, the sequence encoding amino acid residues 1 to 35 of the N-terminal end of BVG96_RS17270 [90] was PCR amplified using Q5 polymerase (NEB) and cloned via NEBuilder HiFi DNA Assembly (NEB) into plasmid pIDMv5K-J231000-Dasher-GFP-B1006, previously modified with the replacement of the kanamycin resistance gene with a gene encoding gentamycin resistance. The resultant plasmid was confirmed by sequencing and transformed into *S*. *marcescens* UMH9 and Δ*tatC*::*nptII* via electroporation. Bacteria harboring the SufI-GFP fusion or vector control plasmid were cultured to mid exponential growth phase then fixed with 4% paraformaldehyde for 20 min at 25°C, washed with an equal volume of PBS, and normalized to 1x10^9^ CFU. Bacteria were mounted with Vectashield anti-fade medium (Vector Laboratories, Inc), and observed with a Nikon Ti2 Widefield microscope with a 100X oil immersion lens. Images were captured with an ORCA-Fusion Digital CMPS camera (Hamamatsu) and analyzed with ImageJ (Version 1.54f) to quantify cell length and fluorescence intensity (n>100).

### Statistical analysis

Tests used for statistical analysis are noted in the respective footnotes to tables and figure legends. Specifically, to control for statistical significance when multiple comparisons were analyzed, Prism software (GraphPad) was used adjust calculated P values using False Discovery Rate analysis.

## Supporting information

S1 Fig Competitive Indices ± Standard Deviations in Spleen and Liver from murine tail vein cochallenges. Fitness gene mutants were competed with wild-type bacteria in a TVI murine bacteremia model. Mice were sacrificed and bacteria were enumerated by CFU from spleen and liver homogenates 24 h after inoculation (Tables 3 and 4). Bars represent the mean of log-transformed competitive indices ± standard deviation. False discovery rates were calculated for each species independently and q values of <0.05 are indicated by an asterisk. Abbreviations: Cf, *C*. *freundii*; Eh, *E*. *hormaechei*; Ec, *E*. *coli*; Kp, *K*. *pneumoniae*; Sm, *S*. *marcescens*; ND, not determined.
(PDF)

S2 Fig Fold-defects of fitness gene mutants as compared to wild-type strains in the murine model of bacteremia. Competitive indices in Tables 3 and 4 have been converted to fold-defects, averaged for all species, and depicted separately for liver and spleen. Bars represent standard deviations.
(PDF)

S3 Fig Competitive Indices ± Standard Deviations for S. marcescens UMH9 in the kidney following murine tail vein injection cochallenges. Fitness gene mutants were competed with wild-type S. marcescens in a TVI murine bacteremia model. Mice were sacrificed and bacteria were enumerated by CFU from kidney homogenates 24 h after inoculation (Table 5). Bars represent the mean of log-transformed competitive indices ± standard deviation. False discovery rates resulting in q values of <0.05 are indicated by an asterisk.
(PDF)

S4 Fig Growth characteristics of bacteremia fitness mutants. A-E. Relative growth rates (mutant/wild-type) and doubling times were calculated from the maximal specific growth rate in exponential phase for each mutant and wild-type strain. The statistical significance of relative growth rates was assessed by one-way ANOVA with Dunnett’s multiple comparisons test against the hypothetical value of 1 representing wild-type. Adjusted P values are indicated by asterisks: *, p<0.05; **, *p*<0.01; ***, *p*<0.001; ****, *p*<0.0001. Bars represent the means from three biological replicates ± the standard deviation. Wild-type controls from independent experimental plates were included for each species and set of mutants, as shown in doubling time calculations.
(PDF)

S5 Fig Complementation of C. freundii UMH14 prc mutant and K. pneumoniae KPPR1 wzxE (Enterobacterial Common Antigen) mutant. In the Osmotic Stress Assay, (A) 1x10^7^ CFU/mL C. freundii UMH14 wild type strain, its prc mutant, and its complemented mutant and (B) K. pneumoniae KPPR1 wild type strain, its wzxE mutant, and its complemented mutant were incubated with 0M or 2M D-sorbitol in PBS to induce osmotic stress for 30 min. Individual CFUs were determined after 30-minute incubation. Bacterial viability was calculated relative to 0M sorbitol. In the Serum Resistance Assay (C), 1x10^7^ CFU/mL of K. pneumoniae KPPR1 wild type strain, its wzxE mutant, and its complemented mutant were incubated for 90 min at 37°C with heat-inactivated human serum (left) or 90% pooled human serum (right) to assay for complement-mediated killing. Viability at t = 90 min was calculated relative to t = 0 min with statistical differences in susceptibility to heat-inactivated human serum or normal human serum. Data are presented as the mean + SEM and are representative of 3 independent experiments each with 3 biological replicates. Statistical significance was assessed by an unpaired t-test (*p<0.0001).
(PDF)

S6 Fig Oxidative stress by exposure to H_2_O_2_. Bacterial strains with mutations in the genes arcA, ruvA, and xerC in each species of interest were exposed to hydrogen peroxide to measure resistance to oxidative stress. Percent survival was calculated by comparing bacterial survival at 2 hours to the input. Fold change was calculated by dividing percent survival of each mutant to its respective wild-type strain. No mutant conveyed statistically significant resistance to hydrogen peroxide stress as assessed by a one-sample t-test with a hypothetical value of 1, representing survival of the wild-type strain. Data are means of 3 independent experiments.
(PDF)

S7 Fig Complementation of a *C*. *freundii* UMH14 *prc* dependent growth defect in a hypotonic medium. C. freundii UMH14 wild-type and prc::nptII grown aerobically in either LB medium (1% NaCl LB) or a hypotonic LB medium (0% NaCl LB) maintaining either the pBBR1MCS-5 empty vector plasmid (Vector) or a pBBR1MCS-5 derivative encoding the prc locus under control of its native promoter (pprc). Representative growth curves are the mean and standard deviation (small and obscured by symbols) of technical triplicate wells derived from OD600 measurements taken every 15 m for 10 h in a plate reader.
(PDF)

S1 Table Primer sequences used for construction of prioritized fitness gene mutants. (PDF)

S2 Table Statistically significant *In vitro* phenotypes of fitness gene mutants. (PDF)
