# Supplementary material for: Fitness factor genes conserved within the multi-species core genome of Gram-negative Enterobacterales species contribute to bacteremia pathogenesis
Source: PLoS Pathog. 2024 Aug 23;20(8):e1012495. doi: 10.1371/journal.ppat.1012495 (PMC11376589; doi:10.1371/journal.ppat.1012495)
Supplement: S1 Fig — Fitness gene mutants were competed with wild-type bacteria in a TVI murine bacteremia model. Mice were sacrificed and bacteria were enumerated by CFU from spleen and liver homogenates 24 h after inoculation (Tables 3 and 4). Bars represent the mean of log-transformed competitive indices ± standard deviation. False discovery rates were calculated for each species independently and q values of <0.05 are indicated by an asterisk. Abbreviations: Cf, C. freundii; Eh, E. hormaechei; Ec, E. coli; Kp, K. pneumoniae; Sm, S. marcescens; ND, not determined. (PDF) [file ppat.1012495.s001.pdf]

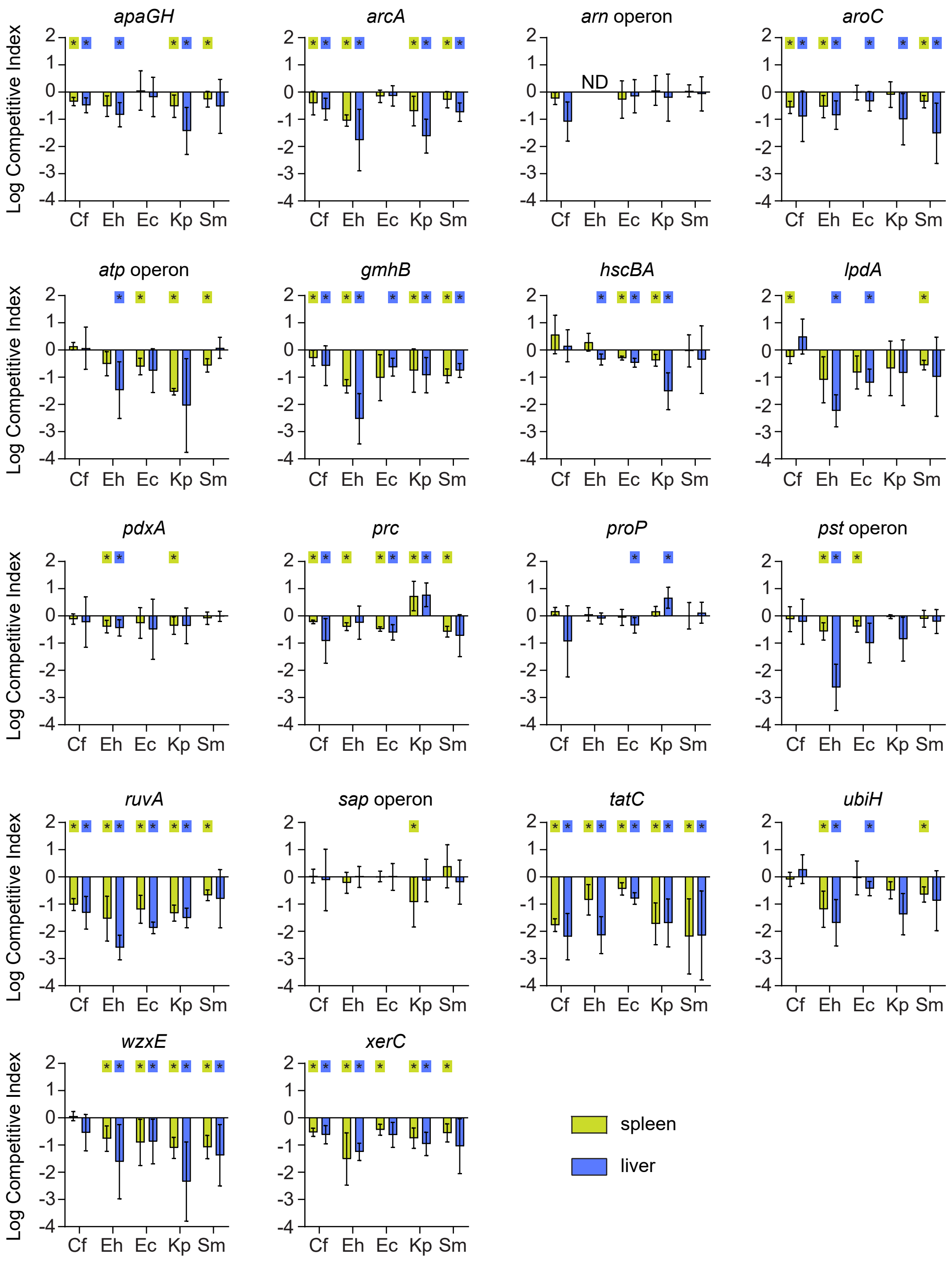

Supplemental Fig 1. Competitive Indices ± Standard Deviations in Spleen and Liver from murine tail vein cochallenges. Fitness gene mutants were competed with wild-type bacteria in a TVI murine bacteremia model. Mice were sacrificed and bacteria were enumerated by CFU from spleen and liver homogenates 24 h after inoculation (Table 3 and 4). Bars represent the mean of log-transformed competitive indices ± standard deviation. False discovery rates were calculated for each species independently and q values of <0.05 are indicated by an asterisk. Abbreviations: Cf, *C. freundii*; Eh, *E. hormaechei*; Ec, *E. coli*; Kp, *K. pneumoniae*; Sm, *S. marcescens*; ND, not determined.
