# Supplementary material for: Fitness factor genes conserved within the multi-species core genome of Gram-negative Enterobacterales species contribute to bacteremia pathogenesis
Source: PLoS Pathog. 2024 Aug 23;20(8):e1012495. doi: 10.1371/journal.ppat.1012495 (PMC11376589; doi:10.1371/journal.ppat.1012495)
Supplement: S2 Fig — Competitive indices in Tables 3 and 4 have been converted to fold-defects, averaged for all species, and depicted separately for liver and spleen. Bars represent standard deviations. (PDF) [file ppat.1012495.s002.pdf]

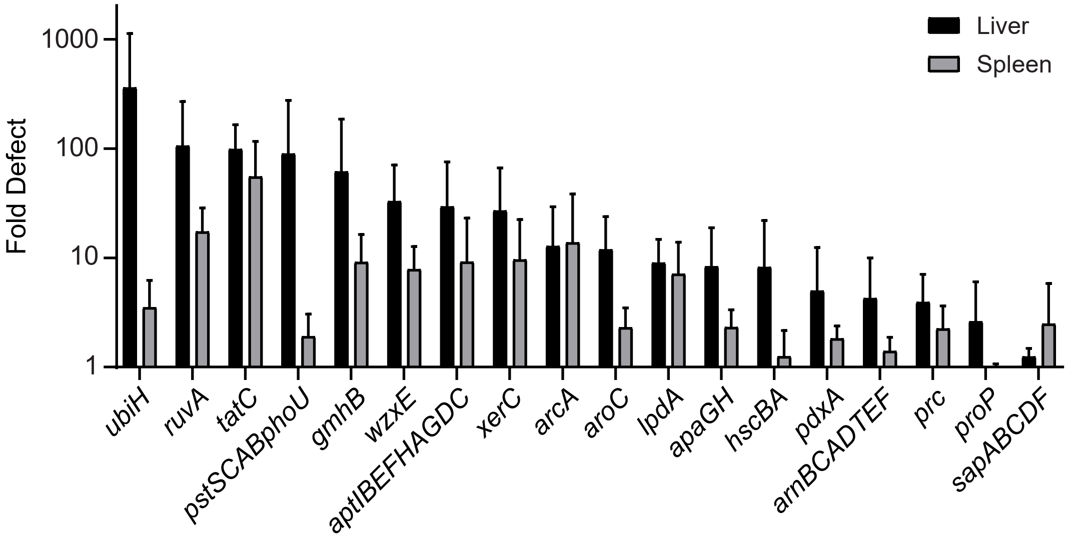

Supplemental Fig 2. Fold-defects of fitness gene mutants as compared to wild-type strains in the murine model of bacteremia. Competitive indices in Table 3 and Table 4 have been converted to fold-defects, averaged for all species, and depicted separately for liver and spleen. Bars represent standard deviations.
