# Supplementary material for: Fitness factor genes conserved within the multi-species core genome of Gram-negative Enterobacterales species contribute to bacteremia pathogenesis
Source: PLoS Pathog. 2024 Aug 23;20(8):e1012495. doi: 10.1371/journal.ppat.1012495 (PMC11376589; doi:10.1371/journal.ppat.1012495)
Supplement: S3 Fig — Fitness gene mutants were competed with wild-type S. marcescens in a TVI murine bacteremia model. Mice were sacrificed and bacteria were enumerated by CFU from kidney homogenates 24 h after inoculation (Table 5). Bars represent the mean of log-transformed competitive indices ± standard deviation. False discovery rates resulting in q values of <0.05 are indicated by an asterisk. (PDF) [file ppat.1012495.s003.pdf]

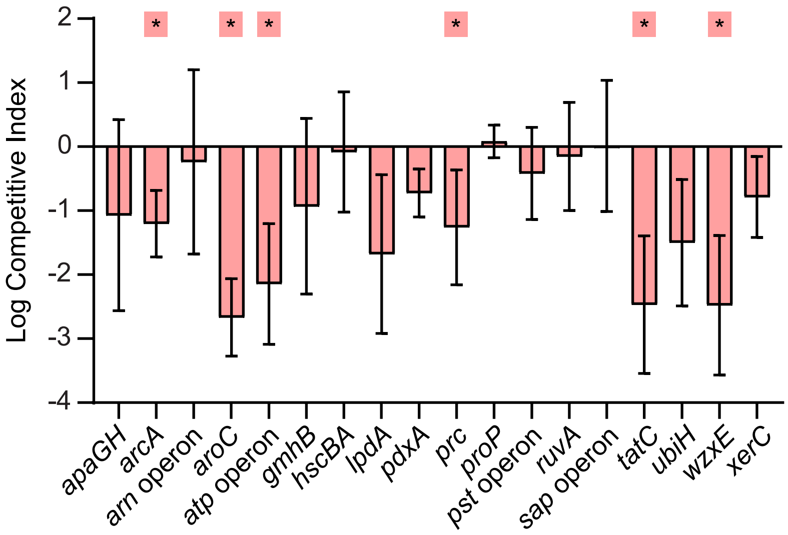

Supplementary Fig 3. Competitive Indices ± Standard Deviations for *S. marcescens* UMH9 in the kidney following murine tail vein injection cochallenges. Fitness gene mutants were competed with wild-type *S. marcescens* in a TVI murine bacteremia model. Mice were sacrificed and bacteria were enumerated by CFU from kidney homogenates 24 h after inoculation (Table 5). Bars represent the mean of log-transformed competitive indices ± standard deviation. False discovery rates resulting in q values of <0.05 are indicated by an asterisk.
