# Supplementary material for: Fitness factor genes conserved within the multi-species core genome of Gram-negative Enterobacterales species contribute to bacteremia pathogenesis
Source: PLoS Pathog. 2024 Aug 23;20(8):e1012495. doi: 10.1371/journal.ppat.1012495 (PMC11376589; doi:10.1371/journal.ppat.1012495)
Supplement: S4 Fig — A-E. Relative growth rates (mutant/wild-type) and doubling times were calculated from the maximal specific growth rate in exponential phase for each mutant and wild-type strain. The statistical significance of relative growth rates was assessed by one-way ANOVA with Dunnett’s multiple comparisons test against the hypothetical value of 1 representing wild-type. Adjusted P values are indicated by asterisks: *, p<0.05; **, p<0.01; ***, p<0.001; ****, p<0.0001. Bars represent the means from three biological replicates ± the standard deviation. Wild-type controls from independent experimental plates were included for each species and set of mutants, as shown in doubling time calculations. (PDF) [file ppat.1012495.s004.pdf]

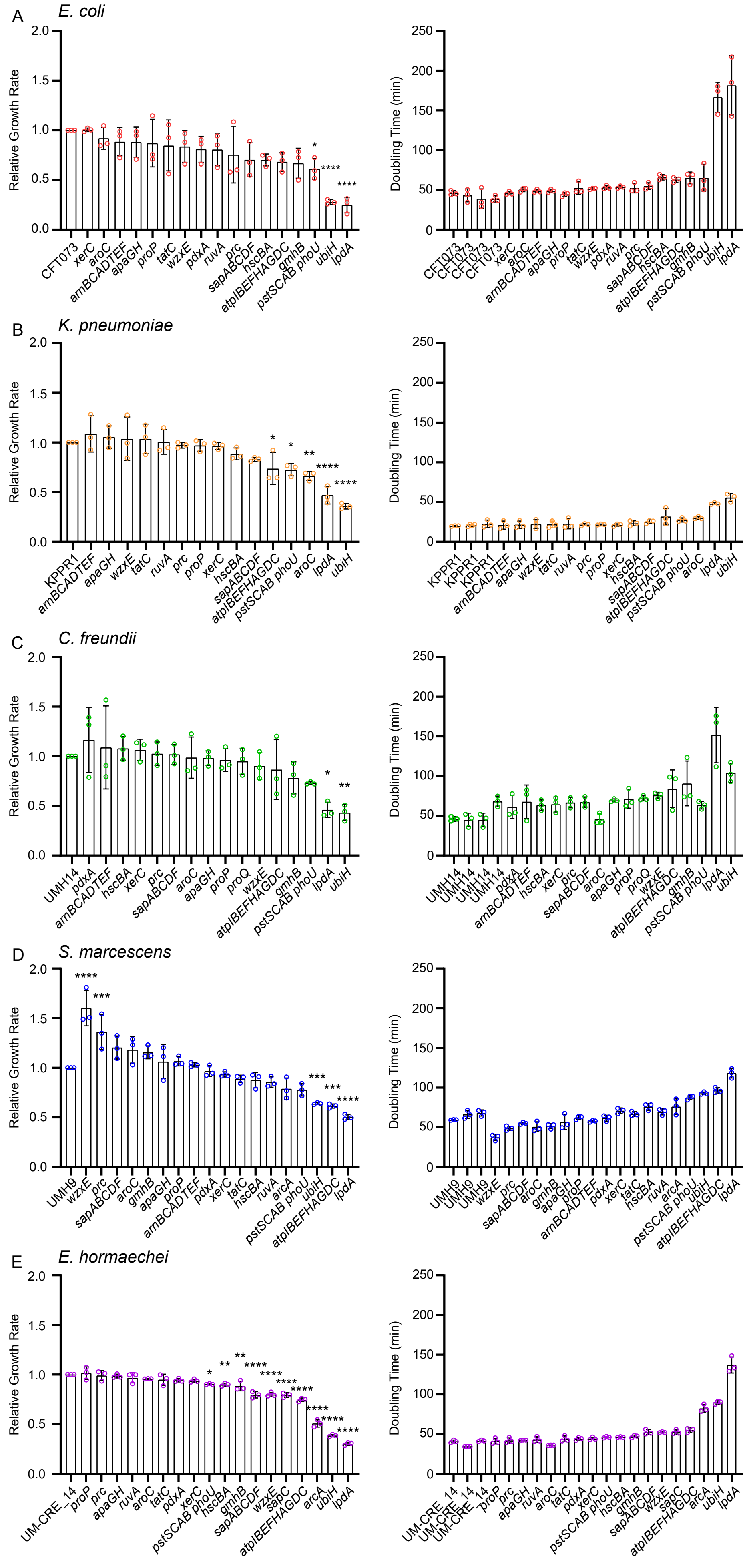
A
E. coli
Relative Growth Rate
Doubling Time (min)
CFT073
xerC
aroC
arnBCADTEF
apaGH
proP
tatC
wzxE
pdxA
ruvA
prc
sapABCDF
hscBA
atpIBEFHAGDC
gmhB
pstSCAB phoU
ubiH
lpdA
B
K. pneumoniae
Relative Growth Rate
Doubling Time (min)
KPPR1
arnBCADTEF
apaGH
wzxE
tatC
ruvA
prc
proP
xerC
hscBA
sapABCDF
atpIBEFHAGDC
pstSCAB phoU
aroC
lpdA
ubiH
C
C. freundii
Relative Growth Rate
Doubling Time (min)
UMH14
pdxA
arnBCADTEF
hscBA
xerC
prc
sapABCDF
aroC
apaGH
proP
proQ
wzxE
atpIBEFHAGDC
gmhB
pstSCAB phoU
lpdA
ubiH
D
S. marcescens
Relative Growth Rate
Doubling Time (min)
UMH9
wzxE
prc
sapABCDF
aroC
gmhB
apaGH
proP
arnBCADTEF
pdxA
xerC
tatC
hscBA
ruvA
arcA
pstSCAB phoU
ubiH
atpIBEFHAGDC
lpdA
E
E. hormaechei
Relative Growth Rate
Doubling Time (min)
UM-CRE_14
proP
prc
apaGH
ruvA
aroC
tatC
pdxA
xerC
pstSCAB phoU
hscBA
gmhB
sapABCDF
wzxE
sapC
atpIBEFHAGDC
arcA
ubiH
lpdA

Supplemental Fig. 4. Growth characteristics of bacteremia fitness mutants. A-E. Relative growth rates (mutant/wild-type) and doubling times were calculated from the maximal specific growth rate in exponential phase for each mutant and wild-type strain. The statistical significance of relative growth rates was assessed by one-way ANOVA with Dunnett's multiple comparisons test against the hypothetical value of 1 representing wild-type. Adjusted P values are indicated by asterisks: *, $p<0.05$; **, $p<0.01$; ***, $p<0.001$; ****, $p<0.0001$. Bars represent the means from three biological replicates ± the standard deviation. Wild-type controls from independent experimental plates were included for each species and set of mutants, as shown in doubling time calculations.
