# Supplementary material for: Fitness factor genes conserved within the multi-species core genome of Gram-negative Enterobacterales species contribute to bacteremia pathogenesis
Source: PLoS Pathog. 2024 Aug 23;20(8):e1012495. doi: 10.1371/journal.ppat.1012495 (PMC11376589; doi:10.1371/journal.ppat.1012495)
Supplement: S5 Fig — In the Osmotic Stress Assay, (A) 1x10^7^ CFU/mL C. freundii UMH14 wild type strain, its prc mutant, and its complemented mutant and (B) K. pneumoniae KPPR1 wild type strain, its wzxE mutant, and its complemented mutant were incubated with 0M or 2M D-sorbitol in PBS to induce osmotic stress for 30 min. Individual CFUs were determined after 30-minute incubation. Bacterial viability was calculated relative to 0M sorbitol. In the Serum Resistance Assay (C), 1x10^7^ CFU/mL of K. pneumoniae KPPR1 wild type strain, its wzxE mutant, and its complemented mutant were incubated for 90 min at 37°C with heat-inactivated human serum (left) or 90% pooled human serum (right) to assay for complement-mediated killing. Viability at t = 90 min was calculated relative to t = 0 min with statistical differences in susceptibility to heat-inactivated human serum or normal human serum. Data are presented as the mean + SEM and are representative of 3 independent experiments each with 3 biological replicates. Statistical significance was assessed by an unpaired t-test (*p<0.0001). (PDF) [file ppat.1012495.s005.pdf]

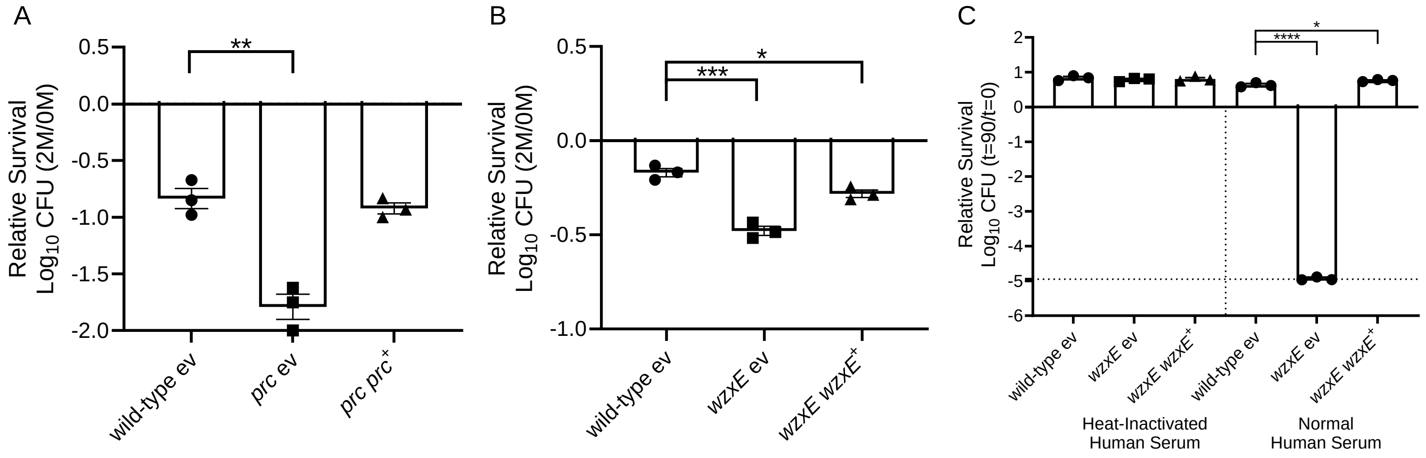

Supplementary Fig 5. Complementation of *C. freundii* UMH14 *prc* mutant and *K. pneumoniae* KPPR1 *wzxE* (Enterobacterial Common Antigen) mutant. In the Osmotic Stress Assay, **(A)** 1x10^7 CFU/mL *C. freundii* UMH14 wild type strain, its *prc* mutant, and its complemented mutant and (**B**) *K. pneumoniae* KPPR1 wild type strain, its *wzxE* mutant, and its complemented mutant were incubated with 0M or 2M D-sorbitol in PBS to induce osmotic stress for 30 min. Individual CFUs were determined after 30-minute incubation. Bacterial viability was calculated relative to 0M sorbitol. In the Serum Resistance Assay **(C)**, 1x10^7 CFU/mL of *K. pneumoniae* KPPR1 wild type strain, its *wzxE* mutant, and its complemented mutant were incubated for 90 min at 37°C with heat-inactivated human serum (left) or 90% pooled human serum (right) to assay for complement-mediated killing. Viability at t=90 min was calculated relative to t=0 min with statistical differences in susceptibility to heat-inactivated human serum or normal human serum. Data are presented as the mean ± SEM and are representative of 3 independent experiments each with 3 biological replicates. Statistical significance was assessed by an unpaired *t*-test (*$p<0.05$, ***$p<0.001$, ****$p<0.0001$).
