# Supplementary material for: Fitness factor genes conserved within the multi-species core genome of Gram-negative Enterobacterales species contribute to bacteremia pathogenesis
Source: PLoS Pathog. 2024 Aug 23;20(8):e1012495. doi: 10.1371/journal.ppat.1012495 (PMC11376589; doi:10.1371/journal.ppat.1012495)
Supplement: S6 Fig — Bacterial strains with mutations in the genes arcA, ruvA, and xerC in each species of interest were exposed to hydrogen peroxide to measure resistance to oxidative stress. Percent survival was calculated by comparing bacterial survival at 2 hours to the input. Fold change was calculated by dividing percent survival of each mutant to its respective wild-type strain. No mutant conveyed statistically significant resistance to hydrogen peroxide stress as assessed by a one-sample t-test with a hypothetical value of 1, representing survival of the wild-type strain. Data are means of 3 independent experiments. (PDF) [file ppat.1012495.s006.pdf]

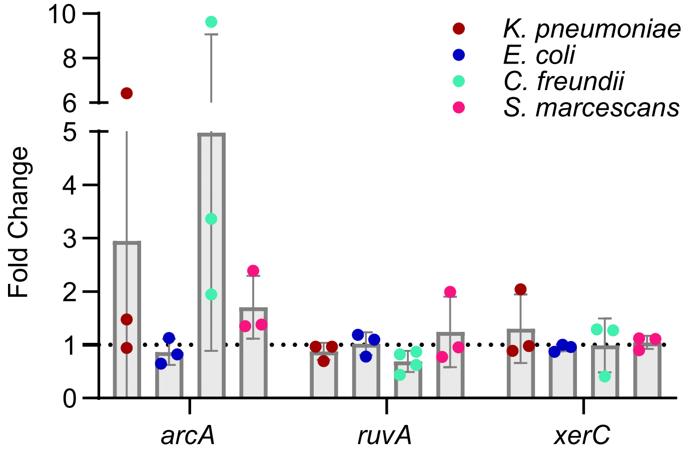

Supplemental Fig 6. Oxidative stress by exposure to H_2O_2. Bacterial strains with mutations in the genes *arcA*, *ruvA*, and *xerC* in each species of interest were exposed to hydrogen peroxide to measure resistance to oxidative stress. Percent survival was calculated by comparing bacterial survival at 2 hours to the input. Fold change was calculated by dividing percent survival of each mutant to its respective wild-type strain. No mutant conveyed statistically significant resistance to hydrogen peroxide stress as assessed by a one-sample t-test with a hypothetical value of 1, representing survival of the wild-type strain. Data are means of 3 independent experiments.
