# Supplementary material for: Fitness factor genes conserved within the multi-species core genome of Gram-negative Enterobacterales species contribute to bacteremia pathogenesis
Source: PLoS Pathog. 2024 Aug 23;20(8):e1012495. doi: 10.1371/journal.ppat.1012495 (PMC11376589; doi:10.1371/journal.ppat.1012495)
Supplement: S7 Fig — C. freundii UMH14 wild-type and prc::nptII grown aerobically in either LB medium (1% NaCl LB) or a hypotonic LB medium (0% NaCl LB) maintaining either the pBBR1MCS-5 empty vector plasmid (Vector) or a pBBR1MCS-5 derivative encoding the prc locus under control of its native promoter (pprc). Representative growth curves are the mean and standard deviation (small and obscured by symbols) of technical triplicate wells derived from OD600 measurements taken every 15 m for 10 h in a plate reader. (PDF) [file ppat.1012495.s007.pdf]

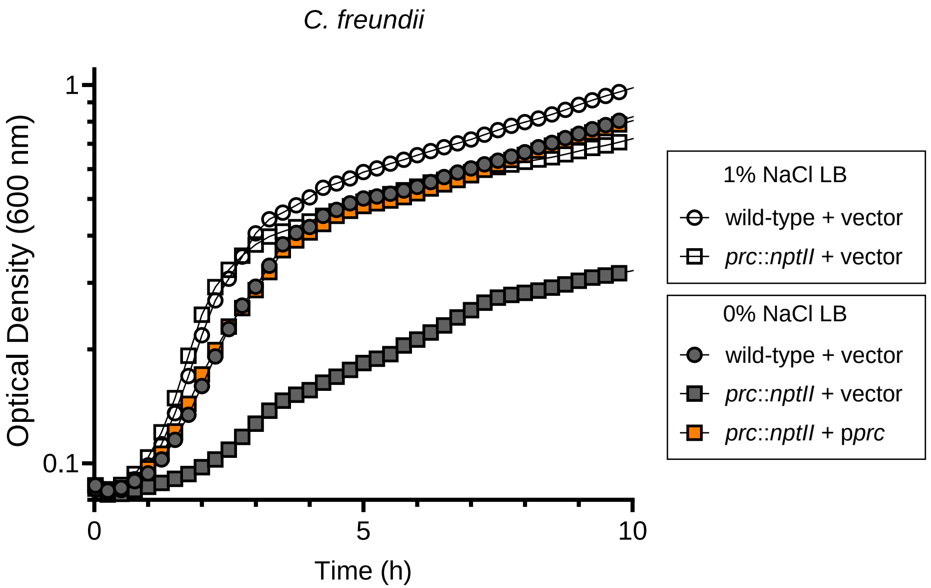

Supplementary Fig 7. Complementation of a *C. freundii* UMH14 *prc* dependent growth defect in a hypotonic medium. *C. freundii* UMH14 wild-type and *prc*::*nptII* grown aerobically in either LB medium (1% NaCl LB) or a hypotonic LB medium (0% NaCl LB) maintaining either the pBBR1MCS-5 empty vector plasmid (Vector) or a pBBR1MCS-5 derivative encoding the *prc* locus under control of its native promoter (p*prc*). Representative growth curves are the mean and standard deviation (small and obscured by symbols) of technical triplicate wells derived from OD_{600} measurements taken every 15 m for 10 h in a plate reader.
