# Supplementary material for: Fitness factor genes conserved within the multi-species core genome of Gram-negative Enterobacterales species contribute to bacteremia pathogenesis
Source: PLoS Pathog. 2024 Aug 23;20(8):e1012495. doi: 10.1371/journal.ppat.1012495 (PMC11376589; doi:10.1371/journal.ppat.1012495)
Supplement: S1 Table — (PDF) [file ppat.1012495.s008.pdf]

Supplemental Table 1. Primer sequences used for construction of prioritized fitness gene mutants.

Name	Sequence (5'-3')
SM *atpIBEFHAGDC* KO F	GATCGCTCAAATTGCTAAAGACGGTTTGGTTGACCATCGGCTATAAATCAgTGTAGGCTGGAGCTGCTTC
SM *atpIBEFHAGDC* KO R	CCCGATATCAGGGGTTATACACCACACCGCCCGCCTGGCTGAAATGCTTACATATGAATATCCTCCTTAGT
SM *atpIBEFHAGDC* confirm F	CGTTCGACGGCGTGATCAGC
SM *atpIBEFHAGDC* confirm R	CGTCCAGCTTGACCGTCAGC
SM *arnBCADTEF* KO F	TTAAGGTTTAGTTAAGCTTGCGGCCTTACATTGTCAGTTAATCCACGGAAgTGTAGGCTGGAGCTGCTTC
SM *arnBCADTEF* KO R	CCGGCACCACGTAAGGAGAGCGCTATTTGGCGTTAATTTATCTCCCGCACCATATGAATATCCTCCTTAGT
SM *arnBCADTEF* confirm F	TTATCTCAGCCAGCAACAGC
SM *arnBCADTEF* confirm R	GTCGTTGGTATCGTAAATGC
SM *gmhB* KO F	GGTAAAACATGCGATACTGTCAGCGTATACAGGCCCTCAGGAGCAAAGCGGTGgTGTAGGCTGGAGCTGCTTC
SM *gmhB* KO R	TCTGACCGCTCACTATTCATTCGTAACGCCTATTAAACCCGCTTTTTGATCATATGAATATCCTCCTTAGT
SM *gmhB* confirm F	TGGCTTCATCGCACAGCAGC
SM *gmhB* confirm R	GCTGCTGGCACGGAGTTAGC
SM *ubiH* KO F	cgccgatgccatcgaggctttgatggcggcggcaaggtaacggaatgagcGTGTAGGCTGGAGCTGCTTC
SM *ubiH* KO R	cgattctgtgtctaacgttccacccagcccagcgtgcgcttggcgaaggcCATATGAATATCCTCCTTAGT
SM *ubiH* confirm F	gtacgacattgtgctggcgt
SM *ubiH* confirm R	gtaatcagcgtgatgtccgaca
SM *wzxE* KO F	CAATCAGCGTACGGTAATCAATACCATCCTGAGTTTCTTTGCCTGATATGgTGTAGGCTGGAGCTGCTTC
SM *wzxE* KO R	GATATCGGATCCCAGAACGTGAATCAGTGTGGTCATACTCGTCTACGATACATATGAATATCCTCCTTAGT
SM *wzxE* confirm F	GTTCGTGGTGGAAGATGCCG
SM *wzxE* confirm R	CGCTTCGATATGCCGATTGC
SM *ruvA* KO F	atgcccggtcattctgcaggagggtaaatgataggtcgtctcagaggaGTGTAGGCTGGAGCTGCTTC
SM *ruvA* KO R	aggcgatcggcttcaatcattatctacctcttagcggccttacaaagcCATATGAATATCCTCCTTAGT
SM *ruvA* confirm F	ccagaatgtgctgcggatg
SM *ruvA* confirm R	ttcgttggccacgatgttgg
SM *aroC* KO F	gcacggcgggcctcataacgataaaagactaacggtaaggagccgtgatgGTGTAGGCTGGAGCTGCTTC
SM *aroC* KO R	caggcccaacatccagtttttcatggcgattaccagcgcggcacgtcggaCATATGAATATCCTCCTTAGT
SM *aroC* confirm F	gcgtgctgatttgtgaagtg
SM *aroC* confirm R	acctggtagttcggtgagt
SM *pdxA* KO F	cttacgtgaagatcctcgatggcagcgatgcacagcaataaacgcgtcGTGTAGGCTGGAGCTGCTTC
SM *pdxA* KO R	tggtggactctgttattcattacaattaattatcattttaatggcgagCATATGAATATCCTCCTTAGT
SM *pdxA* confirm F	gaaagatcgcgcttaccgc
SM *pdxA* confirm R	cggagacaatgctatcgatg
SM *apaGH* KO F	tggctgtcagccaatccggcgccgcagcaataaggagttgttgtcatgGTGTAGGCTGGAGCTGCTTC
SM *apaGH* KO R	gcccttgccttccagcgaggcccagtggccgaagacgatggtgtattcCATATGAATATCCTCCTTAGT
SM *apaGH* confirm F	ttcaaccagcggcggaaaac
SM *apaGH* confirm R	cggactacaagacgatgttcg
SM *lpdA* KO F	taaaaatgacgtctgacccgccggacaatcaattaagaggtcatgatgGTGTAGGCTGGAGCTGCTTC
SM *lpdA* KO R	gccgcttgttaaccggtcaaaccagcagaattacttcttcttggctttCATATGAATATCCTCCTTAGT
SM *lpdA* confirm F	gccgtctggtgatgtaatg
SM *lpdA* confirm R	tttgctgaatcgttgagcgc

SM *hscBA* KO F	CTTGCGTCCCGCTTCCAGGATCTGCAGCGCCAATTTCACCCCGATCGTTTgTGTAGGCTGGAGCTGCTTCGaag
SM *hscBA* KO R	AATGGAAGCGTCCATGCGGCGCGCGGCAAAATCTTGCGTTTGTGCATCTACATATGAATATCCTCCTTAGTtcct
SM *hscBA* confirm F	CTGGAATTTGTTGACGAAGC
SM *hscBA* confirm R	TATTGGTCGTACAGGGCTTC
SM *sapABCDF* KO F	CAGAGTGCGATACACTAACGCTATTGCTGATTAACCCATGAGCCTCTATGgTGTAGGCTGGAGCTGCTTC
SM *sapABCDF* KO R	CGCAGCGGGTTTATTGCAGGCCAGGACGGGCCTCAAAAACGGCCGCCGTCCATATGAATATCCTCCTTAGT
SM *sapABCDF* confirm F	TCGTTCATACATCCCCTTGC
SM *sapABCDF* confirm R	CGAAGGCTGTTCATCAGTGC
SM *prc* KO F	acttgcagttctgatacggaggccaacccaggcatgaacaaattcGTGTAGGCTGGAGCTGCTTCG
SM *prc* KO R	tggctgctgagcgggccgatctttttccagatgagccagctccagCATATGAATATCCTCCTTAGT
SM *prc* confirm F	agtcagagcaggcaagag
SM *prc* confirm R	ataaatttgtcaggcgacgc
SM *xerC* KO F	tctcaaggtagaacggcaactcagcccgctgacgcagcttGTGTAGGCTGGAGCTGCTTC
SM *xerC* KO R	acacgttcgccagatgttgaaagtcgaggtgggtataaatCATATGAATATCCTCCTTAGT
SM *xerC* confirm F	gcgatacgcaacactatcag
SM *xerC* confirm R	tgacgtcgtggtagatctcc
SM *proP* KO F	acgctttcacgaacaacgaataattcattaagggatgattatatgGTGTAGGCTGGAGCTGCTTC
SM *proP* KO R	cgggtggcttttgtgtttcgcgcggatcaatccagcttgggatgCATATGAATATCCTCCTTAGT
SM *proP* confirm F	taaccttggcataaacgacg
SM *proP* confirm R	atggaaaggtccatcgtgctc
SM *pstSCABphoU* KO F	ATTAAAACGACTCGAAGTCGATTTTAAATATCCCATTAGGAGGGATTATGgTGTAGGCTGGAGCTGCTTC
SM *pstSCABphoU* KO R	ACCGCGCCTTTGAAGGATATTACCCGGAAAGGGTCAGTCGGCTTTGTCGTCCATATGAATATCCTCCTTAGT
SM *pstSCABphoU* confirm F	GCAGCTGCTGTCTTACCACG
SM *pstSCABphoU* confirm R	AGTTCACTTCCTTGGCGCGC
SM SufI F	AGATTAAAGAGGAGAAAGAGTCCcATGTCACTCAGTCGACGTCA
SM SufI R	ccctctgtcaaggcagtagcGGGTAATGGGGTCTGCGTTC
Dasher GFP F	gctactgccttgacagaggg
Dasher GFP R	TgGGACTCTTTCTCCTCTTTAATCT
KP *atpIBEFHAGDC* KO F	AAAGGACGTTTTATACGACACGCGACATACCTCAAAGGGAGCAGGAGTAAAAACGTGATGGTGTAGGCTGGAGCTGCTTC
KP *atpIBEFHAGDC* KO R	CCAGCCTGGAACCCAGACTGGCTTTTTACGTTCAAGCCGGTGTTACATCGCTTTTTTGGTCATATGAATATCCTCCTTAGT
KP *atpIBEFHAGDC* confirm F	GATGCTTGACTCTTCGTC
KP *atpIBEFHAGDC* confirm R	TTCACTTCATCCGCTC
KP *arnBCADTEF* KO F	ATATTTTATTCTAACGACCCAGATTCGTGCGTGGGTCATCTACAGAACAGGATGATGATGGTGTAGGCTGGAGCTGCTTC
KP *arnBCADTEF* KO R	GATCCTGCTCGGCCCGGGCCGTCCCAAAAGCCGGCTCCCCGGCTAGCGGCGTTTCACCGGCATATGAATATCCTCCTTAGT
KP *arnBCADTEF* confirm F	GCCGTGCTGAACTCACT
KP *arnBCADTEF* confirm R	GCCATTAACCAGCGTC
KP *wzxE* KO F	CAATCTGTCTGTGGTGAACCAGCGGACGGTGATTAACTCGCTGCTGAGCTACTTCTCCTGATATGGTGTAGGCTGGAGCTGCTTC
KP *wzxE* KO R	CTGGTTATGATGCGGGATATCCGATCCCAGTACATGTATCAGTGCTGTCATGCCCGTTTACGCCACATATGAATATCCTCCTTAGT
KP *wzxE* confirm F	CATTACATTCCCCTGCACAG
KP *wzxE* confirm R	CAGTTCGTCGTTAAAGAACC
KP *ruvA* KO F	ATCCAGCCTTTTTTTATGATACCGCCAGATACTTAGACCATAACGCAGGAGCCCCACGTGGTGTAGGCTGGAGCTGCTTC

KP *ruvA* KO R	TGTCTGCCGATACCAGCCGGTCTGCTTCAATCATCCCTTACCTCACAACGCAGCGCGCAGCATATGAATATCCTCCTTAGT
KP *ruvA* confirm F	TCAGATCAGCGAGACC
KP *ruvA* confirm R	CGACATACTCTGCCAGCA
KP *apaGH* KO F	GTACTGCCTGATGGCCAACTGGCTGTCTGACAATCTGCCAACGAAGGAGAGCTAACCATGGTGTAGGCTGGAGCTGCTTC
KP *apaGH* KO R	GGTAGCCCGGATAAGGCGCAACGCGCCGCTATCCGGGAAGAGTCAGGACGCGACGGCTTCCATATGAATATCCTCCTTAGT
KP *apaGH* confirm F	GTATCCGGTCAAAGAGATCC
KP *apaGH* confirm R	TAGGTCGGGTAAGGCGTTAG
KP *lpdA* KO F	ACTTGGGCGTAAGAATAATAGCGTCAGACCCGCCGGATATAAATTACAGAGGTCATGATGGTGTAGGCTGGAGCTGCTTC
KP *lpdA* KO R	CGGCTCCCGGAGCCGCTTATGCTTTTTTCCTGAAAGAAAAAGTTACTTTTTCTTCGCTTTCATATGAATATCCTCCTTAGT
KP *lpdA* confirm F	ATACACGCGGATAGAACGTC
KP *lpdA* confirm R	GTAGCGGTTTATGAACAGTGAC
KP *hscBA* KO F	AACGTATAATCGGTACTCCTCAACCCCACCGCGGTCACGCCGCGCGTGGGGTTTTAATTTAGATGGTGTAGGCTGGAGCTGCTTC
KP *hscBA* KO R	GGGGCAGAAATACAATTTTTGGCATTTTAAACCTCGTCCACGGATTGGCCTTTCAGCGCGATACGCATATGAATATCCTCCTTAGT
KP *hscBA* confirm F	TCAAAGATGAGTGCGGCTGC
KP *hscBA* confirm R	TCTCAGCTTCCACAACGATG
KP *sapABCDF* KO F	CGGTGGTGAATGTCTATTAATCTTAATAAGATAGAGTTCAGCTCTATAATCAAACAGAAAATATGGTGTAGGCTGGAGCTGCTTC
KP *sapABCDF* KO R	TGAATGACAAGGGCGGCGTGAGGGCCGCCCCTGCCGTTGCCTGCCAGCTATTTCCCGTCTTTGCGCATATGAATATCCTCCTTAGT
KP *sapABCDF* confirm F	GTAACCGCATTATCCCTCAG
KP *sapABCDF* confirm R	CGATGTCTCATGGATAATAGG
KP *prc* KO F	GGTATGTCAATGATTGTACGCGCAGAACACCTGGTGTTCTGAAACGGAGGCCGGGCCAGGCATGAGTGTAGGCTGGAGCTGCTTC
KP *prc* KO R	AACGCGCACAAAAAAAACGGGCACCTCTCAGGGTGCCCGTTAGCAACTTATTTGCTGGCCGGCGGCATATGAATATCCTCCTTAGT
KP *prc* confirm F	CACCAAAGATGGCGTTCGTG
KP *prc* confirm R	TTGTAGCATCTTGCTGACAG
KP *xerC* KO F	CAGCTGTTGCAGGAGATTGCCCAGATGCTGCCCGGCCTGCTGGAGCGCTGGATTGAACGCGCATGGTGTAGGCTGGAGCTGCTTC
KP *xerC* KO R	GTCAAACGTCAGCGCGGAAATCTGGCCCAGAGGTCGGTAAAAACGCATTATTTCCCCCGTTTAGCCATATGAATATCCTCCTTAGT
KP *xerC* confirm F	GGGTGATGCTCTTCAGCAGC
KP *xerC* confirm R	TGAGCGAAGGATGGTAGCTG
KP *proP* KO F	GCGAATGGCCTTGCCATTCGGCGAAGAGGCGTGCACTCAAGGCGGAGTAGCAGAGGAAAAATATGGTGTAGGCTGGAGCTGCTTC
KP *proP* KO R	AATGCCGCCGGGGTTTCCCCAGGCGGCATGATTAACAGTGAAAACGGTTAGTCGTTGATACGCGGCATATGAATATCCTCCTTAGT
KP *proP* confirm F	AGCAATTCACCGCTCCTGAC
KP *proP* confirm R	TCCAGCAGTCGCTTCTACAG
KP *pstSCABphoU* KO F	TTGACCTATTTTGCTCACCATAGCCTCGAAACAATGATTTACGAATCCCAGCAGGAGACATTATGGTGTAGGCTGGAGCTGCTTC
KP *pstSCABphoU* KO R	TATCTAATTCTGATAAATCCATTTACCAGAATTAGACAGAATGATTATTACTCTTTCGGATCTTTCATATGAATATCCTCCTTAGT
KP *pstSCABphoU* confirm F	TACGAATCCCAGCAGGA
KP *pstSCABphoU* confirm R	GCGGGGTAAAACATATC
KP *tatC* KO F	gtccgctacggcggcagttccccattcttcccccgcatcgagtgataaaccgtaaacatgGTGTAGGCTGGAGCTGCTTC
KP *tatC* KO R	tcgctcccatatgccaaccgccctgacgggcggttggcggtattactcttctctgtgctcCATATGAATATCCTCCTTAGT
KP *tatC* confirm F	taccatccttaatccggtgg
KP *tatC* confirm R	gctcgccagtttctgcg
KP kan insert confirm R	ATGCTCTTCGTCCAGATCATC
KP *ubiH* KO F	TGGCCATCTCCCGTTTGACCGGCGGCGCGCTGCCGGTCCACCTCATTGAAGCGCAGGATCCGCATGTGTAGGCTGGAGCTGCTTC

KP *ubiH* KO R	ACGGGGAACCCAACCGAGGGTACGCTGCGCCAGCGCATCGCGCGCCGGGGTAAATAATTCCAATGGGAATTAGCCATGGTCC
KP *ubiH* confirm F	GGCGAGATTGACGAGCTTAT
KP *ubiH* confirm R	CCACACTTCCATACCGTGATAG
KP *aroC* KO F	ATGGCAGGAAATACAATTGGACAACTCTTTCGCGTCACTACCTTCGGCGAATCGCACGGTCTGGCGTGTAGGCTGGAGCTGCTTC
KP *aroC* KO R	ATAAAGTGATCCATCAGAACGATCGCCAGCATCGCCTCAGCGATCGGTACGGCGCGGATGCCGACATGGGAATTAGCCATGGTCC
KP *aroC* confirm F	CTTTATGCTGACCAAACAGCAG
KP *aroC* confirm R	AGATCCGGATGACCGAAGTA
EC *ruvA* KO F	TATCCAGCATTTTTTTATCATACAGCATTGGCAGAGTGTAGGCTGGAGCTGCTTC
EC *ruvA* KO R	AATCATCCTTTACCTCATAACGCGGCGCGTAGGGCCATATGAATATCCTCCTTAGT
EC *ruvA* confirm F	CACATTTCTTCGCTGAATATGTAAT
EC *ruvA* confirm R	TGGCAAGCGTAGTTTTACCC
EC *pdxA* KO F	GCCAGCGCCTACGTTAAAATCCTGAGCAACTAATGGTGTAGGCTGGAGCTGCTTC
EC *pdxA* KO R	CTGGTGGACTCGATTATTCATTGGGTGTTAACAATCATATGAATATCCTCCTTAGT
EC *pdxA* confirm F	GCTGGATACCCGTAATGTCG
EC *pdxA* confirm R	AAACAATGCTGTCGATCACG
EC *apaGH* KO F	AGAACGCGCCTTTGCAGGAGAGTTAACGATGATCAGTGTAGGCTGGAGCTGCTTC
EC *apaGH* KO R	AGCATTAGATTTTATGCTCGACGGGATTTAAGACGCATATGAATATCCTCCTTAGT
EC *apaGH* confirm F	CCTGTTTAGCGTCGAGGTGT
EC *apaGH* confirm R	CATGACGCATGAGACTGCAAC
EC *lpdA* KO F	TAATTTGCCGGATGTTCCGGCAAACGAAAAATTACTTCATATGAATATCCTCCTTAGT
EC *lpdA* KO R	CCGCCGGAGATAAATATATAGAGGTCATGATGAGTGTGTAGGCTGGAGCTGCTTC
EC *lpdA* confirm F	CTTATCGGGCCTACACAAGC
EC *lpdA* confirm R	AAATCGGTTGCCGTTTGTT
EC *sapABCDF* KO F	TGCTCCTCGCTAAGTTGTTCTGTGAGTTATAGTTAGTGTAGGCTGGAGCTGCTTC
EC *sapABCDF* KO R	GAGGGGGGCCTGTCAGGATGAGGTGCAAAACACTACATATGAATATCCTCCTTAGT
EC *sapABCDF* confirm F	TCCCGTACGCATTGATGAGA
EC *sapABCDF* confirm R	ATTGCTCACGGTCTTATGCG
EC *arnBCADTEF* KO F	ACTCAGTAACGTTGTTTTGTTGTGGGCAGGAAAATCATATGAATATCCTCCTTAGT
EC *arnBCADTEF* KO R	TGGACAGGTATTCAATGACGGATGGAAAAAAAATGGTGTAGGCTGGAGCTGCTTC
EC *arnBCADTEF* confirm F	GGGGATTTACTCTCGCCTTT
EC *arnBCADTEF* confirm R	TTGCCAAATGCAAAGTCTAAA
EC *prc* KO F	CGGAGGCCGGGCCAGGCATGAACATGTTTTTTAGGGTGTAGGCTGGAGCTGCTTC
EC *prc* KO R	AATCAGGCACAATTTCTTGTGCCTGATTGATATTACATATGAATATCCTCCTTAGT
EC *prc* confirm F	ACACCCCGGTTTCTGACATT
EC *prc* confirm R	TGCGTAAGTCACCAGAAAGAC
EC *proP* KO F	AAATCGGCAATCTCGTGGTCAATATCATCGATTTTCATATGAATATCCTCCTTAGT
EC *proP* KO R	GCGGAAATGGGCCAACTCTGCGAGGAAAGCTATGCGTGTAGGCTGGAGCTGCTTC
EC *proP* confirm F	GGACCGGGTATTGATGAGAGT
EC *proP* confirm R	GGGCAATGGTCGAGGAAATC
EC *pstSCABphoU* KO F	ACAATGCTTTATGAATCCTCCCAGGAGACATTATGGTGTAGGCTGGAGCTGCTTC
EC *pstSCABphoU* KO R	AATAATTAAATTATTGGGATTTACTTGGTGAATTACATATGAATATCCTCCTTAGT

EC *pstSCABphoU* confirm F	ATCCTGCCAACTCCATGTGA
EC *pstSCABphoU* confirm R	GCATGTTCATAGCAAGGACCT
EC *ECAoperon* KO F	TCTGCTAATAATTTTCTCTGAGAGCATGCATTGTGGTGTAGGCTGGAGCTGCTTC
EC *ECAoperon* KO R	TTCGTCGCTTTACAAAGAGAGGAAAATCAGAGGTT
EC *ECAoperon* confirm F	AGAAAATGTGGATCCTGCGC
EC *ECAoperon* confirm R	CCGCCATCCACATAAACCAG
EC *atpIBEFHAGDC* KO F	atacctcgaagggagcaggagtgaaaaacgtgatgGTGTAGGCTGGAGCT
EC *atpIBEFHAGDC* KO R	tgcttttcaagccggtgttacatcgctttttggtCATATGAATATCCTCCTTAGT
EC *atpIBEFHAGDC* confirm F	ACCGCTTTTTGATGCTTG
EC *atpIBEFHAGDC* confirm R	TTCAGGACGGGTCAATAC
EC *gmhB* KO F	gcgtcacatgccttattaaggagctataaaaggtgGTGTAGGCTGGAGCTGCTTC
EC *gmhB* KO R	cgctcatcttttaatcatcacgccggtttttgctgCATATGAATATCCTCCTTAGT
EC *gmhB* confirm F	CAGACGTCTAAATCAATCAGG
EC *gmhB* confirm R	GATGGAGGCGCATTATAG
EC *wzxE* KO F	aattgcgaccttgttgaactacttctcctgatatgGTGTAGGCTGGAGCTGCTTC
EC *wzxE* KO R	tacgtgaatcagtacagtcatgcccgcctacgccaCATATGAATATCCTCCTTAGT
EC *wzxE* confirm F	CTACACCACCAAAGAGAG
EC *wzxE* confirm R	TCGTTGAAAAACCGCAG
EC *hscBA* KO F	gttatcgcggcggatcgcagccctgagaatgttatgGTGTAGGCTGGAGCTGCTTC
EC *hscBA* KO R	ataacaatctttggcatattaaacctcgtccacggaCATATGAATATCCTCCTTAGT
EC *hscBA* confirm F	cgaaagcttccacgttt
EC *hscBA* confirm R	caatctcgataccgttacg
EC *xerC* KO F	ggcgcgttggattgaacgcgtatgaccgatttacacGTGTAGGCTGGAGCTGCTTC
EC *xerC* KO R	aaggccggtaaaaacgcattatttcccccgtttggcCATATGAATATCCTCCTTAGT
EC *xerC* confirm F	gatttgggtgttgtgct
EC *xerC* confirm R	tcggtgcgcaaaatca
EC *tatC* KO F	ccttccccttcgtcgagtgataaaccgtaaacatgGTGTAGGCTGGAGCTGCTTC
EC *tatC* KO R	ctgacgggcggttgaatttattcttcagtttttcCATATGAATATCCTCCTTAGT
EC *tatC* confirm F	AACAGAAGCCAGAAACTAC
EC *tatC* confirm R	CGAGCTGGTCAAATTAAC
EC *ubiH* KO F	GGGCACGACGCTGGCGCTGGCTATTTCCCGGTTAAGTCACGTGTAGGCTGGAGCTGCTTC
EC *ubiH* KO R	ACATCGCGTGCTGGGGTAAATAATTCCATCGTCATCAGCCATATGAATATCCTCCTTAGT
EC *ubiH* confirm F	GAACCAGGGCTGTATATTGCG
EC *ubiH* confirm R	CATCCGCCGCCAGAGG
EC *aroC* KO F	AAAGATTAAGTAAACACGCAAACACAACAATAACGGAGCCGTGTAGGCTGGAGCTGCTTC
EC *aroC* KO R	TTACCAGCGTGGAATATCAGTCTTCACATCGGCATTTTGCATATGAATATCCTCCTTAGT
EC *aroC* confirm F	GGCAGATTACCTCGCTGATGA
EC *aroC* confirm R	TCAGCACCGACGATACAGC
EH *atpIBEFHAGDC* KO F1	**cgaagggagcaggag**taaaaacgtgtgcatgatcgaattagcttcaaaag
EH *atpIBEFHAGDC* KO F2	gttttatacgacacgcggcatacct**cgaagggagcaggag**
EH *atpIBEFHAGDC* KO R1	**gactggcttttgtgcttttc**aagccggtggacgatcgaattggggatctt

EH *atpIBEFHAGDC* KO R2	aaaaagccagcctgggatcca**gactggcttttgtgcttttc**
EH *atpIBEFHAGDC* confirm F	cgcttgggtcaaaagttg
EH *atpIBEFHAGDC* confirm R	gaatacatgcgggtacctttg
EH *arcA* KO F1	**cactcgaggtttacat**atgaacaaagacgatcgaattggggatctt
EH *arcA* KO F2	gacccttgatcaatttgaaatcgga**cactcgaggtttacat**
EH *arcA* KO R1	**ctcctgtttaagt**taagactttgcatgatcgaattagcttcaaaag
EH *arcA* KO R2	cgataaagaaattaggcatgtcttaacgg**ctcctgtttaagttaa**
EH *arcA* confirm F	gttgccgaaacaccctg
EH *arcA* confirm R	cgataacctgcgtaacg
EH *gmhB* KO F1	**cgaacaagcctttttcaggagc**aaaccggacgatcgaattggggatctt
EH *gmhB* KO F2	ctgcgattcatgagatactagc**cgaacaagcctttttcaggagc**
EH *gmhB* KO R1	**tatttcaaccgctcaccttttcgccaaaac**gctgcatgatcgaattagcttcaaaag
EH *gmhB* KO R2	agcggaaaaatgcatttt**tatttcaaccgctcaccttttcgccaaaac**
EH *gmhB* confirm F	cttctgccagatacggg
EH *gmhB* confirm R	caggattttttctcttcaaccg
EH *ubiH* KO F1	**caacattttgcacggtcaatactcc**gacgatcgaattggggatctt
EH *ubiH* KO F2	attccgccgcccacaatggcgacat**caacattttgcacggtcaatactcc**
EH *ubiH* KO R1	**aggcgctgatggcggcggcacgcgttgc**atgatcgaattagcttcaaaag
EH *ubiH* KO R2	gtggtgaaaaaagccgacgacatcg**aggcgctgatggcggcggcacgcgt**
EH *ubiH* confirm F	gtcagtagcttttcgctgg
EH *ubiH* confirm R	catggtgctgaccgttg
EH *wzxE* KO F1	**ctgagttatttcgcctg**atgacgatcgaattggggatctt
EH *wzxE* KO F2	ggtgaaccagcgtacggtgatcaattcgctt**ctgagttatttcgcctg**
EH *wzxE* KO R1	**gtgtatcagtgcagtcattg**catgatcgaattagcttcaaaag
EH *wzxE* KO R2	tggttgtgatgcgggatatccgatcccagtat**gtgtatcagtgcagtcattg**
EH *wzxE* confirm F	gctggcgaagccttc
EH *wzxE* confirm R	cgttatcgcgtccaac
EH *ruvA* KO F1	**cctttacctcacaatgcag**cgcgcagacgatcgaattggggatctt
EH *ruvA* KO F2	ataccaggcggtctgcttcaatcatc**ctttacctcacaatgcag**
EH *ruvA* KO R1	**ttccagaacgcaggagc**gtcacgtgtgcatgatcgaattagcttcaaaag
EH *ruvA* KO R2	ttttattatgtcggcagataatttc**ttccagaacgcaggagc**
EH *ruvA* confirm F	cctggatgaaaatctccatctg
EH *ruvA* confirm R	cattgcgattacccactgtc
EH *aroC* KO F1	**agagcgattgcggttttttttcatag**gacgatcgaattggggatctt
EH *aroC* KO F2	tgctccactgaccagcagcgccagc**agagcgattgcggttttttttcatag**
EH *aroC* KO R1	**cacaaacaacaacatcggagccgtg**tgcatgatcgaattagcttcaaaag
EH *aroC* KO R2	tcggcccgtttcacaacgctcaaa**cacaaacaacaacatcggagccgtg**
EH *aroC* confirm F	gtgcgcatcacctgatag
EH *aroC* confirm R	catggtacatctgatagagcag
EH *pdxA* KO F1	**gactcgattagtcattgagtattaac**aatgacgatcgaattggggatctt
EH *pdxA* KO F2	acgggctaagtggccctgatg**gactcgattagtcattgagtattaac**

EH *pdxA* KO R1	**tcaccccggcgaacc**cgccgggatttgcatgatcgaattagcttcaaaag
EH *pdxA* KO R2	ctgatgaaaccgcatcgtgttgtga**tcaccccggcgaacc**
EH *pdxA* confirm F	gttcgtcgaggcgttc
EH *pdxA* confirm R	cagctgatggacacgc
EH *apaGH* KO F1	**gaagggagaggataaacatc**tgacgatcgaattggggatctt
EH *apaGH* KO F2	aacgtcgttaacggttttatcctctcctc**gaagggagaggataaacatc**
EH *apaGH* KO R1	**gacaatgcgccgccgaag**gagagctaagcctgcatgatcgaattagcttcaaaag
EH *apaGH* KO R2	agctggctaattacatcagc**gacaatgcgccgccgaag**
EH *apaGH* confirm F	cgcagaaactgtatctgacg
EH *apaGH* confirm R	tgctgagccgtatcacg
EH *lpdA* KO F1	**gggtatacattaagaggtcatgatg**tgcatgatcgaattagcttcaaaag
EH *lpdA* KO F2	cggatagaacgacccggtggacgac**gggtatacattaagaggtcatgatg**
EH *lpdA* KO R1	**tctgaagaaacttatttcttcttcgc**tttgacgatcgaattggggatctt
EH *lpdA* KO R2	ggcctgaggccgctttttca**tctgaagaaacttatttcttcttcgc**
EH *lpdA* confirm F	ctggtgatgtaatcgaaaagc
EH *lpdA* confirm R	ggtaatgagctttgtcgttaac
EH *hscBA* KO F1	**tctttggcatattaaacctcgtc**cacggagacgatcgaattggggatctt
EH *hscBA* KO F2	ccgcatgaggcaaaattacaa**tctttggcatattaaacctcgtc**
EH *hscBA* KO R1	**acaggctacccctgag**attgttatgtgcatgatcgaattagcttcaaaag
EH *hscBA* KO R2	cccgctacgcgtggggtttgttctg**acaggctacccctgag**
EH *hscBA* confirm F	gtcgaaaccttcacgcac
EH *hscBA* confirm R	caattcctcaacggcactc
EH *sapABCDF* KO F1	**atcgaatctaaaaaccttaaaaact**tgcatgatcgaattagcttcaaaag
EH *sapABCDF* KO F2	ggcgtaagtgcgatacactttgcagatcga**atctaaaaaccttaaaaact**
EH *sapABCDF* KO R1	**actctgctggcaagcg**ggttacgcaacgggacgatcgaattggggatctt
EH *sapABCDF* KO R2	ccgcatgccatagaggtgttt**actctgctggcaagcg**
EH *sapABCDF* confirm F	cctgaaagaggcgaaatataatc
EH *sapABCDF* confirm R	gagtcgacccgtcagac
EH *prc* KO F1	**cctgttggggattattttgtcg**gagcagggacgatcgaattggggatctt
EH *prc* KO F2	aaacaggcacaatttcttgtg**cctgttggggattattttgtcg**
EH *prc* KO R1	**tgaaacggaggccgag**cctggcatgtgcatgatcgaattagcttcaaaag
EH *prc* KO R2	tgtacgcgcagaacacttgttgttc**tgaaacggaggccgag**
EH *prc* confirm F	cctcagtttaacaaaacgcac
EH *prc* confirm R	cggtttctgacatcaatgc
EH *xerC* KO F1	**tggagcgctggattgag**cgcgtatgtgcatgatcgaattagcttcaaaag
EH *xerC* KO F2	atcgccctgatgttgcctgagttgc**tggagcgctggattgag**
EH *xerC* KO R1	**gtaaaaacgcattatttcccccg**tttggcgacgatcgaattggggatctt
EH *xerC* KO R2	tgagatctgaccgagtgggcg**gtaaaaacgcattatttcccccg**
EH *xerC* confirm F	gccggaagccaaagc
EH *xerC* confirm R	cagggactgacgcag
EH *proP* KO F1	**ggctgagtctacgaggaaag**ctatgtgcatgatcgaattagcttcaaaag

EH *proP* KO F2	gcaaaatgcgctgtgaaacggacgg**ggctgagtctacgaggaaag**
EH *proP* KO R1	**cgggtttttatcactcgttaattc**tcgggacgatcgaattggggatctt
EH *proP* KO R2	taaaagaaaacccgccagcgg**cgggtttttatcactcgttaattc**
EH *proP* confirm F	cgctggtagggtaaaaagg
EH *proP* confirm R	gcacgtccctgaactg
EH *pstSCABphoU* KO F	tcatcgtagccacttaaacaatgatttacgaaatccttgcaggagacatttgcatgatcgaattagcttcaaaag
EH *pstSCABphoU* KO R	accgaatggtcccctctccctgtgggagagggttagggtgagggtaaaaagacgatcgaattggggatctt
EH *pstSCABphoU* confirm F	gaaccagactaaacatgcttattgtg
EH *pstSCABphoU* confirm R	cgatccgcacaacaagc
CF *atpIBEFHAGDC* KO F	CGCGGCATACCTCGAAGGGAGCAGGAGTAAAAACGTGATGGTGTAGGCTGGAGCTGCTTC
CF *atpIBEFHAGDC* KO R	CTGGAAACCAGACTGGCCTTTGTGCTTTTCAAGCCGGTGCATATGAATATCCTCCTTAGT
CF *atpIBEFHAGDC* confirm F	GCATAAAAGTGATAAATGTGGCGTAG
CF *atpIBEFHAGDC* confirm R	AGTGGCGCTGACTGATAAACC
CF *arnBCADTEF* KO F	AAACTTCGGTAGCGTTAAATGGCTTGAAGGAATAATGATGGTGTAGGCTGGAGCTGCTTC
CF *arnBCADTEF* KO R	ATGGGAGCGCCTGTTACGACTGTCGGCAGATAAATGAGCCATATGAATATCCTCCTTAGT
CF *arnBCADTEF* confirm F	TAAACCACTCCCCACTGTCTTTG
CF *arnBCADTEF* confirm R	CAAAAGGTGCGCTATGGAATGG
CF *gmhB* KO F	GTGGCAAAGTCTGTACCCGCAATTTTTCTCGACCGTGACGGTGTAGGCTGGAGCTGCTTC
CF *gmhB* KO R	TGCATTTTTGTTTCAACCGCTCACCTTTTACCCATAACGCATATGAATATCCTCCTTAGT
CF *gmhB* confirm F	CTGTCAGGTACGGTCGAAGCC
CF *gmhB* confirm R	CGGCTGTTTGTGTGAAGTGATTC
CF *ubiH* KO F	GTGACATTGAAGCATTAATGGCTGCGGCGCGACTGCAATGATGTAGGCTGGAGCTGCTTC
CF *ubiH* KO R	TTATCAGGCCTACTTCTTTAATTCGGCCTACAGGTTATATATATGAATATCCTCCTTAGT
CF *ubiH* confirm F	CGGCATTCGTATTGAGGATGACATC
CF *ubiH* confirm R	CCTGAAAATAGGACAATCAAAAACCGTAG
CF *wzxE* KO F	ACGGTAATTTCGACCTTACTCAACTACTTCGCCTGAAATGGTGTAGGCTGGAGCTGCTTC
CF *wzxE* KO R	CGTGAATCAGTACAGTCATGCCCGCCTACGCCAGAGTAACATATGAATATCCTCCTTAGT
CF *wzxE* confirm F	GTGTTCCACTACATCCCGCTG
CF *wzxE* confirm R	CTGGTGTTGAACTGACCGTG
CF *ruvA* KO F	(14)
CF *ruvA* KO R	(14)
CF *ruvA* confirm F	(14)
CF *ruvA* confirm R	(14)
CF *aroC* KO F	ATTACACGCGCACATACACAACACTCATAACGGAGCCGTGGTGTAGGCTGGAGCTGCTTC
CF *aroC* KO R	TTACCAGCGTGGAATATCGGTCTTCACGTCCGCATTTTGCATATGAATATCCTCCTTAGT
CF *aroC* confirm F	TAACGGTATTCTGATTTGTGAAGTCGG
CF *aroC* confirm R	GCCAGCGCCGCAAGTG
CF *pdxA* KO F	AGCGCGCCAGCGCTTACGTTAAAGTTCTGAGCAACTAATGGTGTAGGCTGGAGCTGCTTC
CF *pdxA* KO R	TGATGGACTCGATTATTCATTGGTATTAACAATCATTTTCATATGAATATCCTCCTTAGT
CF *pdxA* confirm F	ACTCCTCTTTCGGTTGGCATC
CF *pdxA* confirm R	GTTCAATGACCGTGAGCTTGTC

CF *apaGH* KO F	ACTATCTGTCAGAAAACGCGCCATCGAAGGAGAGTTAAACGTGTAGGCTGGAGCTGCTTC
CF *apaGH* KO R	GTAGGCCTGATAAGCGTAGCGCCATCAGGCATAAATCTCCATATGAATATCCTCCTTAGT
CF *apaGH* confirm F	CGCCAGCGCCTAAAGTTGATTC
CF *apaGH* confirm R	AGAAGCTGTATCTGACGCACATTG
CF *lpdA* KO F	GACCCGGTGGACGACGGGTATAAATTAAGAGGTCATGATGGTGTAGGCTGGAGCTGCTTC
CF *lpdA* KO R	AAACGCCTCCGAAGAGGCGTTTTCACAAAAGCGGGAAGACATATGAATATCCTCCTTAGT
CF *lpdA* confirm F	TCATGAAGTCAGTGAGGTTATTGTGC
CF *lpdA* confirm R	GTCGCGACAATCATGCCAAC
CF *hscBA* KO F	CCCGTGAACAACAGAGAATCGATGCCCTGAGAATGTTATGGTGTAGGCTGGAGCTGCTTC
CF *hscBA* KO R	AAAATAACAATCTTTGGCATATTAAACCTCGTCCACAGACATATGAATATCCTCCTTAGT
CF *hscBA* confirm F	GGTACGCAGTTAGATTTCGTCAAAG
CF *hscBA* confirm R	CCTTCACGTACGATGCAGTGG
CF *sapABCDF* KO F	TTAGTAAGATAGCCTCTTTATATAATCCATTAAAATTATGGTGTAGGCTGGAGCTGCTTC
CF *sapABCDF* KO R	GATGGCAGAATGCCCCCCGGTTAAGGGGGCAGTCGTTTGCATATGAATATCCTCCTTAGT
CF *sapABCDF* confirm F	CGCGTTTATAGCAAGTCAGATGGG
CF *sapABCDF* confirm R	CAACTTGTCGACGAGGCCAG
CF *prc* KO F	GCAGAACACCTGGTGTTCTGAAACGGAGGCCAGGCCTGGCGTGTAGGCTGGAGCTGCTTC
CF *prc* KO R	TGTTAAAAAAACAGGCACAATTTCTTGTGCCTGTTCGAGGATATGAATATCCTCCTTAGT
CF *prc* confirm F	GGATGCCACCGTATTAGAAATCAC
CF *prc* confirm R	CTTATGACTATTTTCAAGCATCCATAGGT
CF *xerC* KO F	GTTGCCGGAGCTGCTGGAGCGCTGGATTGAACGCGTATGAGTGTAGGCTGGAGCTGCTTC
CF *xerC* KO R	CCAAAGGCCGGTAAAAACGCATTATTTCCCCCGTTTGGCCATATGAATATCCTCCTTAGT
CF *xerC* confirm F	GAATTGCTGGTCATGATGCCGGAG
CF *xerC* confirm R	GGTGACGCCATTGGGTAACATC
CF *proP* KO F	ACTGGAGCGTGACAGGGCTAAGTCTATGAGGACGAGTATGGTGTAGGCTGGAGCTGCTTC
CF *proP* KO R	GTAGGCCTAAAAGCTGCGTGCCATCAGGCAATGACACGACATATGAATATCCTCCTTAGT
CF *proP* confirm F	GGTCATTAACCGCCCAATCAGG
CF *proP* confirm R	GCACACGGTGGTCAAACTTTC
CF *pstSCABphoU* KO F	TCAAACAACGATTTACGAAAACCGTGCAGGAGACATTATGGTGTAGGCTGGAGCTGCTTC
CF *pstSCABphoU* KO R	TGGCGCTAACGCTTATCCGGCCTACTTTCTACGTTTTTACATATGAATATCCTCCTTAGT
CF *pstSCABphoU* confirm F	CCATCCGAATCCCAACGCATC
CF *pstSCABphoU* confirm R	CCGCATAACAAACCGATCGATC
CF *tatC* KO F	(14)
CF *tatC* KO R	(14)
CF *tatC* confirm F	(14)
CF *tatC* confirm R	(14)
