# Supplementary material for: Fitness factor genes conserved within the multi-species core genome of Gram-negative Enterobacterales species contribute to bacteremia pathogenesis
Source: PLoS Pathog. 2024 Aug 23;20(8):e1012495. doi: 10.1371/journal.ppat.1012495 (PMC11376589; doi:10.1371/journal.ppat.1012495)
Supplement: S2 Table — (PDF) [file ppat.1012495.s009.pdf]

Supplemental Table 2. Statistically significant *In vitro* phenotypes of fitness gene mutants.

**Mutant**	***E. coli* CFT073**	***K. pneumoniae* KPPR1**	***C. freundii* UMH14**	***S. marcescens* UMH9**	***E. hormaechei* UM_CRE_14**
apaGH					
arcA	SC[a]	SC	SC	SC	SC
arnBCADTEF	PB	PB, LPS-M	PB	PB, LPS-M	-[b]
aroC	LowSid	LowSid	LowSid	LowSid	
atpIBEFHAGDC					
gmhB	BS				
hscBA					
lpdA					
pdxA					
prc	SS	SS	SS, OS	SS, OS	SS
proP					
pstSCABphoU	AP	AP	AP	AP	
ruvA	BS, CS	BS, CS	CS	CS	CS
sapABCDF					
tatC		BS	BS	PL	
ubiH					
wzxE	SS, PB, OS, BS	SS, OS	PB, BS	SS, PB, OS, BS	SS, PB, OS
xerC	CS	CS	CS	CS	

[a]**Abbreviations**:
SS, human serum sensitivity; **PB**, polymyxin B sensitivity; **OS**, osmotic stress sensitivity; **BS**, bile salt sensitivity; **AP**, alkaline phosphatase overexpression; **LowSid**, mutation reduced siderophore activity; **SC**, small colony morphology; **CS**, ciprofloxacin susceptibility; **PL**, Tat-mediated protein localization; **LPS-M**, LPS modification.
[b]*arn* genes not present in *E. hormaechei* UMCRE14.
